# Spatial and temporal homogeneity of driver mutations in diffuse intrinsic pontine glioma

**DOI:** 10.1038/ncomms11185

**Published:** 2016-04-06

**Authors:** Hamid Nikbakht, Eshini Panditharatna, Leonie G. Mikael, Rui Li, Tenzin Gayden, Matthew Osmond, Cheng-Ying Ho, Madhuri Kambhampati, Eugene I. Hwang, Damien Faury, Alan Siu, Simon Papillon-Cavanagh, Denise Bechet, Keith L. Ligon, Benjamin Ellezam, Wendy J. Ingram, Caedyn Stinson, Andrew S. Moore, Katherine E. Warren, Jason Karamchandani, Roger J. Packer, Nada Jabado, Jacek Majewski, Javad Nazarian

**Affiliations:** 1 Department of Human Genetics, McGill University, Montreal, Québec, Canada H3A 1B1; 2 McGill University and Génome Québec Innovation Centre, Montreal, Québec, Canada H3A 0G1; 3 Research Center for Genetic Medicine, Children's National Health System, Washington, District Of Columbia 20010, USA; 4 Institute for Biomedical Sciences, George Washington University School of Medicine and Health Sciences, Washington, District Of Columbia 20052, USA; 5 Department of Pediatrics, McGill University and McGill University Heath Centre Research Institute, Montreal, Québec, Canada H4A 3J1; 6 Division of Pathology, Children's National Health System, Washington, District Of Columbia 20010, USA; 7 Center for Cancer and Blood Disorders, Children's National Health System, Washington, District Of Columbia 20010, USA; 8 The Department of Neurological Surgery, George Washington University School of Medicine and Health Sciences, Washington, District Of Columbia 20052, USA; 9 Center for Molecular Oncologic Pathology, Department of Medical Oncology, Dana-Farber Cancer Institute, Boston, Massachusett 02115, USA; 10 Department of Pathology, CHU Ste-Justine, Université de Montréal, Montreal, Québec, Canada H3T 1C5; 11 UQ Child Health Research Centre, The University of Queensland, Brisbane, Queensland 4101, Australia; 12 University of Queensland Diamantina Institute, The University of Queensland, Brisbane, Queensland 4102, Australia; 13 Oncology Service, Children's Health Queensland Hospital and Health Service, Brisbane, Queensland 4101, Australia; 14 National Cancer Institute, National Institute of Health, Bethesda, Maryland 20892, USA; 15 Department of Pathology, Montreal Neurological Hospital, McGill University, Montreal, Québec, Canada H3A 2B4; 16 Brain Tumour Institute, Center for Neuroscience and Behavioral Medicine, Children's National Health System, Washington, District Of Columbia, 20010, USA; 17 Department of Integrative Systems Biology, George Washington University School of Medicine and Health Sciences, Washington, District Of Columbia 20052, USA

## Abstract

Diffuse Intrinsic Pontine Gliomas (DIPGs) are deadly paediatric brain tumours where needle biopsies help guide diagnosis and targeted therapies. To address spatial heterogeneity, here we analyse 134 specimens from various neuroanatomical structures of whole autopsy brains from nine DIPG patients. Evolutionary reconstruction indicates histone 3 (H3) K27M—including H3.2K27M—mutations potentially arise first and are invariably associated with specific, high-fidelity obligate partners throughout the tumour and its spread, from diagnosis to end-stage disease, suggesting mutual need for tumorigenesis. These H3K27M ubiquitously-associated mutations involve alterations in TP53 cell-cycle (*TP53/PPM1D*) or specific growth factor pathways (*ACVR1/PIK3R1*). Later oncogenic alterations arise in sub-clones and often affect the PI3K pathway. Our findings are consistent with early tumour spread outside the brainstem including the cerebrum. The spatial and temporal homogeneity of main driver mutations in DIPG implies they will be captured by limited biopsies and emphasizes the need to develop therapies specifically targeting obligate oncohistone partnerships.

## Abstract (web summary)

Diffuse Intrinsic Pontine Gliomas are diagnosed by sampling a small portion of the tumour. Here, using multiple samples from tumours, the authors analyse the spatial and temporal distribution of driver mutations revealing that H3K27M mutations arise first in tumorigenesis followed by a specific invariable sequence of driver mutations, which are homogeneously distributed across the tumour mass.

## Graphical abstract

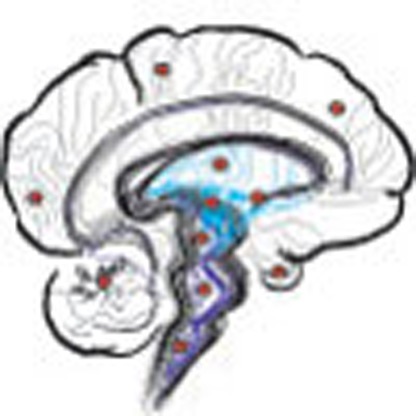

Diffuse Intrinsic Pontine Gliomas (DIPGs) represent 10–15% of all paediatric brain tumours and have a dismal median survival of 9–12 months. They seemingly arise as *de novo* tumours in the pons where surgical options are limited^1^,^2^. Recently, we and others have identified recurrent somatic gain-of-function mutations leading to lysine 27 to methionine (p.Lys27Met, K27M) substitution in histone H3 in ∼80% of DIPGs^2^,^3^,^4^. Despite a low mutational rate relative to other cancers, these H3K27M mutants appear to be often associated with other mutations in cell cycle regulatory genes (*TP53, PPM1D*), chromatin remodeler (*ATRX*) or growth factors (*ACVR1*) (refs 3, 4, 5, 6, 7, 8).

Spatial and temporal tumour heterogeneity is viewed as a major obstacle to accurate diagnosis and successful targeted therapy in cancer. Many adult cancers including high-grade gliomas (HGG) show intra-tumour heterogeneity^9^,^10^,^11^, which confounds determining the complete genetic landscape when only a fraction of a tumour is sampled. In DIPG, published studies have solely used biopsied or limited autopsied specimens without assessing the full spatial and temporal tumour heterogeneity. Despite the fact that H3K27M mutations are identified in most DIPG tumour samples^2^,^3^,^4^,^5^,^6^,^7^,^8^,^12^, the homogeneity of histone and its partner mutation(s) across tumour mass is yet to be determined. It is not currently clear to what extent the tumour may be composed of a mixture of clonal populations carrying distinct mutations. In addition, while DIPGs invariably exhibit tumour spread outside of the pons and brainstem^13^, there are to date no comprehensive molecular studies of these invading cells. Such infiltrating tumour cells may originate from specific clones present in the primary site, as described for other cancers^14^ including other paediatric tumours^15^,^16^. Alternately, malignant cells may seed early during tumour evolution, as recently shown for low-grade gliomas in adults^17^ and/or may genetically diverge from the primary tumour as seen in metastatic cancer including medulloblastoma^16^.

Therefore, in order to define DIPG heterogeneity, we sought to: (i) determine whether the currently recommended needle biopsies^18^ robustly define DIPG tumour biology; (ii) infer the temporal order of specific driver mutations in DIPG; and (iii) fully characterize the molecular signature of the primary, regional and distant subclones, and define their evolutionary relationships. Our results suggest that histone H3K27M mutations arise first and are associated with specific, obligate partners involving alterations in TP53 cell-cycle (*TP53/PPM1D*) or specific growth factor pathways (*ACVR1/PIK3R1*), which are present throughout the tumour as well as its spread outside the brainstem.

## Results

### Genomic landscape of primary and extended tumours

**Figure 1:**
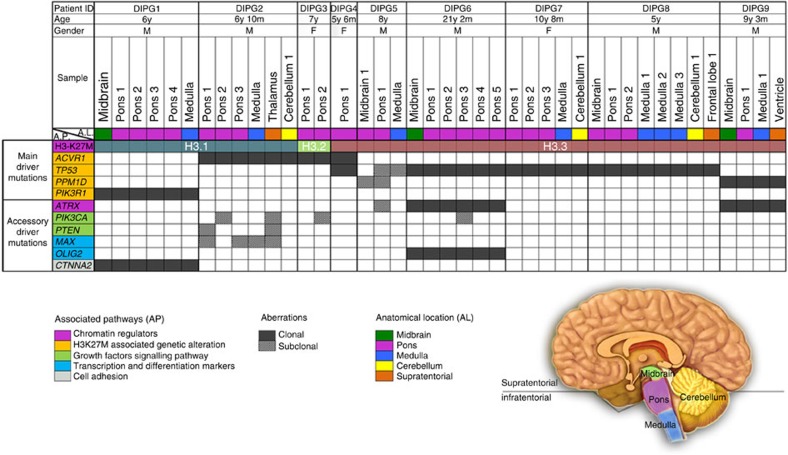
Oncogenic alterations in 41 sub-regions from nine DIPG patients from whole exome sequencing data. Samples representing different anatomical locations within each patient are represented in columns. The mutations (in rows) were selected based on published datasets in paediatric glioblastoma and specifically DIPG. Mutations were divided into two subgroups; driver mutations which are essential for tumour initiation/maintenance and accessory driver mutations, which can further promote and accelerate tumour growth, but are not absolutely essential for tumour initiation or maintenance.

We analysed 134 punch cores from nine DIPG whole brain specimens obtained at autopsy as previously described^19^. Selected punch cores represented multiple spatial regions of the primary tumour and adjacent areas within the brainstem (average of six per autopsy encompassing the pons, midbrain and medulla), cerebellum and various cerebral neuroanatomical regions (average of 10 per autopsy) (Supplementary Tables 1–3; Supplementary Fig. 1). Sixty-seven samples representative of tumour and normal brain areas based on histological assessment were subjected to whole exome sequencing (WES) (Fig. 1; Supplementary Tables 1–4 and Supplementary Data 1–9, Supplementary Fig. 2). Oncogenic mutations associated with HGG^3^,^5^,^6^,^7^,^8^ were identified in 41/67 (61%) of punch cores and a vast majority (35/37; 95%) of punch cores taken from the brainstem (Supplementary Tables 1 and 2). Surprisingly, samples with oncogenic mutations also included distal supratentorial areas (3/20), a few of which were deemed radiologically and histologically normal (Fig. 1, Supplementary Tables 1–3, Supplementary Figs 2A–E and 4). In eight DIPGs, we observed K27M mutations in canonical H3.1 (2/8) or non-canonical H3.3 (6/8). In one patient, we identified K27M in *HIST2H3C*, which encodes the canonical histone 3.2 (H3.2K27M) (Fig. 1) a novel mutation recently described in one paediatric DIPG sample^20^. H3K27M mutations were ubiquitous in all 41 samples with oncogenic content, and were always associated with at least one additional partner driver mutation: *TP53*, *PPM1D*, *ACVR1* or *PIK3R1* (Fig. 1).

### Clonal evolution of diffuse intrinsic pontine glioma

**Figure 2:**
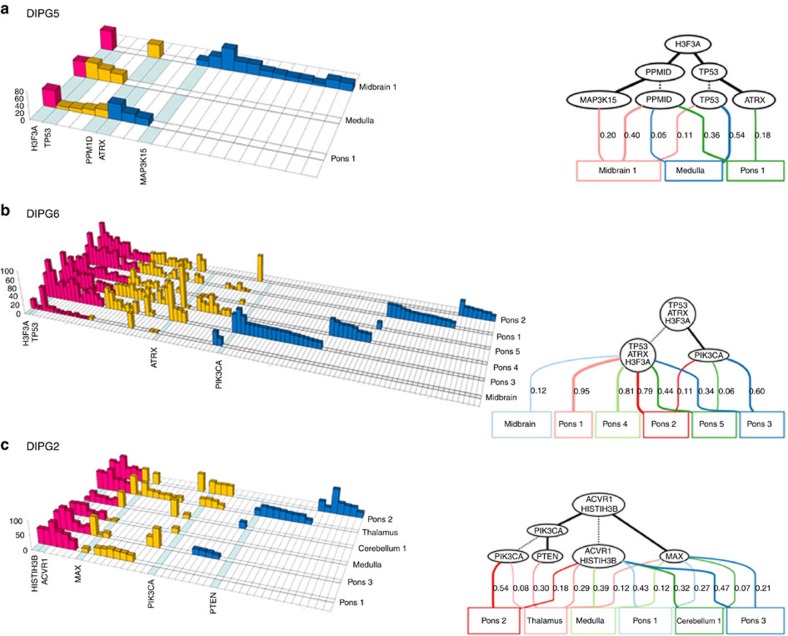
Selected examples of clonal evolution within DIPG tumours. Left: histograms show the raw allele frequencies (whole exome sequencing data) for each somatic mutation in different autopsy regions within each tumour. Red: ubiquitous mutations across regions; yellow: mutations shared in at least two regions; blue: mutations seen in only one region. Right: phylogenetic trees constructed from the mutation allele frequencies of deep amplicon sequencing data showing the order of evolution along with support probabilities (upper portions of graphs) and clonal mixing proportions within samples (lower portions). For clarity, only mutations selected to be likely oncogenic are shown. (**a**) DIPG5: a rare case harboring both TP53 and PPM1D mutations, which are generally found to be mutually exclusive. PPM1D and TP53 mutations occur in distinct clones and are both secondary to H3K27M. ATRX is also secondary and subclonal. (**b**) DIPG6: while it is impossible to resolve the order of H3/TP53/ATRX mutations' appearance, PIK3CA is clearly sub-clonal and appears in the later stages of evolution within this tumour. (**c**) DIPG2: the H3.1 K27M and ACVR1 main driver mutations are ubiquitous, occur at similar frequencies across all samples, and their mutation order cannot be resolved. Conversely, other accessory driver mutations are clearly secondary in order of appearance, and are present only in distinct subclones.

To decipher clonal evolution of DIPG, we employed a strategy resembling previously successful multi-region tumour sequencing studies^14^. Such approaches aim to reconstruct evolutionary relationship among multiple samples from the same patient based on the presence or absence of newly emerging somatic mutations^14^,^17^, and the relative mutation allele frequencies^21^,^22^. We used our WES data (70 × mean coverage) to select mutations—based on their presence in our and other available DIPG datasets^3^,^5^,^6^,^7^,^8^—for further screening using deep amplicon sequencing (∼4,000 × mean coverage) in the initial sample set and in other geographically proximal and distal samples (*N*=134). Mutation allele frequencies were corrected for aneuploidy and presence within copy number variant (CNV) regions whenever possible, and subsequently used to reconstruct evolutionary trajectories (Fig. 2; Supplementary Figs 5, 6 and 7; Supplementary Table 5). Remarkably, evolutionary analysis showed obligate association of specific combinations of at least four primary partner driver genes in our sample set: H3K27M/TP53 and/or PPM1D, H3K27M/ACVR1 and H3K27M/PIK3R1 (Fig. 2; Supplementary Fig. 7). The association between the histone mutation and obligate partner(s) was invariably maintained throughout all samples from an autopsy brain. This is in striking contrast to adult cancers, including HGG, which have large numbers of mutations and potentially long evolutionary histories resulting in complex clonal structures^9^,^10^,^17^. This also contrasts isocitrate dehydrogenase (IDH)-mediated tumorigenesis, where IDH mutations are associated with genetic alterations that change over the course of the disease either through partnership with a different mutated gene or through acquisition of additional/different mutations in the same gene during tumour evolution^17^.

### Temporal tumour homogeneity of main driver mutations

The tight association of principal driver mutation pairs implies early and nearly concurrent appearance during tumour evolution. Accordingly, in several cases the order of the partner mutations could not be resolved (Fig. 2; Supplementary Fig. 7). However, variations in the relative allele frequencies across spatial samples in DIPG1, DIPG5, DIPG7 and DIPG9 enabled estimation of the most likely mutation order, suggesting H3K27M as the earliest tumorigenic event in DIPG (Fig. 2, Supplementary Fig. 7). A clear example of such clonal evolution is illustrated by DIPG5 (Fig. 2a) harboring three principal driver mutations (H3.3K27M/*TP53*/*PPM1D*). PPM1D regulates TP53 cell-cycle checkpoint and mutations in both genes have been described as mutually exclusive in gliomas^8^,^23^. DIPG5 is the sole reported exception to this rule, providing valuable evolutionary insights. We identified two major clones carrying distinct combinations of driver mutations, H3.3K27M-*TP53* and H3.3K27M-*PPM1D*, indicating the H3.3K27M mutation as the initial event, followed by *TP53* mutation in one subclone, and *PPM1D* mutation in another. This suggests that the early presence of oncohistone mutations promotes subsequent alterations of the TP53 pathway, and that this partner association—which is required for full transforming potential in DIPG—is subsequently maintained throughout the tumour.

Further analysis of combined data from our cohort and published genomic DIPG datasets (*N*=130) showed the presence of H3K27M in 116/130 (89%) of patients (29 H3.1K27M, 1 H3.2K27M, 86 H3.3K27M, Supplementary Table 6 and Supplementary Fig. 10). H3K27M mutants were in vast majority of cases (111/116) associated with partner mutations, which included the *TP53* pathway exclusively (71), growth factor pathways exclusively (28), or a combination of both (11). Mutations in *TP53* (64), *PPM1D* (9), *TP53* and *PPM1D* (1, DIPG5), or other genes controlling this cell-cycle checkpoint (1) were preferentially associated with H3.3K27M. Recurrent activating *ACVR1* somatic mutations were identified in 32 H3K27M samples, including eight with *TP53* pathway mutations. These mutations, as previously shown, tend to favor association with the canonical H3K27M (25/32). In rare cases including one from our dataset (DIPG1), we identified H3K27M/*PIK3R1* association as the main combination of driver mutations (3/111) (Supplementary Table 6 and Supplementary Fig. 10). A novel *CTNNA2* alteration was also identified as clonal in DIPG1 similar to PIK3R1. Even if we cannot fully rule out its contribution to tumorigenesis, it was not viewed as main driver as no clear phenotypic advantage could be inferred. Furthermore, this mutation has not been previously reported in COSMIC and was absent in other DIPG samples including other datasets analysed in this study (Fig. 1, Supplementary Table 7).

In order to assess the fidelity of postmortem specimen as representative of newly diagnosed tumours, we analysed three available paired biopsy/autopsy samples (Supplementary Table 7). In all cases, H3K27M mutants and their obligate partners were present both at diagnosis and at the end-stage of the disease. This finding, along with the analysis of published diagnosis biopsy datasets^3^,^5^,^6^,^7^,^8^, indicates that the H3K27M obligate partnership is maintained throughout the disease course.

### Molecular characterization of tumour spread

**Figure 3:**
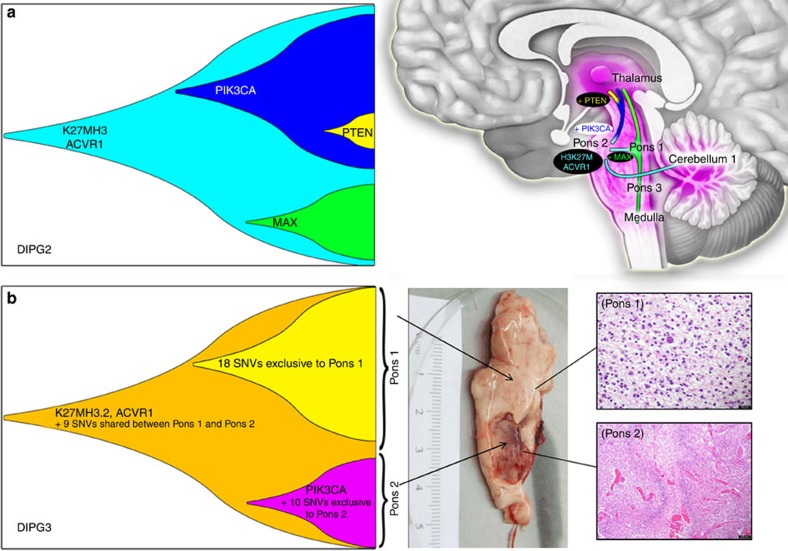
Tumour spread in DIPG. (**a**) Tumour spread in DIPG2 in the thalamus, cerebellum and brainstem. Tumour extension in the thalamus harbors secondary mutation PIK3CA, MAX and PTEN, which indicates late spread from both Pons 1 and Pons 2. Extension towards the cerebellum is relatively early in the tumour evolution as it lacks secondary mutations found in the primary tumour and other brainstem spread. (**b**) Evolution of tumour in patient DIPG3. Autopsy revealed two morphologically and histologically distinct regions of the tumour, indicated as DIPG3 Pons 1 (low-grade) and DIPG3 Pons 2 (high-grade). Exome sequencing identified 11 SNVs and several large scale CNAs common to both regions. Shared alterations included H3.2 K27M and ACVR1 G328V mutations that are likely the main driver mutations in this patient. The analysis also indicated a clear clonal substructure of the two regions, with 18 SNVs and 1 CNA found only in DIPG3 Pons 1, and 11 SNVs and 3 CNAs unique to DIPG3 Pons 2. Intriguingly, DIPG3 Pons 2 carries the activating PIK3CA H1047R mutation, which occurs early in the evolution of this sub-clone judging by its high allelic frequency. PIK3CA H1047R is associated with multi-potency^24^ and PI3K activation with angiogenesis and growth and this mutation likely contributes to tumour aggressiveness and high-grade features of Pons 2 compared with Pons 1 in DIPG3. Scale bar=500 μm.

Molecular studies of punch cores representing various neuroanatomical locations showed tumour extension outside the brainstem (Supplementary Tables 1–3; Supplementary Fig. 3). Remarkably, tumour spread was detected in proximal (for example, the cerebellum, 4/8 patients with sampled material) and distal (thalamus 4/4, hippocampus/ventricle/frontal or occipital lobes 3/7) brain locations, and in rare cases in areas with no clinical, radiological or histopathological evidence of tumour (Supplementary Figs 2, 3, 4). Interestingly, re-analysis of the published datasets showed two cases where the matched ‘normal' frontal lobe samples had H3K27M/partner mutation frequencies similar to the primary tumour^5^. Furthermore, clustering analysis of our DNA methylation data showed distant tumour spread to be more similar to H3K27M mutant tumours than to normal brain (Supplementary Fig. 8). Thus, molecular designation of histologically ‘normal' brain DIPG specimen warrants more in-depth molecular scrutiny. Evolutionary studies of tumour dissemination were consistent with a mixture of early and late tumour spread. Some sites harbored most of the secondary mutations observed in the primary tumours suggestive of late spread, while others lacked the secondary mutations found in the primary tumour site suggestive of early spread (Fig. 3a, Supplementary Fig. 9).

In addition to the main driver mutations discussed above, several patients carry recurrent mutations that are clearly subclonal (present in some but not all tumour areas in a patient) and occur at later stages of tumour evolution (Fig. 1). These include mutations in the chromatin remodeler ATRX (DIPG5 and DIPG9), as well as the activating PIK3CA H1047R mutation^24^, which affects the catalytic domain of PIK3CA (DIPG2, DIPG3 and DIPG6) (Fig. 1). In DIPG3 gross visual inspection of the pons indicated two morphologically distinct areas (Pons 1 and 2, Fig. 3b). Histological examination identified area Pons 1 as low-grade infiltrative astrocytoma (WHO II) and Pons 2 as high-grade astrocytoma (WHO IV, glioblastoma, Fig. 3b). Both areas carried *HIST2H3C*/*ACVR1* mutations inferred to occur early in tumour evolution (Fig. 3b). However, only Pons 2 (WHO IV) carried PIK3CA H1047R. RNAseq analyses showed enrichment of overexpressed genes involved in vasculature development and angiogenesis pathways in Pons 2, in keeping with prior studies linking PI3K activation to angiogenesis^25^ (Supplementary Data 10). Alterations affecting the PI3K pathway are prevalent both in our sample set (4/9) and in published DIPG datasets (31/116, 26%). Our data suggests that PIK3CA mutations are not essential to tumour initiation in DIPG but may confer localized survival, growth and angiogenic advantage in the areas they are subsequently acquired.

## Discussion

Genomic analysis of autopsy DIPG brains provides a fascinating opportunity to reconstruct the evolution of this deadly tumour, and has important implications on potential therapeutic interventions. Despite the sample number limitation based on disease incidence, this study provides insight into the spatial and temporal evolution of the tumour genome in DIPG helping derive a general model for this disease.

Our analysis suggests that H3K27M mutations are the initial oncogenic event in DIPGs. However, these mutations are not solely sufficient for tumour formation, as they require, nearly universally, obligate partners for effective tumorigenesis. There is some disagreement in the cancer field as to the definition of ‘driver' versus ‘passenger' mutations. The designation passenger has been used for any variant that is not absolutely essential to the tumorigenesis. However, for the purpose of this study—and more generally—we postulate three categories of mutations: (1) main driver mutations are essential to initiate and continue tumorigenesis; (2) accessory driver mutations can further promote and accelerate tumour growth but are not absolutely essential; (3) passenger mutations are neutral and do not affect the tumour. H3K27M and their obligate partners appear thus to be the main driver mutations, since they always co-occur together throughout each tumour.

These K27MH3.3 partner mutations are mostly genetic alterations affecting the TP53 pathway, mainly *TP53* and less frequently *PPM1D* genes. H3K27M mutants also favor association with *ACVR1* gain of function mutations and in rare cases (1 in our dataset and 2 in other reported cases) *PIK3R1* mutations. Interestingly, even if more frequent, PIK3CA mutations, including PIK3CA H1047R, clearly fall into the accessory driver category, since they were invariably sub-clonal in our dataset and in some other reported cases. Similarly, a PTEN mutation was also found in some but not all tumour areas in DIPG2 (Fig. 3). We can only speculate at this time as to why mutations that ultimately activate the PI3K/AKT pathway appear clonal for some of the component genes and subclonal for others. This may be related to the dosage effect of pathway activation, as the loss of the regulatory unit (*PIK3R1*) leads to increased baseline activity of the wild-type kinase whereas the PIK3CA H1047R mutation has been associated with higher kinase activity and oncogenic potential through cellular reprograming and induction of stemness properties^24^. Further studies are needed to help ascertain the specific role of these accessory driver mutations in oncohistone tumorigenesis.

Once acquired, the main driver partnership is maintained throughout the course of the disease (diagnosis to autopsy), in all cells across the primary tumour site, and in tumour spread throughout the brain. The obligate partner is not chosen randomly. One speculation about the phenotypic advantage of the gain of additional mutations may be that the partners are selected as those best suited to increase levels of mutant H3 in cells. Indeed, H3K27M mutants show a dose-dependent effect in inducing cellular proliferation^26^,^27^. Canonical H3 variants are the most abundant histones in cells and need cell-cycle division (S-phase) for synthesis, whereas non-canonical H3.3 variants represent ∼5% of all H3 in cells and are present throughout the cell cycle. Accordingly, H3K27M mutations in cell cycle-dependent canonical histones may co-occur with alterations affecting ACVR1, a growth factor that induces cell division, resulting in the synthesis of wildtype and mutant H3.1 proteins. H3K27M mutations in the non-canonical H3.3 histone are mainly associated with mutations affecting the TP53 pathway, as these possibly offer evasion from cell death and senescence and provide the needed opportunity for the mutant H3.3K27M to exert its effect over a longer period, effectively reshaping the epigenome and drive tumour formation.

The previously unsuspected homogeneity for main driver mutations across the course of the disease uncovered in this study indicate that efforts to cure DIPG should be directed at the oncohistone partnership, as other genetic alterations are generally sub-clonal. Our findings further indicate that needle biopsies recommended to orient care are representative of the main drivers in DIPG even if the regional heterogeneity of other secondary targetable alterations, such as PIK3CA mutations, may not be fully captured. Based on early tumour spread, efforts to cure DIPG should aim for early systemic tumour control as opposed to regimens focused on the pons.

## Methods

### Patient samples

All patient samples were collected with informed consent in accordance with the respective Ethics Review Boards of the Children's National Health System, Washington, DC, USA and the University of Queensland Child Health Research Center, Brisbane, Australia. DIPG post-mortem specimen procurement was performed as previously described^19^. Briefly, the brainstem and cerebellum were removed *en bloc* from the whole brain, and dissected into ∼9 transverse sections. The cerebral cortex was dissected into ∼11 coronal sections. The brainstem, cerebellum and cerebral cortex sections were alternatively frozen or fixed in formalin. A total of 158 samples were studied by immunohistochemistry and molecular analyses, representing various neuroanatomical locations such as frontal, parietal, temporal, occipital lobes, thalamus, lateral ventricles, hippocampus, midbrain, pons, medulla and cerebellum (Supplementary Tables 1–3; Supplementary Fig. 1). For molecular studies (RNA and DNA) 134 core punches were obtained using a biopsy punch and plunger (2 mm, #33-31, Integra Miltex, York, PA). All histological sections were reviewed by neuropathologists (C.-Y.H. and J.K.), according to the World Health Organization (WHO) classification of tumours. Demographical, clinical and histopathological characteristics of all specimens are presented in Supplementary Table 1.

### Immunohistochemistry

Immunohistochemistry was performed on 5 μm thick formalin fixed paraffin embedded (FFPE) slides. Briefly, slides were de-paraffinized, processed for epitope retrieval, DAB detected using reagents customized for the Leica BOND-MAX automated stainer (Leica Biosystems, Buffalo Grove, IL). Processed slides were probed by immunohistochemical assay for hematoxylin and eosin (H&E), Ki67, H3-K27M and H3-K27me3 as previously described^12^ (Supplementary Fig. 2).

### Antibodies

Rabbit monoclonal anti-Ki67 (Biocare Medical, Concord, CA) were pre-diluted and ready to use. Rabbit polyclonal anti-H3K27M (#ABE419 Millipore, Billerica, MA, 1:500), rabbit monoclonal anti-H3K27me3 (C36B11, #9733 Cell Signaling, Beverly, MA 1:75) were diluted in Bond primary antibody diluent (#AR9352 Leica Biosystems, Buffalo Grove, IL). Secondary detection was conducted using the Bond polymer refined detection kit (Leica Biosystems, Buffalo Grove, IL). Slides were counterstained for hematoxylin nuclear stain.

### RNA and DNA extractions

Frozen tissue samples were homogenized with Trizol, and nucleic acids were phase separated using chloroform. Total RNA was extracted according to the PicoPure RNA Isolation kit (Arctrus Bioscience Inc., Mountain view, CA). Genomic DNA was extracted from frozen tissue using the Gentra Puregene DNA extraction kit, or from FFPE tissue using the QiaAmp DNA mini kit according to the manufacturer's instructions (Qiagen, Valencia, CA). All DNA quantifications were conducted using the Quant-iT Picogreen dsDNA assay kit (Life Technologies, Carlsbad, CA).

### Droplet digital PCR

Digital droplet PCR (ddPCR) assays were performed according to standard methods. Briefly, each 20 μl reaction contained 1 × ddPCR Supermix for Probes (Bio-Rad), 900 nM gene specific HPLC-purified forward and reverse primer, 250 nM gene-specific mutant or wild-type locked nucleic acid (LNA) probe and 12.5–25.0 ng genomic DNA. Each reaction was mixed with 60 μl Droplet Generation Oil (Bio-Rad), partitioned into ∼12,000–16,000 droplets in QX100 Droplet Generator (Bio-Rad), transferred to a 96-well plate and sealed. The primers and probes were designed by Integrated DNA Technologies as follows: forward primer for *H3F3A*: 5′- GTACAAAGCAGACTGCCCGCAAAT -3′, reverse primer; 5′- GTGGATACATACAAGAGAGACTTTGTCCC -3′. Forward primer for *HIST1H3B:* 5′- ACAGACGTCTCTGCAGGCAAGC -3′, reverse primer 5′- GGCGGTAACGGTGAGGCTTT -3′. *H3F3A* K27M wild-type probe (HEX) CA+C+T+C+T+T+GC and mutant probe (FAM) CA+CT+C+A+T+GCG. *HIST1H3B* K27M wild-type probe (HEX) T+CGC+A+A+GAG+CG and mutant probe (FAM) TCGC+A+T+G+AGCG. The PCRs were performed in a T100 Thermal Cycler (Bio-Rad) with the following cycling conditions: 1 × (95 °C for 10 min), 50 × (95 °C for 30 s, 53 °C or 57 °C for 60 s, with 2 °C  s^−1^ ramp rate and 1 × (98 °C for 10 min). Following end-point amplification, the fluorescence intensity of individual droplets was measured with the QX100 Droplet Reader (Bio-Rad) and data analysis was performed with QuantaSoft droplet reader software (Bio-Rad). Positive and negative droplet populations were detected either automatically or manually on two-dimensional graphs and target DNA concentrations were calculated using the Poisson statistics^28^.The absolute transcript levels were initially computed as copies per μl PCR for both mutant and wild-type and then presented as per cent of total gene copy.

### Whole-exome sequencing

Genomic DNA was extracted from multiple post-mortem samples patient using the standard extraction methods as described by Qiagen. Nextera Rapid Capture Exome kit was used to prepare the paired-end libraries according to the manufacturer's instructions using on average 36 ng of total starting genomic DNA. Sequencing was performed on Illumina HiSeq 2000 using rapid-run mode with 100 bp paired-end reads. Next, adaptor sequences were removed; reads were trimmed for quality using the FASTX-Toolkit (http://hannonlab.cshl.edu/fastx_toolkit/). An in-house program was used to ensure the presence of exclusively paired-reads to be used in further steps of the analysis. We next aligned the reads using Burrows-Wheeler Aligner (BWA) 0.7.7 to hg19 as reference genome. Indel realignment was performed using the Genome Analysis Toolkit (GATK)^29^ (http://www.broadinstitute.org/gsa/wiki/). We next marked the duplicate reads using Picard (http://picard.sourceforge.net/) and excluded them from further analyses as previously described^6^. The coverage of consensus coding sequence (CCDS) bases was assessed using GATK. The average coverage over all the samples was 70 × . The majority of samples had >90% of CCDS bases covered by at least 10 reads and >83% of CCDS bases covered by at least 20 reads.

We called SNVs and short indels using SAMtools (http://samtools.sourceforge.net/) mpileup with the extended base alignment quality adjustment (–E)^30^,^31^. Next we filtered them for quality so that at least 10% of reads supporting each variant call. We used both ANNOVAR^32^ and in-house tools to annotate the variants and to identify whether these variants affect protein-coding sequence and if they had previously been observed in datasets including the 1,000 Genomes Project data set (November 2011), the National Heart, Lung and Blood Institute (NHLBI) Grand Opportunity (GO) exomes or in ∼3,000 exomes previously sequenced at our center. Results of whole exome sequencing are summarized in Supplementary Table 4 and presented for individual patients as follows: DIPG1 in Supplementary Data 1; DIPG2 in Supplementary Data 2; DIPG3 in Supplementary Data 3; DIPG4 in Supplementary Data 4; DIPG5 in Supplementary Data 5; DIPG6 in Supplementary Data 6; DIPG7 in Supplementary Data 7; DIPG8 in Supplementary Data 8; DIPG9 in Supplementary Data 9.

### Exploratory targeted high-depth DNA sequencing of hotspot mutations

Genomic DNA from DIPG samples were used for high-depth sequencing using Illumina MiSeq platform. The MiSeq panel covers exons in 16 Histone H3 isoforms (10 H3.1, 2 H3.2 and 3 H3.3 genes) and covering hotspot mutations such as *IDH1* mutation (codon 132), *IDH2* (codons 140 and 172), *ACVR1* (exons 6–9) and *BRAF* (exons 11 and 15) and *PPM1D* (exon 6). Genomic DNA samples were sequenced using the MiSeq sequencing platform (Illumina) as previously described^6^ with an average coverage of >20,000 × of the analogous K27M base change across the three histone variants.

To estimate allele frequency of mutations identified using whole exome sequencing genomic DNA from the same samples was also used for high-depth sequencing on the MiSeq platform with an average coverage of >4,000 × . Reads were mapped to the reference genome (human hg19) using the BWA genome alignment^30^,^31^. Alignment files were fed to an in-house program to calculate different variations' allele frequencies at the desired positions.

### RNA sequencing

We used Qiagen RNeasy Lipid Tissue Mini kit to extract RNA from tumour DIPG3 (Pons 1 and Pons 2) according to manufacturer's instructions. Library was prepared using rRNA depletion methods according to instruction from Epicentre (manufacturer) to achieve greater coverage of mRNA. Paired-end sequencing was performed on the Illumina HiSeq 2000 platform.

### DNA methylation analysis

Methylation profiling data was analysed as previously described^6^. The raw data were subject to quality control and preprocessing utilizing the R package minfi, and normalized for technical variation between the Infinium I and II probes using the SWAN method. We removed probes on sex chromosomes (chrX, Y), those containing SNPs (dbSNP: http://www.ncbi.nlm.nih.gov/SNP/) as well as non-specific probes that bind to multiple genomic locations. Unsupervised hierarchical clustering was performed using average linkage and Pearson rank correlation distance on the top 5,000 most variable probes selected based on standard deviation of beta values (*β*-values).

### Copy number variation analysis

To study copy number variations in our samples we developed an in-house program to calculate the deviation of B allele frequency from 50% as well as normalized coverage from whole exome sequencing data (adapted from methods used in FishingCNV^33^ and ExomeAI^34^). Different CNV events (duplication, deletion and copy neutral LOH) were called based upon the B allelic imbalance and the status of the normalized coverage as follow: deviation from 50% B allele frequency and an increase in normalized coverage was considered as amplification, deviation from 50% B allele frequency and decrease in normalized coverage as deletion and deviation from 50% B allele frequency and no change in the normalized coverage was considered as potential copy neutral loss of heterozygosity. We mainly assessed the CNV events at the chromosomal arm level. The results of our CNV detection are presented in Supplementary Fig. 5 and Supplementary Table 14.

### OncoScan verification of the CNV events

OncoScan FFPE Assay Kit, provided by Affymetrix, is a platform based on Molecular Inversion Probe technology, used to asses copy number and loss of heterozygosity using small amounts of DNA from FFPE samples. We performed this assay on several samples in order to verify our WES based CNV detection method. Genomic DNA was quantified using Picogreen protocol (Quant-iT PicoGreen dsDNA Products, Invitrogen, P-7589) and read on SpectraMAX GeminiXS Spectrophotometer. The OncoScan FFPE Assay Kit was used according to the manufacturer's instructions (Affymetrix). A GeneAmp PCR system 9700 Thermal Cycler (Life Technologies) was used from the anneal stage to the denaturation stage. QC gels of the PCR and HaeIII digest products were performed on E-Gel 48.4% agarose gels using Mother E-Base device (Life Technologies) and imaged with SYNGENE GeneGenius Bio Imaging System (Syngene). The digest DNA target was hybridized on OncoScan array (Affymetrix) and incubated at 49 °C in the Genechip Hybridization oven 640 (Affymetrix) for 17 h at 60 rpm. OncoScan arrays were then washed in a GeneChips Fluidics Station 450 (Affymetrix) using OncoScan stain and wash reagents according to the manufacturer's instructions (Affymetrix). The microarrays were finally scanned on a GeneChip scanner 3,000 (Affymetrix). Data QC analysis was performed with the OncoScan Consol 1.2.0.50 software (Affymetrix) using OncoScan Analysis Library files r1.1. OncoScan positive and negative control supplied in the OncoScan FFPE Assay Kit were used for internal controls to assess the performance of each run. CNV events were called using the normalized data using Nexus Express for OncoScan 3.1 (Affymetrix). The OncoScan plots are represented in Supplementary Fig. 6.

### Constructing evolutionary trees

We used PhyloWGS^35^ to reconstruct the tumour phylogeny, which uses a Bayesian approach to infer cellular frequencies from mutation allele frequencies. It applies Dirichlet process to cluster mutations with similar cellular frequencies and the tree-structured stick-breaking process to model the clonal evolutionary tree. For multi-region samples from the same patient, we normalized the read counts used for phylogenetic tree construction by copy number counts from CNV analysis. Read counts were corrected for the CNV events (Supplementary Fig. 5; Supplementary Table 3) for each sample as follow: in case of duplication Ref′=Ref, Alt′=Alt/2; in case of deletion Ref′=Ref+Alt, Alt′=Alt; in case of a gene on chromosome X Ref′=Ref × 2+Alt, Alt′=Alt; in case of Copy Neutral LOH Ref′=Ref+Alt/2 and Alt′=Alt/2. The reconstructed trees were redrawn in Graphviz by using in-house scripts adapted from AncesTree^36^ to show the trajectories of mutations with contribution ⩾0.05.

### Differential expression and gene set enrichment analysis

We aligned RNASeq data from DIPG3-Pons 1 and DIPG3-Pons 2 (RNA extraction and sequencing protocol described before) using STAR 2.3.0 (ref. 37), and analysed differentially expressed genes using DeSEQ2 (ref. 36). The top 200 over expressed and top 200 under expressed genes in DIPG3-Pons 2 (the sub-clone with PIK3CA activating mutation) were analysed for geneset enrichment using both AmiGo 2 tool^38^ (http://amigo.geneontology.org/amigo) provided by Gene Ontology and DAVID^39^ (https://david.ncifcrf.gov/). We used PANTHER Overrepresentation Test (release 20,150,430) for analysis type by Amigo 2 (annotation version and release date: GO Ontology database released 2015-08-06). The top pathways found by AmiGo 2 to be enriched with enrichment folds higher than 5 and (Bonferroni<0.05) were retained. We used functional annotation clustering and set the stringency to the highest in DAVID and filtered the results for enrichment folds higher than 5 (Bonferroni corrected *P value*<0.05). We used the set of genes with at least 50 RNASeq reads in both DIPG3 Pons1 and 2 combined as background gene set in this analysis (Supplementary Data 10).

## 

## Additional information

**Accession codes:** The whole-exome sequencing data for all tumours (along with RNA sequencing data for DIPG3), have been deposited in the European Genome Archive (EGA) database under the accession code EGAS00001001654. The DNA methylation profiles have been deposited in the Gene Expression Omnibus (GEO) database under the accession code GSE77353.

**How to cite this article:** Nikbakht, H. *et al*. Spatial and temporal homogeneity of driver mutations in diffuse intrinsic pontine glioma. *Nat. Commun.* 7:11185 doi: 10.1038/ncomms11185 (2016).

## Supplementary Material

Supplementary Information Supplementary Figures 1-9 and Supplementary Tables 1-6
