# Supplementary material for: Spatial and temporal homogeneity of driver mutations in diffuse intrinsic pontine glioma
Source: Nat Commun. 2016 Apr 6;7:11185. doi: 10.1038/ncomms11185 (PMC4823825; doi:10.1038/ncomms11185)
Supplement: Supplementary Information — Supplementary Figures 1-9 and Supplementary Tables 1-6 [file ncomms11185-s1.pdf]

Supplementary Figure 1

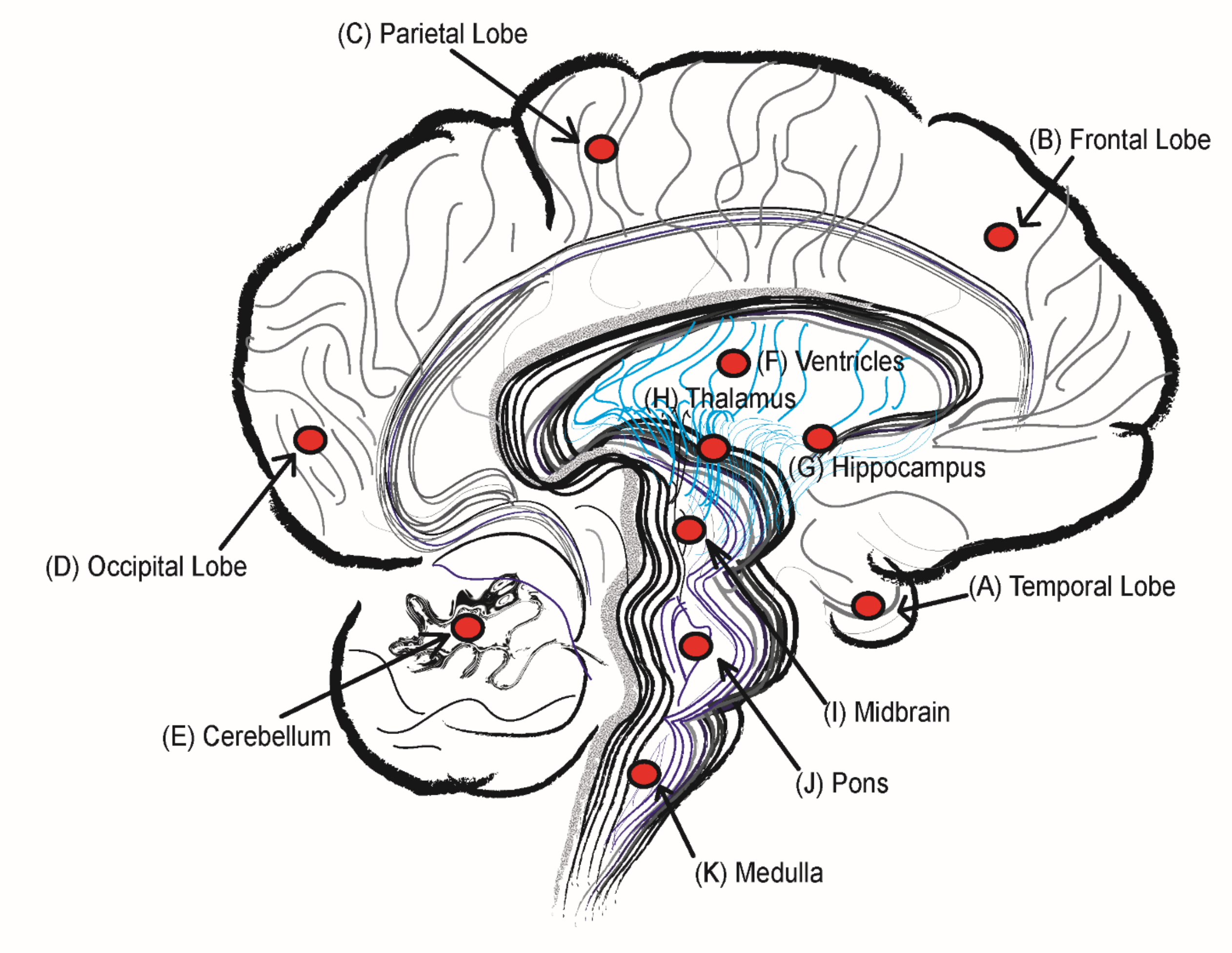

Supplementary Figure 1. Core punch sampling of whole autopsy brains. An average of 6 core punch samples from right and left brainstem (midbrain, pons, medulla) and an average of 10 core punch samples from right and left cerebellum and cerebrum (frontal lobe, parietal lobe, temporal lobe, occipital lobe, lateral ventricles, thalamus, hippocampus) were collected for molecular analyses from frozen and formalin fixed paraffin embedded (FFPE) whole autopsy brains from nine DIPG patients (N=134).

Supplementary Figure 2

(a) **DIPG1 (21y 2m; Male; H3.1 K27M)**

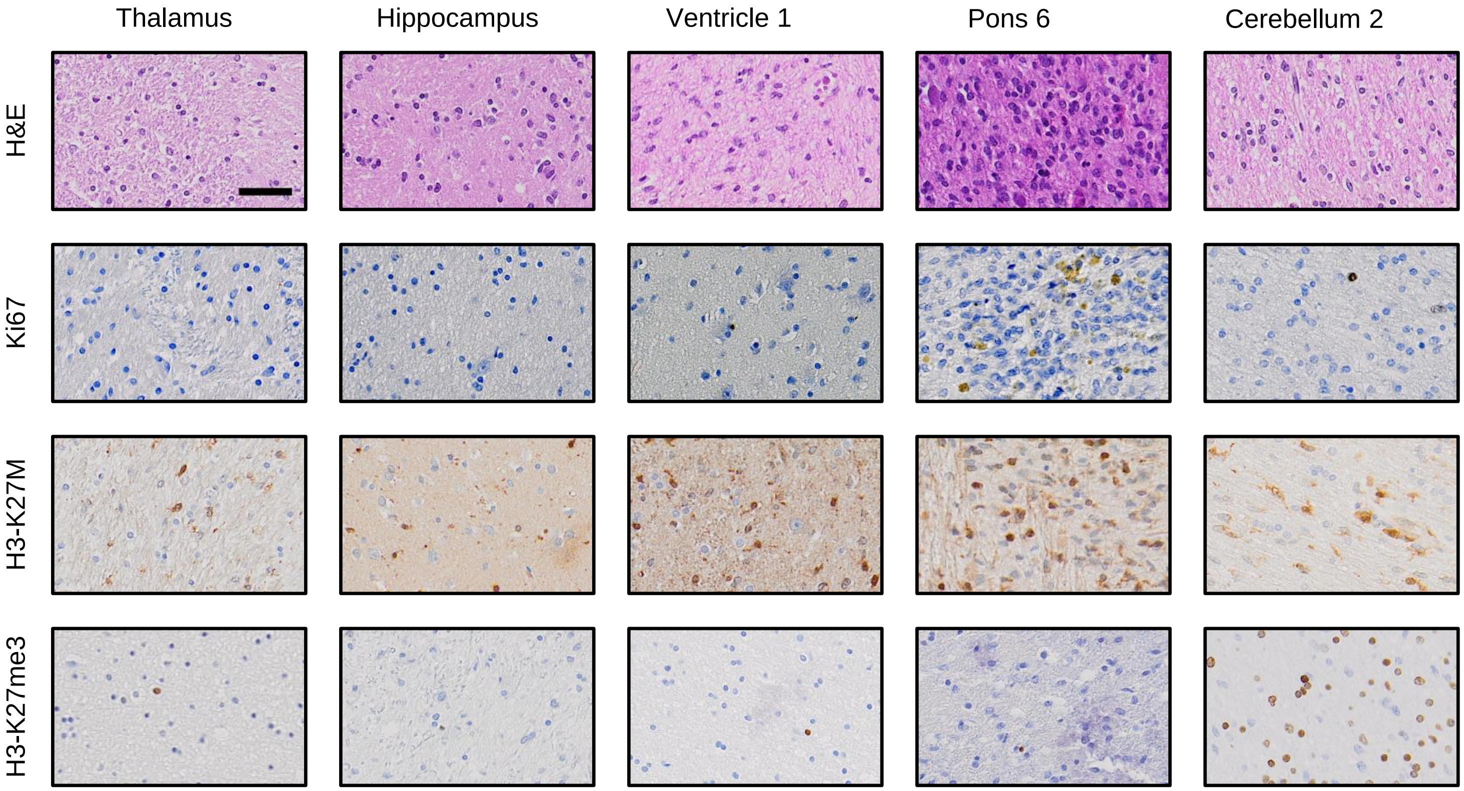

(b) DIPG2 (6y 10m; Male; H3.1 K27M)

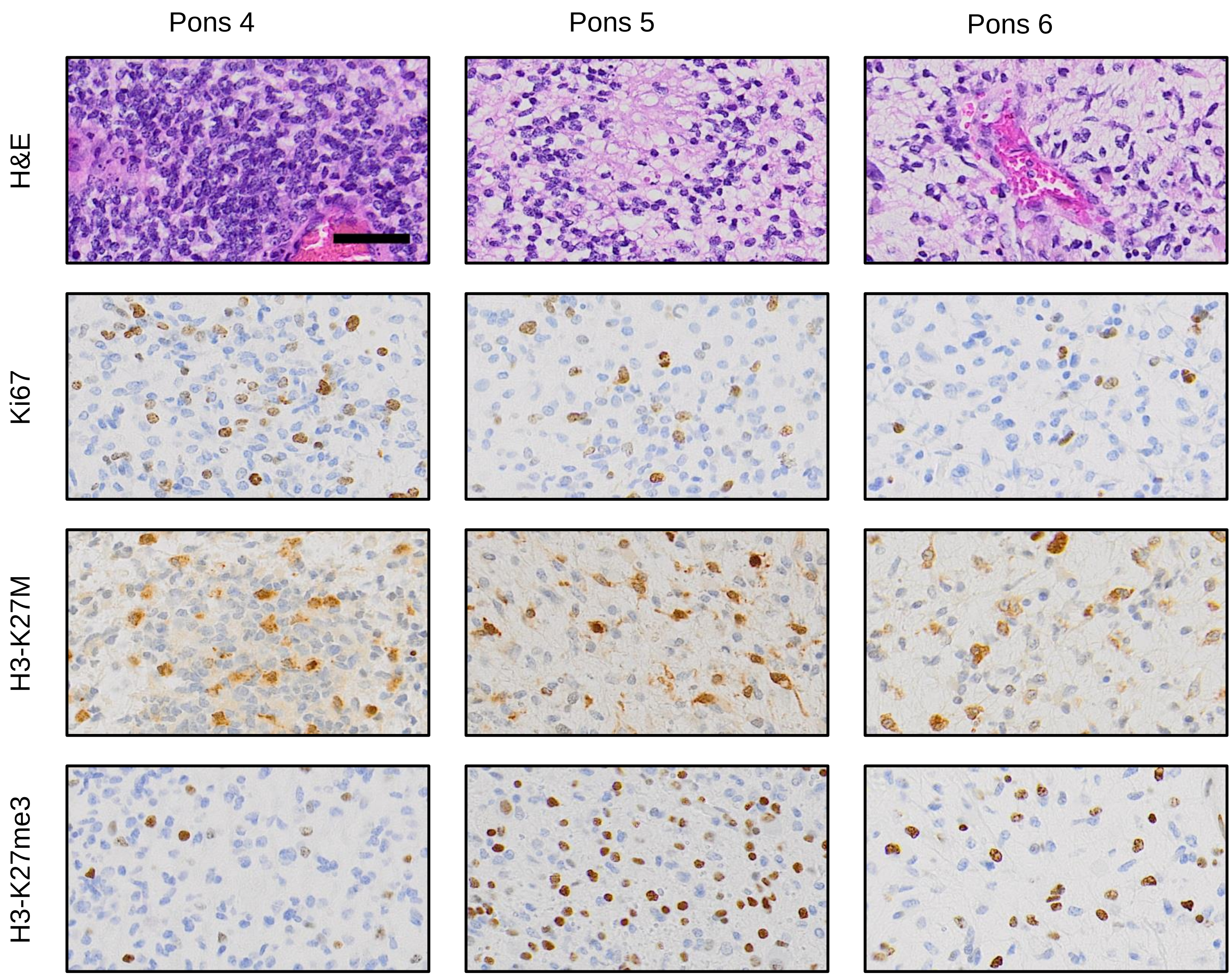

(c) DIPG7; 10y 8m; Female; H3.3 K27M

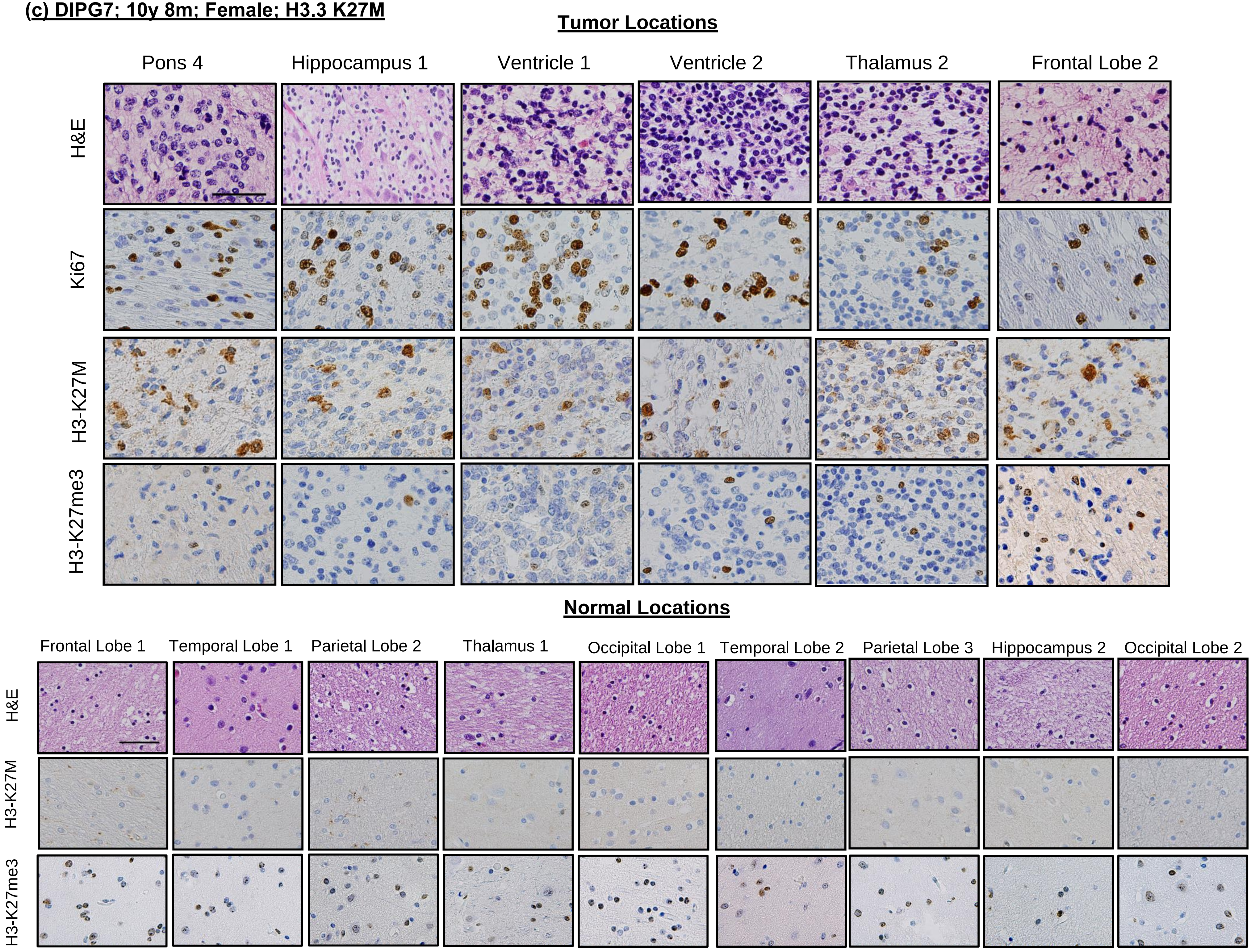

(d) DIPG8 (5y; Male; H3.3 K27M)

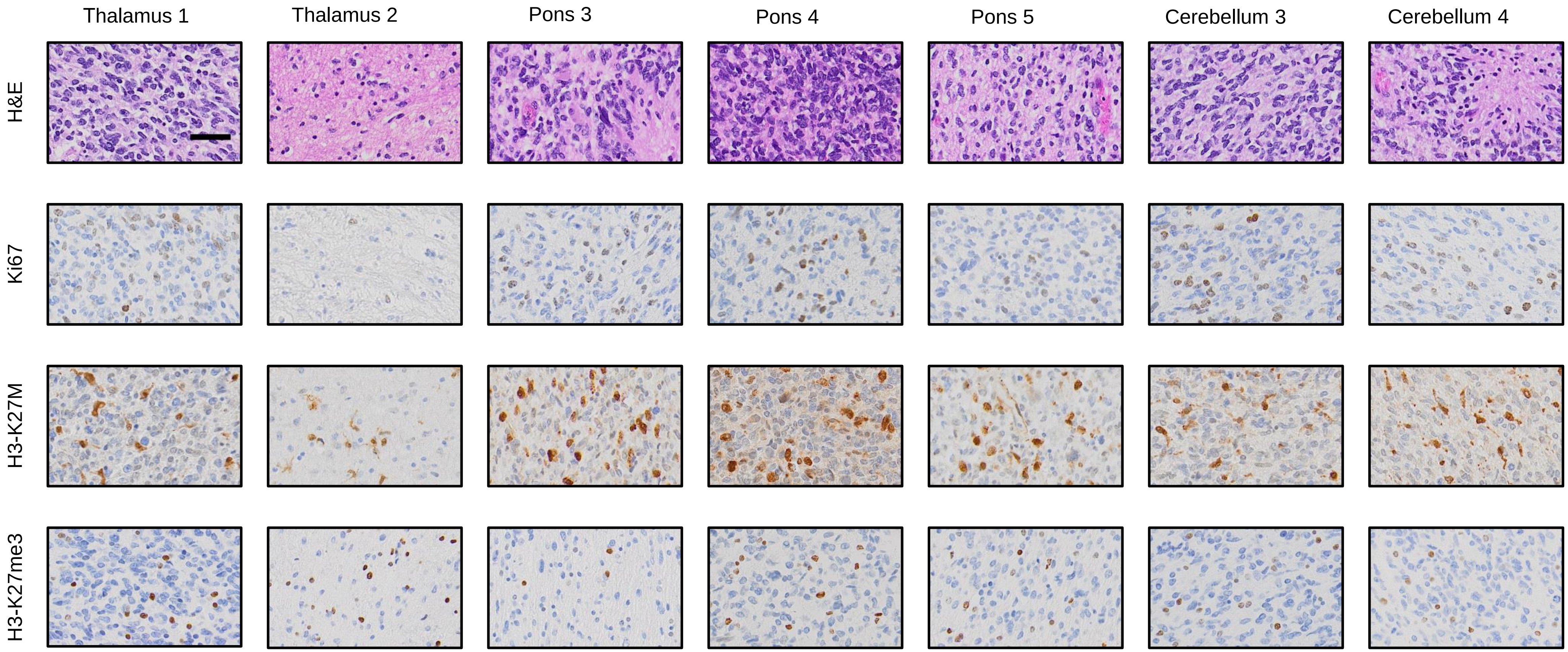

(e) DIPG9 (9y 3m; Male; H3.3 K27M)

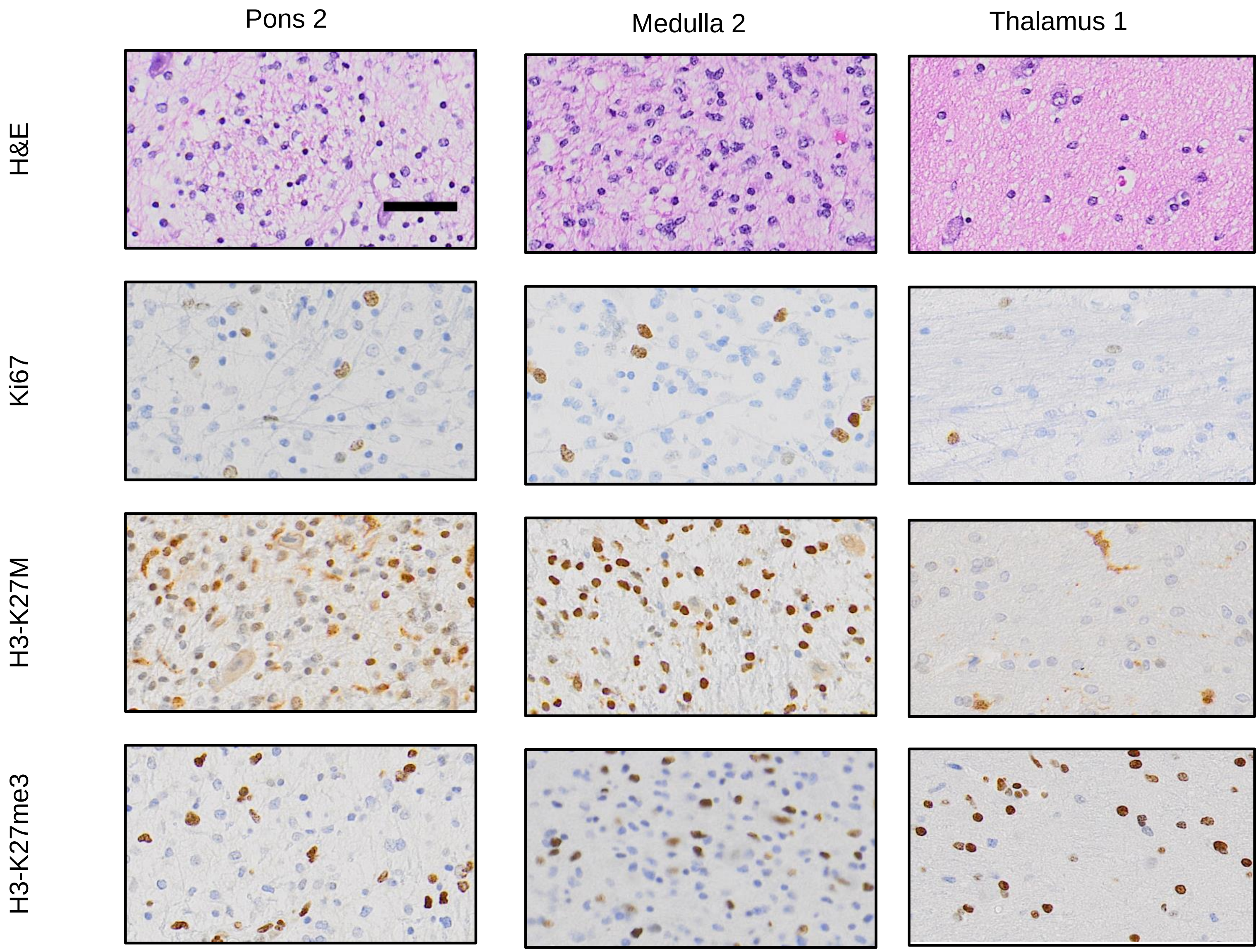

Supplementary Figure 2. Immunohistochemical staining of several neuroanatomical locations in DIPG autopsies. Histological sections were probed for Ki67 (proliferation marker), histone 3 K27M mutant (H3-K27M), and histone 3 K27 trimethylation (H3-K27me3) stains in (a) DIPG1 (b) DIPG2 (c) DIPG7 tumor (top) and normal (bottom) (d) DIPG8 and (e) DIPG9. Patient age, gender, and histone 3 mutation status are indicated above each histological staining panel.Tumor spread is seen in proximal (cerebellum and medulla) and distant (thalamus and frontal lobe) brain locations from the primary tumor in the pons. In DIPG9 (e) the area showing normal hisology had tumor infiltrate as seen using the specific anti-H3K27M staining or in our molecular studies (Supplementary Table 1). Staining performed on several areas from normal brain DIPG7 (c, bottom) shows no unspecific anti-H3K27M staining in concordance with molecular results (Supplementary Table 1). *Scale bar: 50μm.*

Supplementary Figure 3

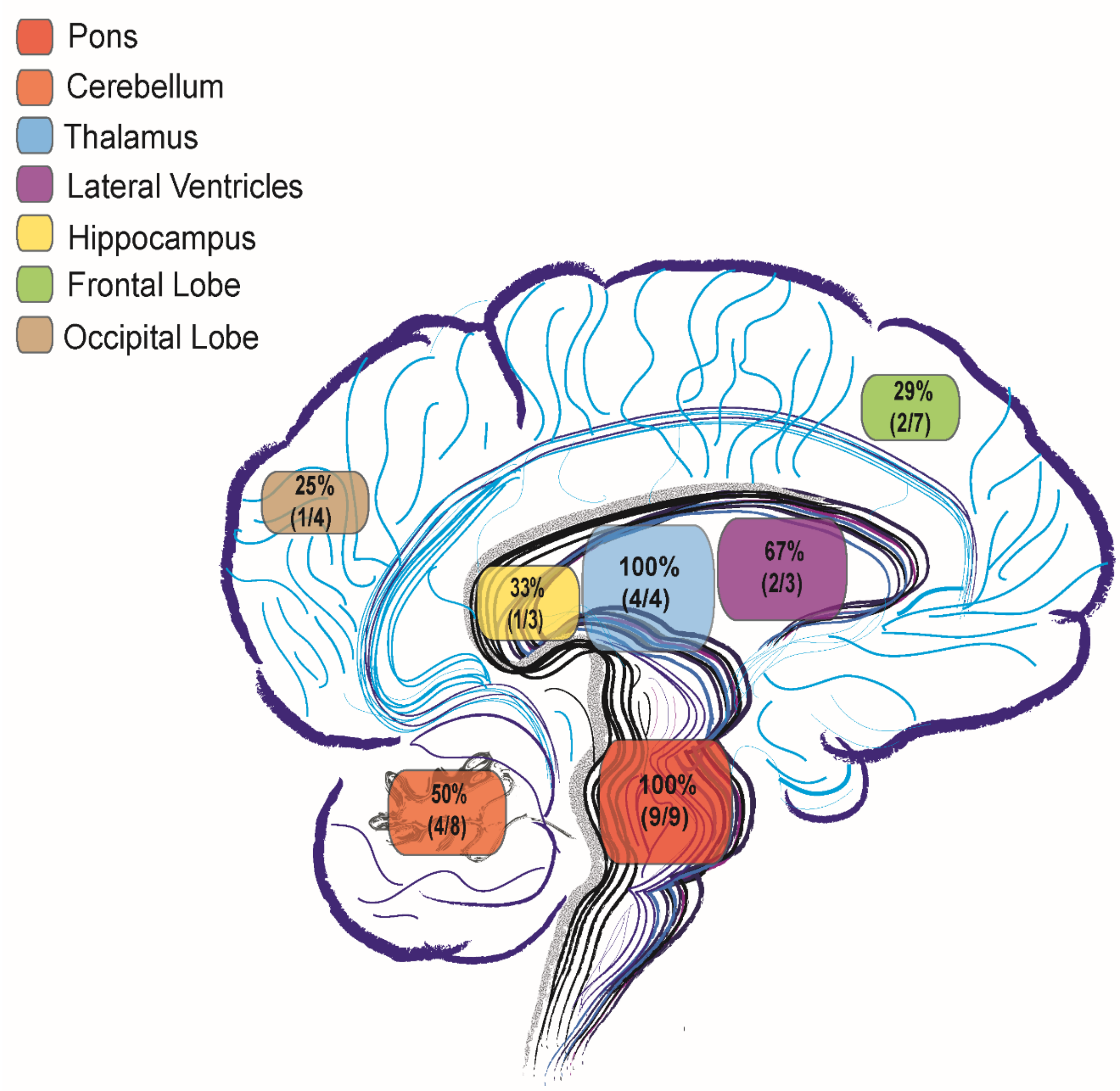

Supplementary Figure 3. Tumor extension in DIPGs. Neuroanatomical location and percent frequency of tumor extension from pons to the cerebellum, thalamus, lateral ventricles, hippocampus, frontal and occipital lobes (color key on left) in brains from nine DIPG patients. Tumor extension was detected by the presence of H3-K27M driver mutation assessed by whole exome sequencing, MiSeq targeted sequencing, and digital droplet PCR molecular analyses. Numbers in parentheses represent the percentage of tumor extension (number of patients with tumor extension/number of patients analyzed).

Supplementary Figure 4

(A)

(B)

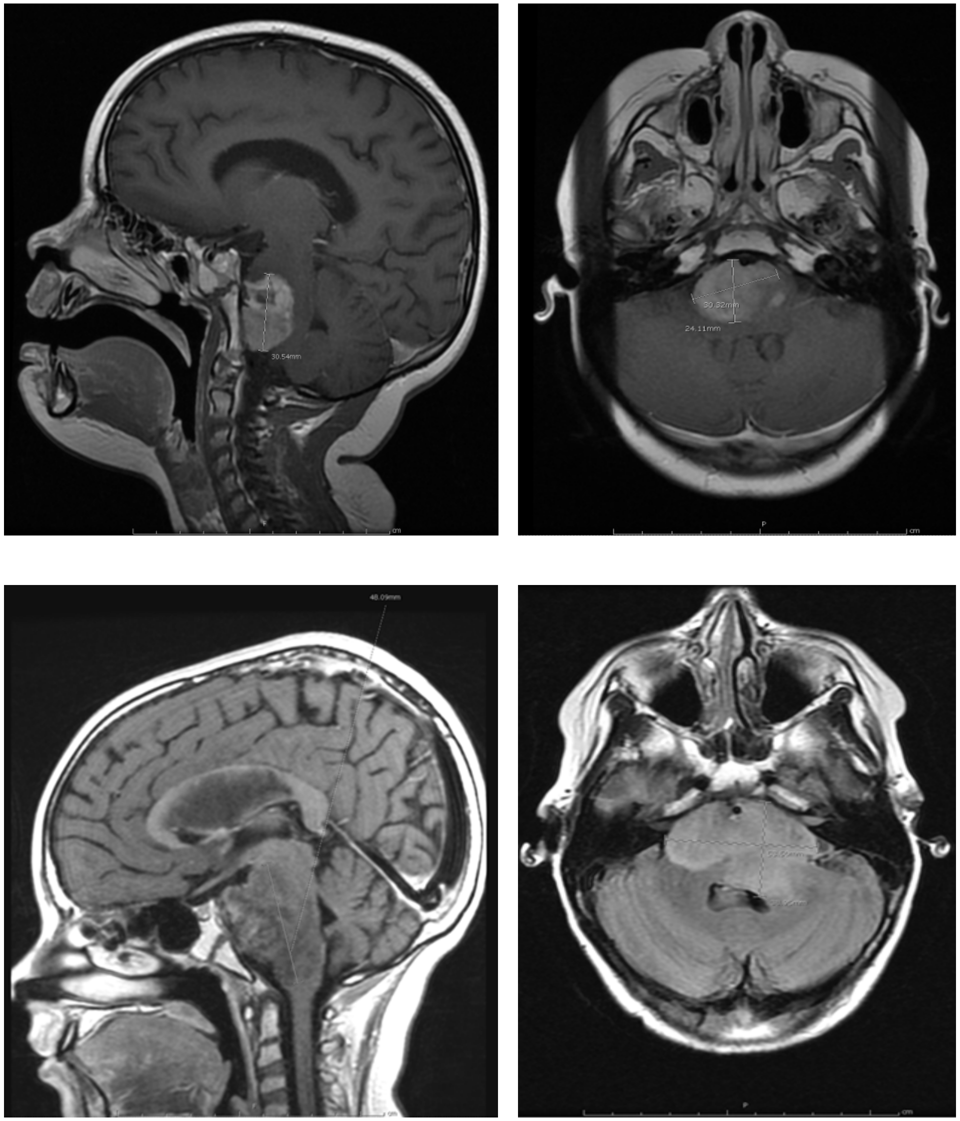

Supplementary Figure 4. Sagittal and axial view MRI of DIPG4 and DIPG7. (A) DIPG patient with non-enhancing lesion in the pons and absence of tumor extension to the cerebral cortex (DIPG4). (B) DIPG patient with non-enhancing pontine lesion and tumor extension to the lateral ventricles (DIPG7). These MRIs were taken from patients 2 weeks (DIPG7) and 3 months (DIPG4) before death, respectively.

Supplementary Figure 5

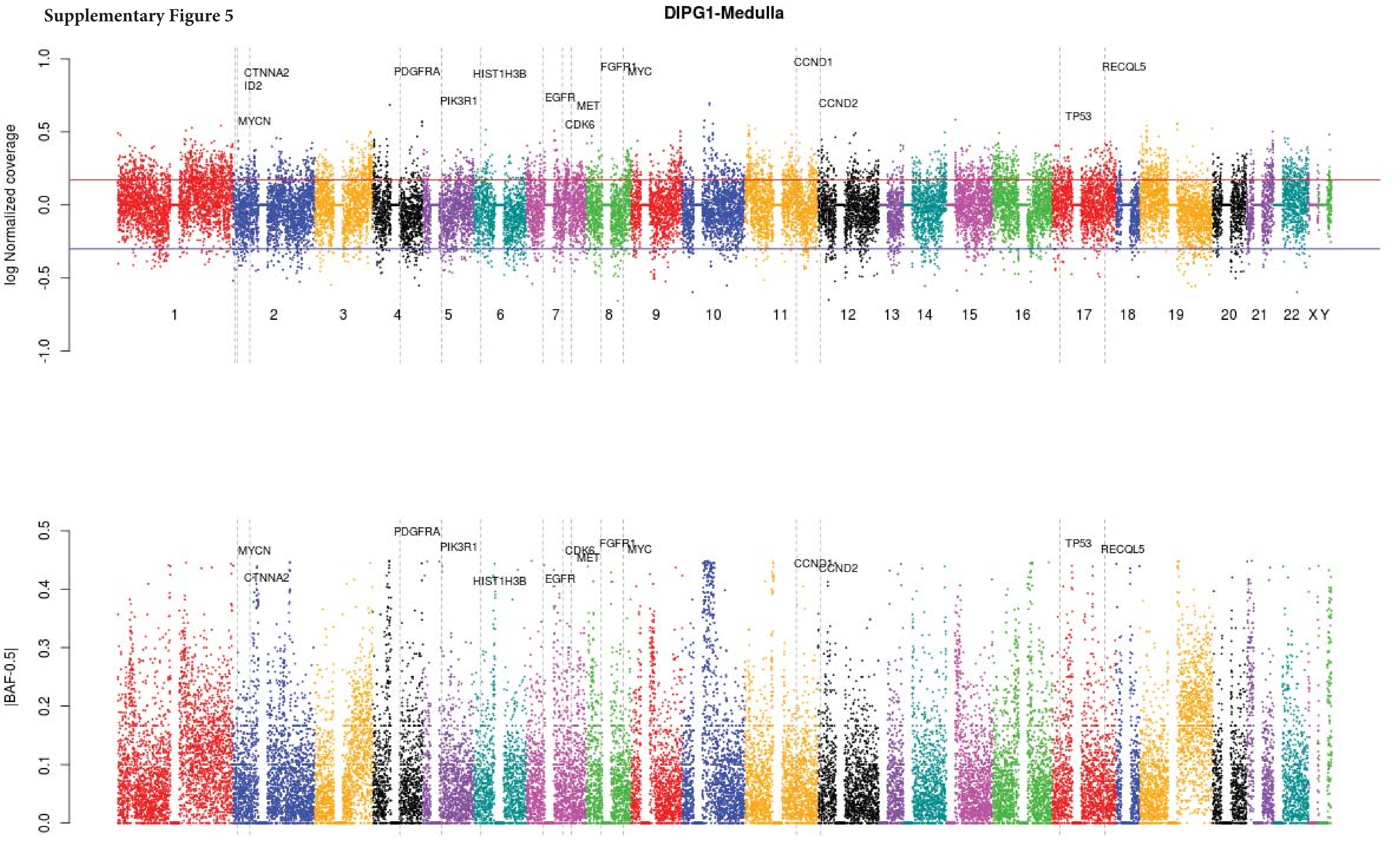

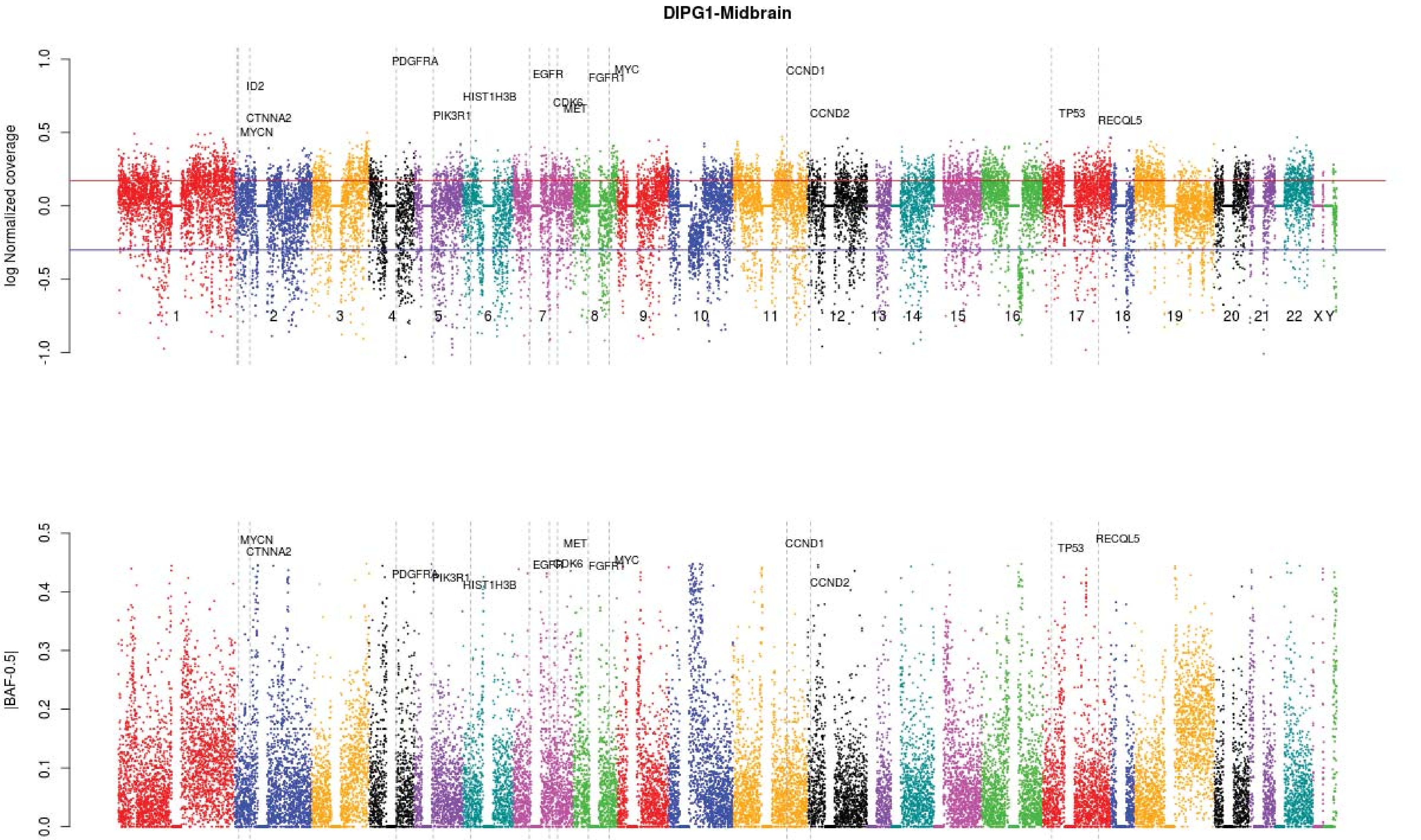

DIPG1-Midbrain
log Normalized coverage
|BAF-0.5|
ID2
CTNNA2
MYCN
PDGFRA
PIK3R1
HIST1H3B
EGFR
CDK6
MET
FGFR1
MYC
CCND1
CCND2
TP53
RECQL5
1 2 3 4 5 6 7 8 9 10 11 12 13 14 15 16 17 18 19 20 21 22 X Y

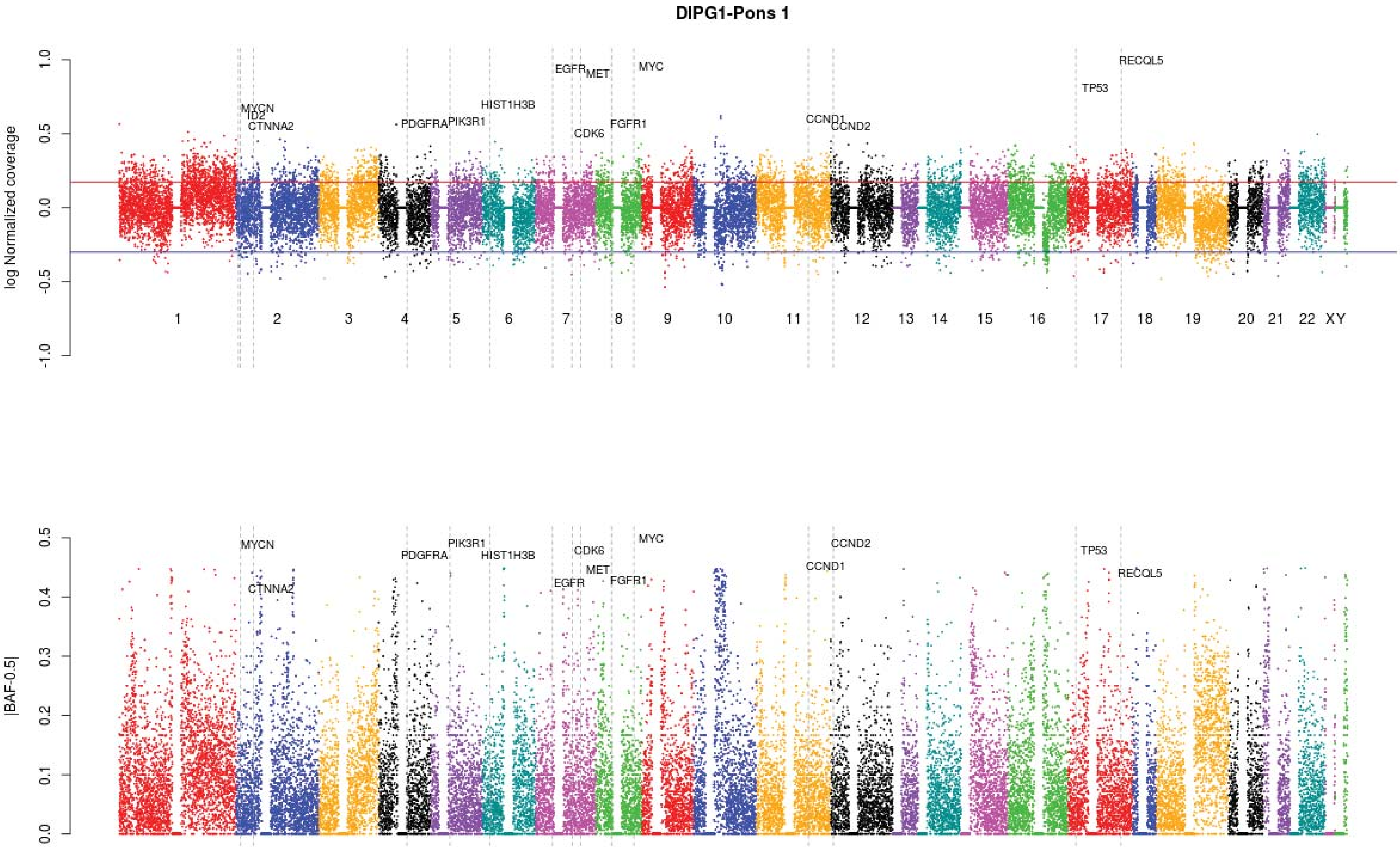

DIPG1-Pons 1
log Normalized coverage
|BAF-0.5|
MYCN
ID2
CTNNA2
PDGFRA
PIK3R1
HIST1H3B
EGFR
MET
CDK6
FGFR1
MYC
CCND1
CCND2
TP53
RECQL5
1 2 3 4 5 6 7 8 9 10 11 12 13 14 15 16 17 18 19 20 21 22 XY

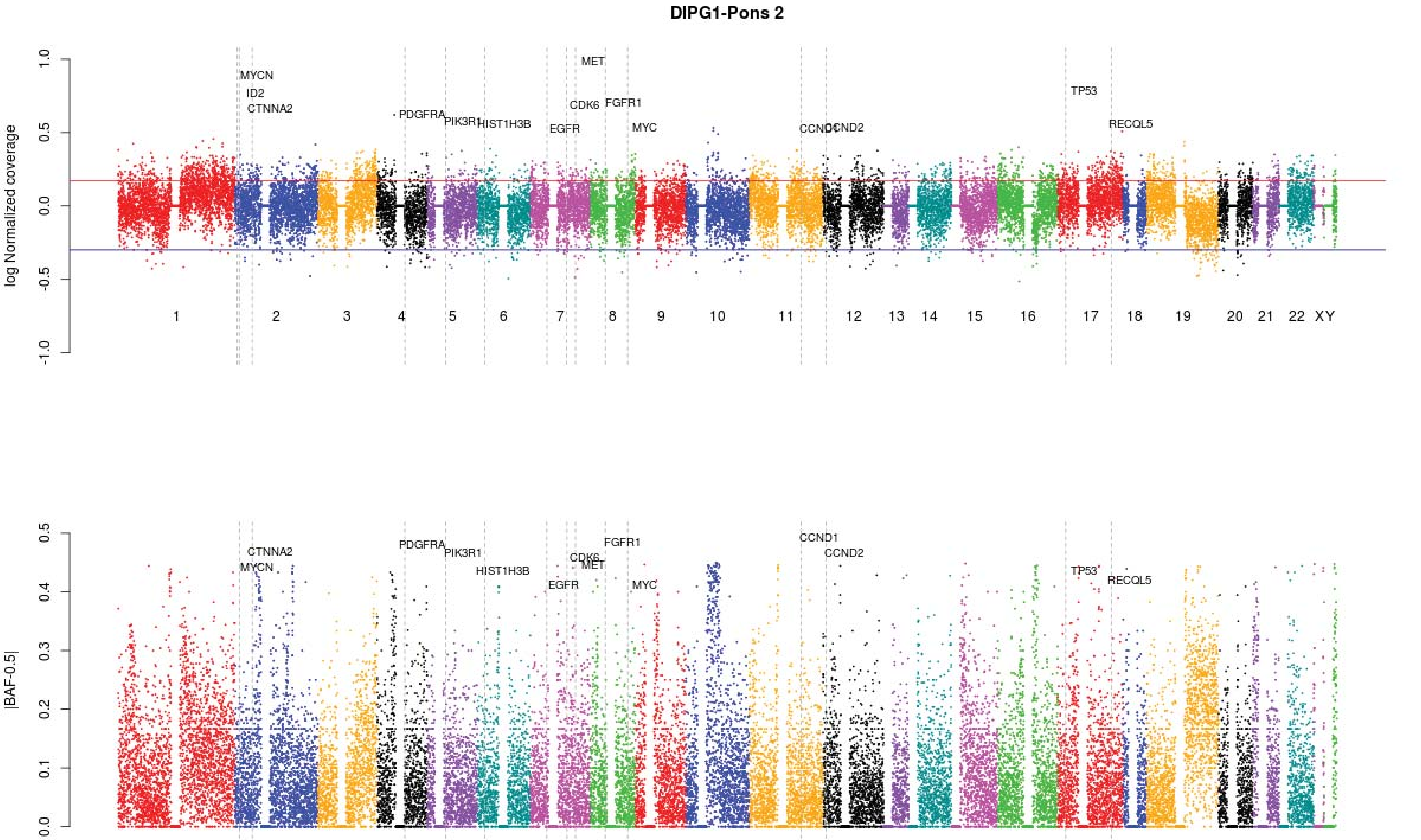

DIPG1-Pons 2
log Normalized coverage
1.0
0.5
0.0
-0.5
-1.0
MYCN
ID2
CTNNA2
PDGFRA
PIK3R1
HIST1H3B
EGFR
CDK6
MET
FGFR1
MYC
CCND1
CCND2
TP53
RECQL5
1
2
3
4
5
6
7
8
9
10
11
12
13
14
15
16
17
18
19
20
21
22
XY
|BAF-0.5|
0.5
0.4
0.3
0.2
0.1
0.0

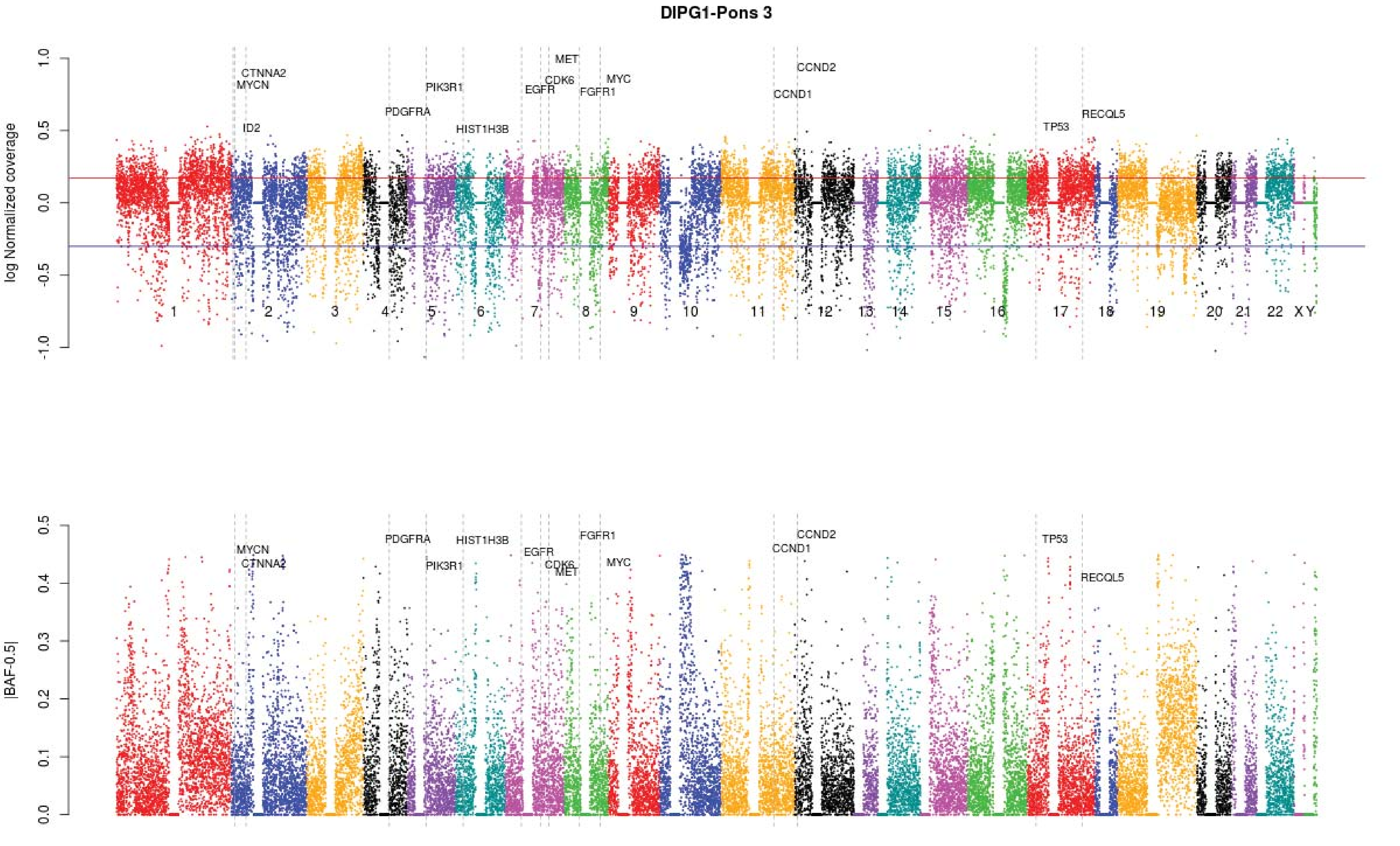

DIPG1-Pons 3
log Normalized coverage
1.0
0.5
0.0
-0.5
-1.0
CTNNA2
MYCN
ID2
PDGFRA
PIK3R1
HIST1H3B
EGFR
CDK6
MET
FGFR1
MYC
CCND2
CCND1
TP53
RECQL5
1
2
3
4
5
6
7
8
9
10
11
12
13
14
15
16
17
18
19
20
21
22
X
Y
|BAF-0.5|
0.5
0.4
0.3
0.2
0.1
0.0

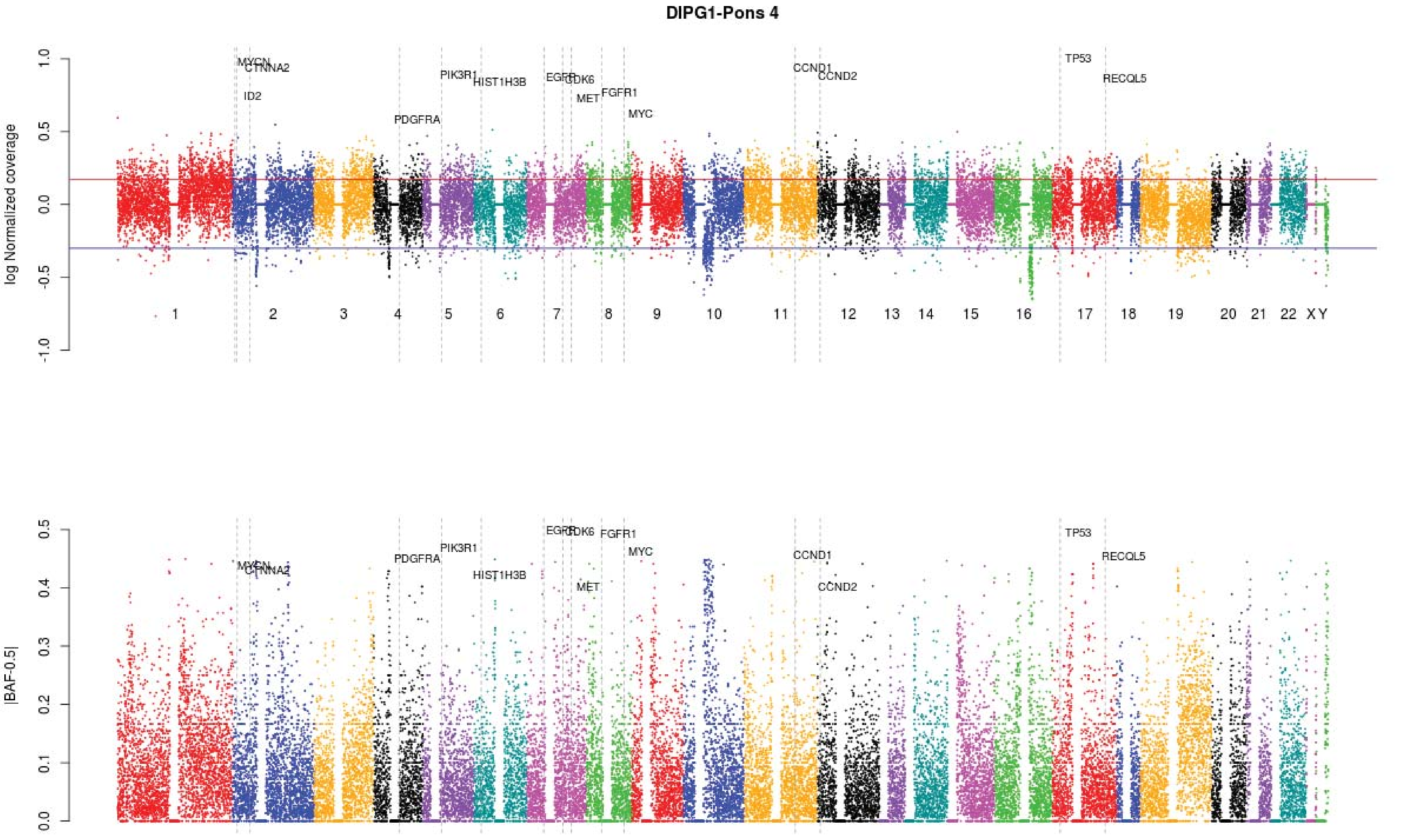

DIPG1-Pons 4
log Normalized coverage
|BAF-0.5|
MYCN
CTNNA2
ID2
PDGFRA
PIK3R1
HIST1H3B
EGFR
CDK6
MET
FGFR1
MYC
CCND1
CCND2
TP53
RECQL5
1 2 3 4 5 6 7 8 9 10 11 12 13 14 15 16 17 18 19 20 21 22 X Y

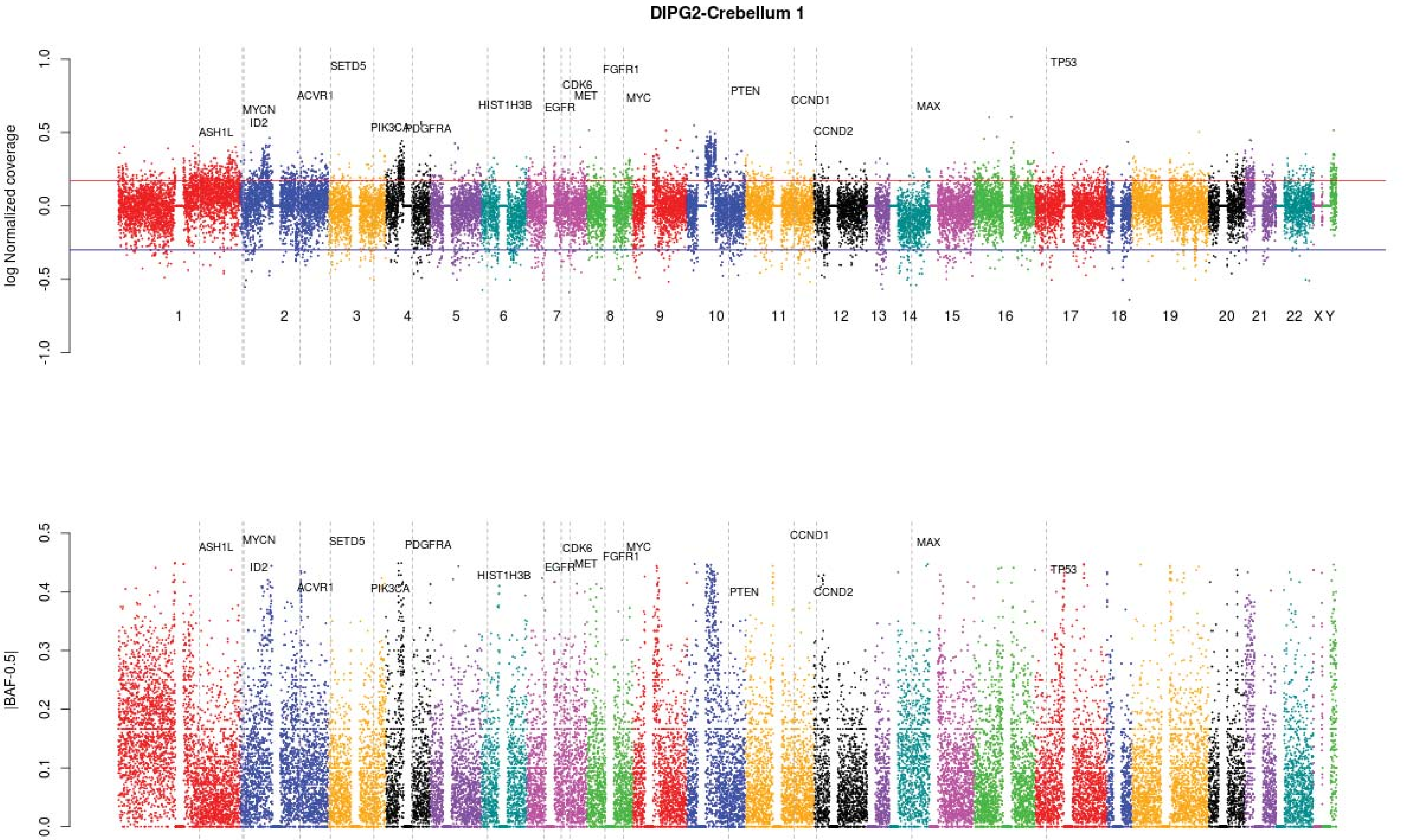
DIPG2-Crebellum 1
log Normalized coverage
1.0
0.5
0.0
-0.5
-1.0
ASH1L
MYCN
ID2
ACVR1
SETD5
PIK3CA
PDGFRA
HIST1H3B
EGFR
CDK6
MET
FGFR1
MYC
PTEN
CCND1
CCND2
MAX
TP53
1
2
3
4
5
6
7
8
9
10
11
12
13
14
15
16
17
18
19
20
21
22
X
Y
|BAF-0.5|
0.5
0.4
0.3
0.2
0.1
0.0

DIPG2-Crebellum 2

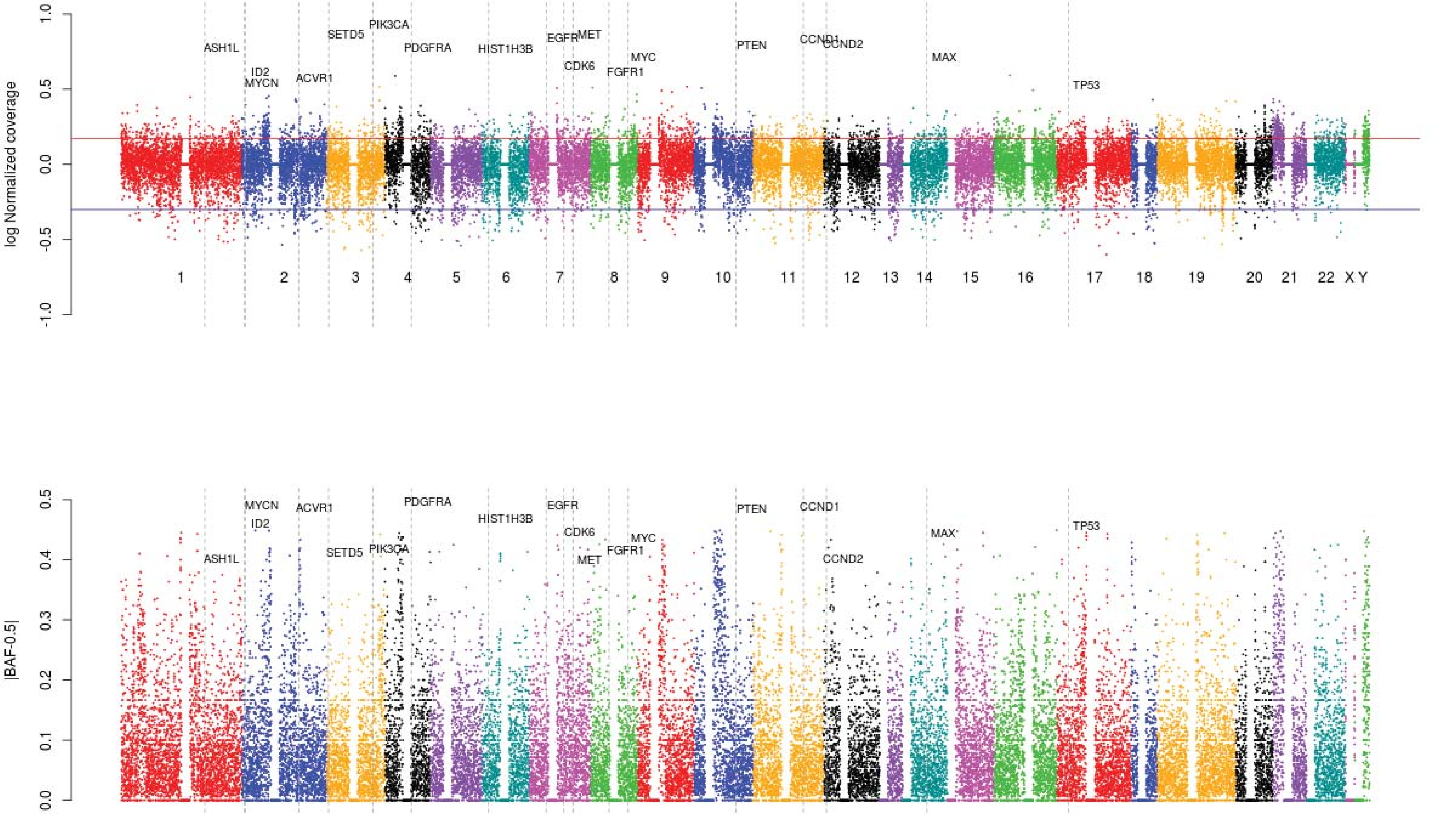

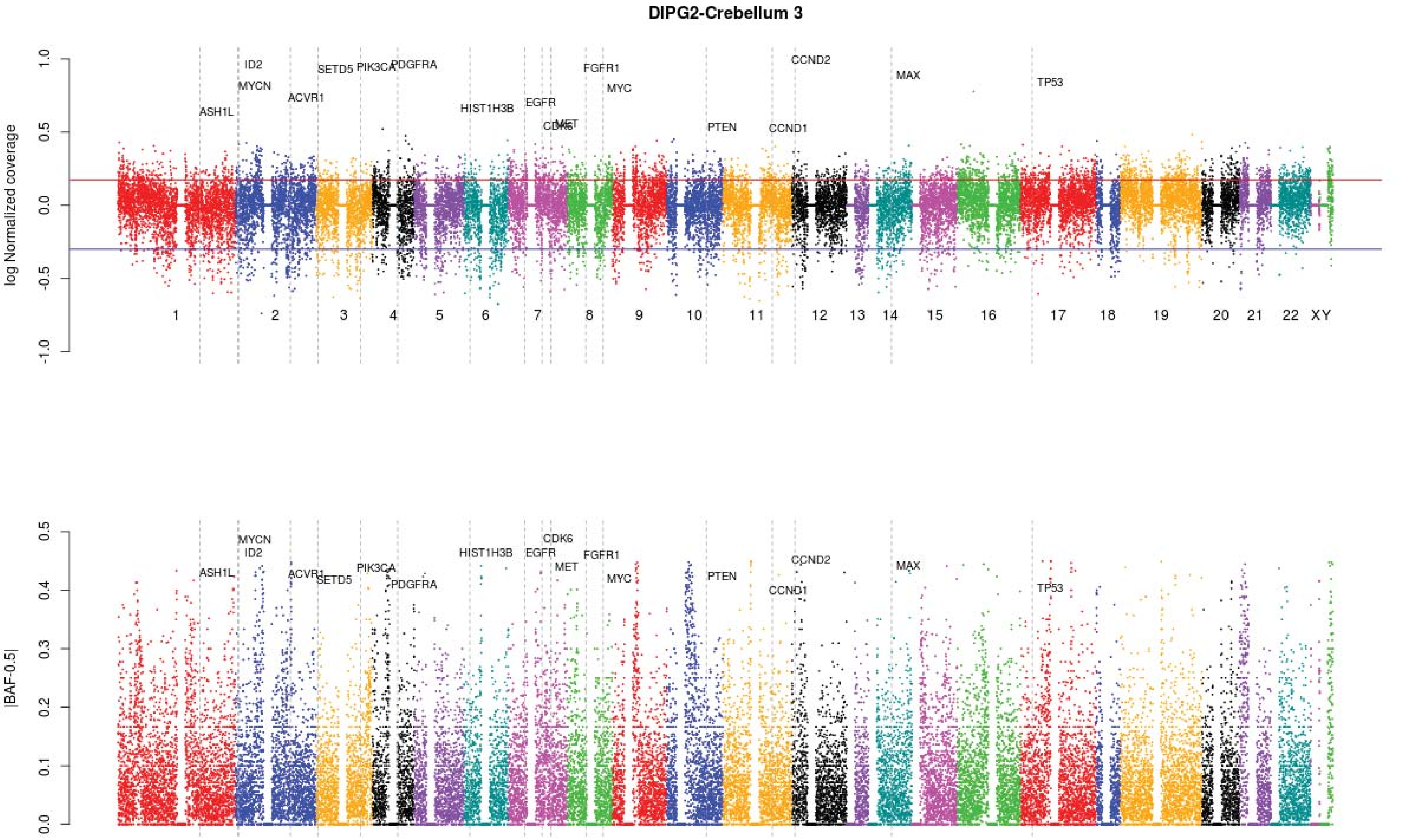
DIPG2-Crebellum 3
log Normalized coverage
1.0
0.5
0.0
-0.5
-1.0
ID2
MYCN
ASH1L
ACVR1
SETD5
PIK3CA
PDGFRA
HIST1H3B
EGFR
CDK6
MET
FGFR1
MYC
PTEN
CCND1
CCND2
MAX
TP53
1
2
3
4
5
6
7
8
9
10
11
12
13
14
15
16
17
18
19
20
21
22
XY
|BAF-0.5|
0.5
0.4
0.3
0.2
0.1
0.0
MYCN
ID2
ASH1L
ACVR1
SETD5
PIK3CA
PDGFRA
HIST1H3B
EGFR
CDK6
MET
FGFR1
MYC
PTEN
CCND1
CCND2
MAX
TP53

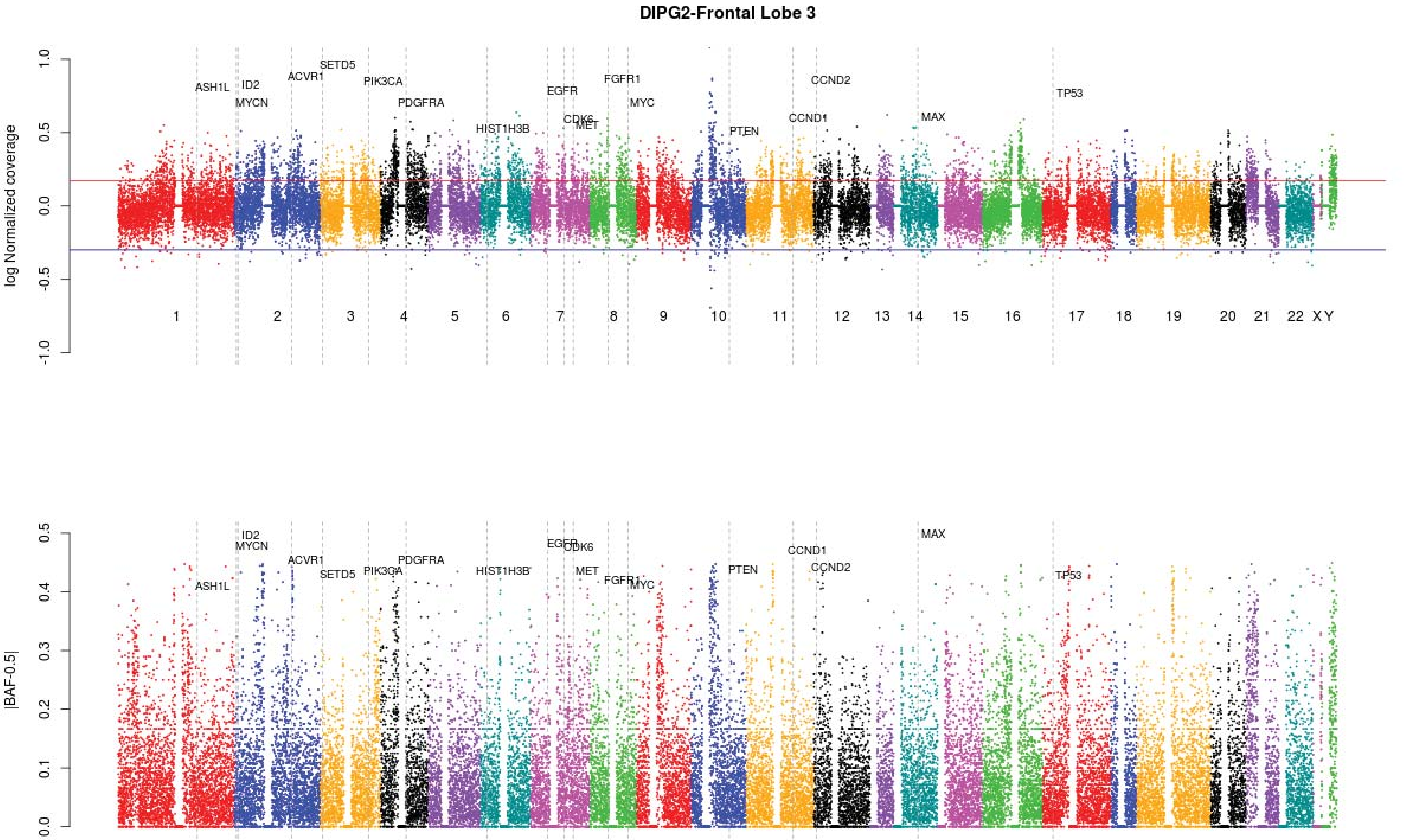
DIPG2-Frontal Lobe 3
log Normalized coverage
|BAF-0.5|
ASH1L
ID2
MYCN
ACVR1
SETD5
PIK3CA
PDGFRA
HIST1H3B
EGFR
CDK6
MET
FGFR1
MYC
PTEN
CCND1
CCND2
MAX
TP53
1 2 3 4 5 6 7 8 9 10 11 12 13 14 15 16 17 18 19 20 21 22 X Y

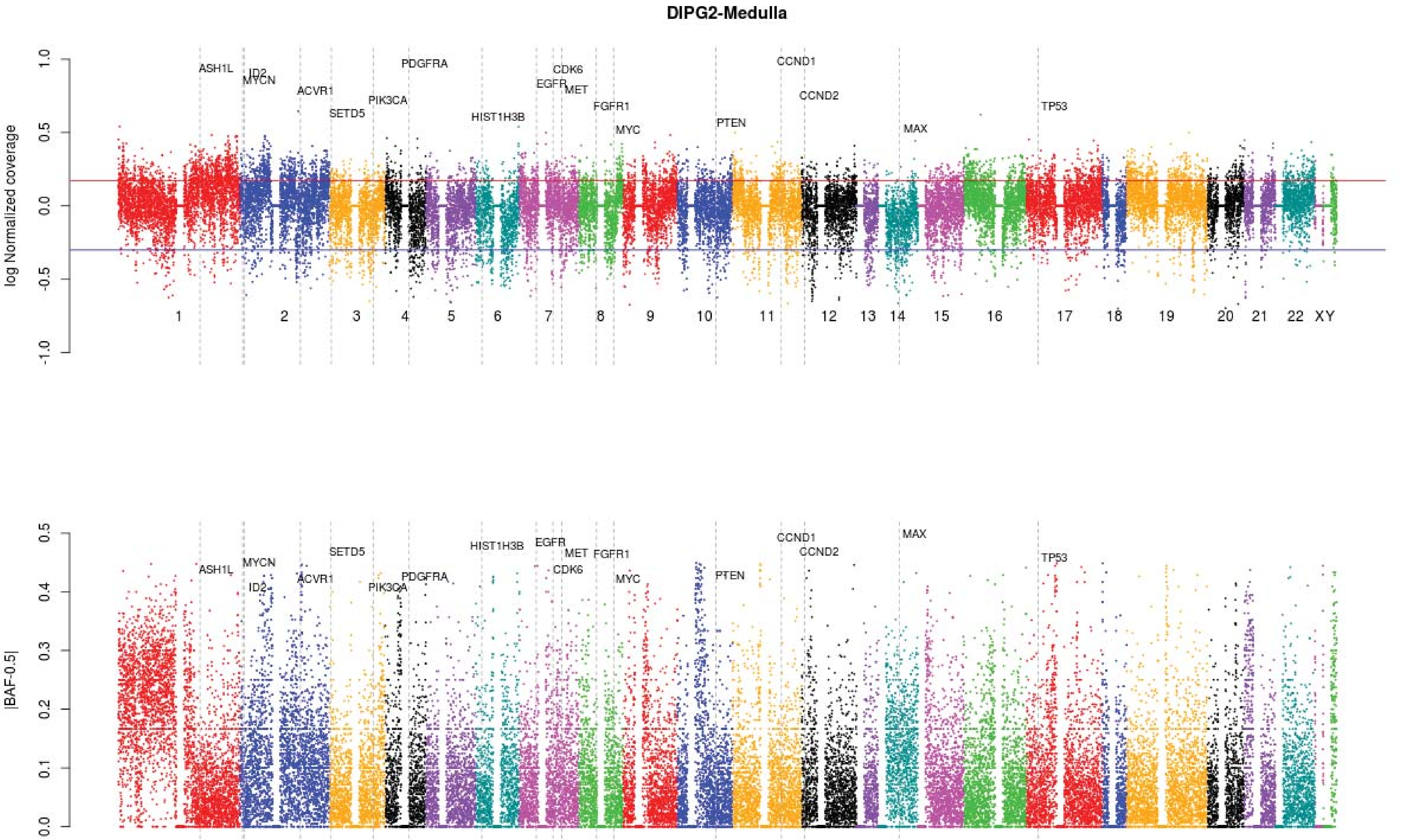

DIPG2-Medulla
log Normalized coverage
1.0
0.5
0.0
-0.5
-1.0
ASH1L
ID2
MYCN
ACVR1
SETD5
PIK3CA
PDGFRA
HIST1H3B
EGFR
CDK6
MET
FGFR1
MYC
PTEN
CCND1
CCND2
MAX
TP53
1
2
3
4
5
6
7
8
9
10
11
12
13
14
15
16
17
18
19
20
21
22
XY
|BAF-0.5|
0.5
0.4
0.3
0.2
0.1
0.0

DIPG2-Occipital Lobe 2

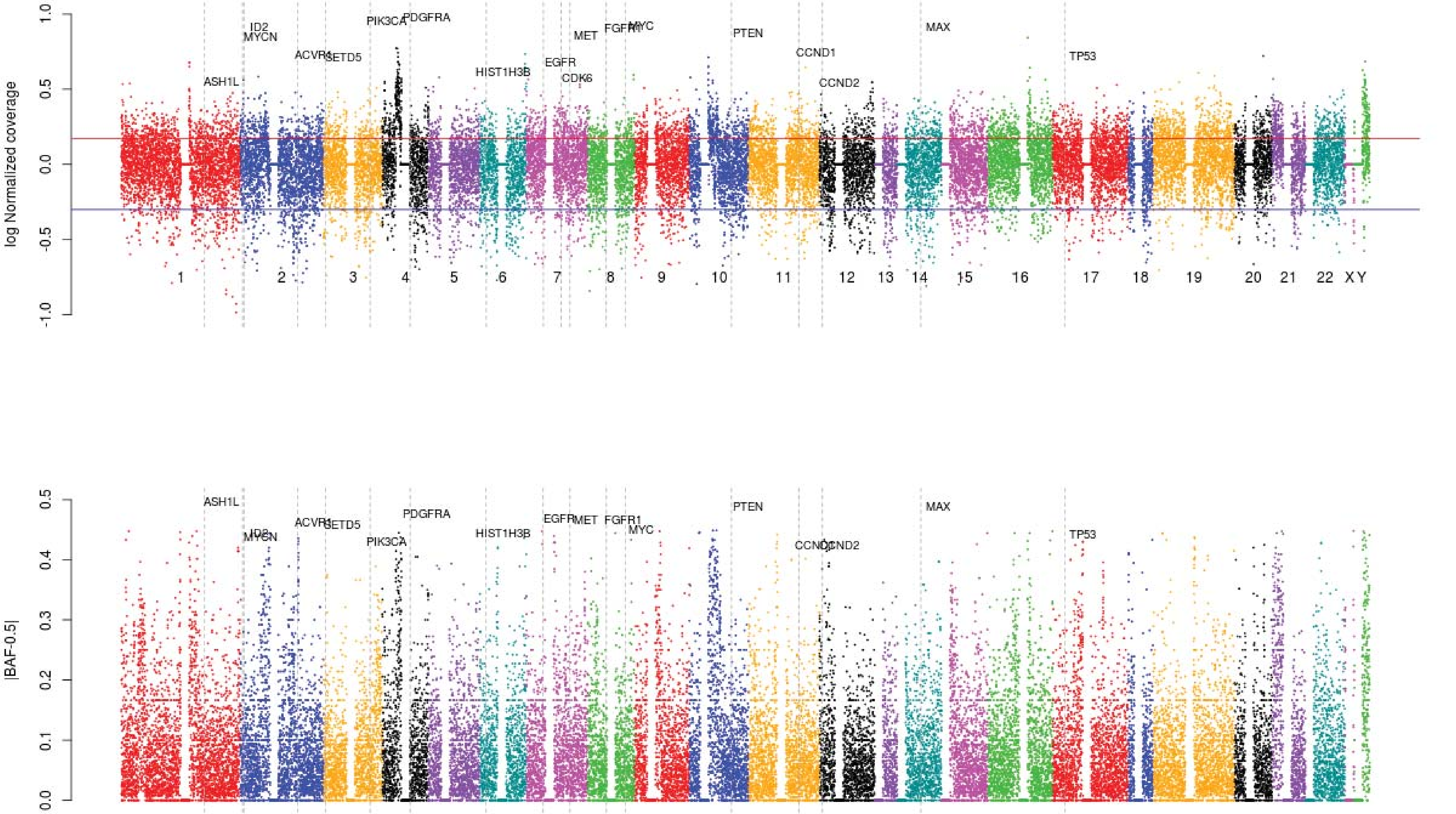

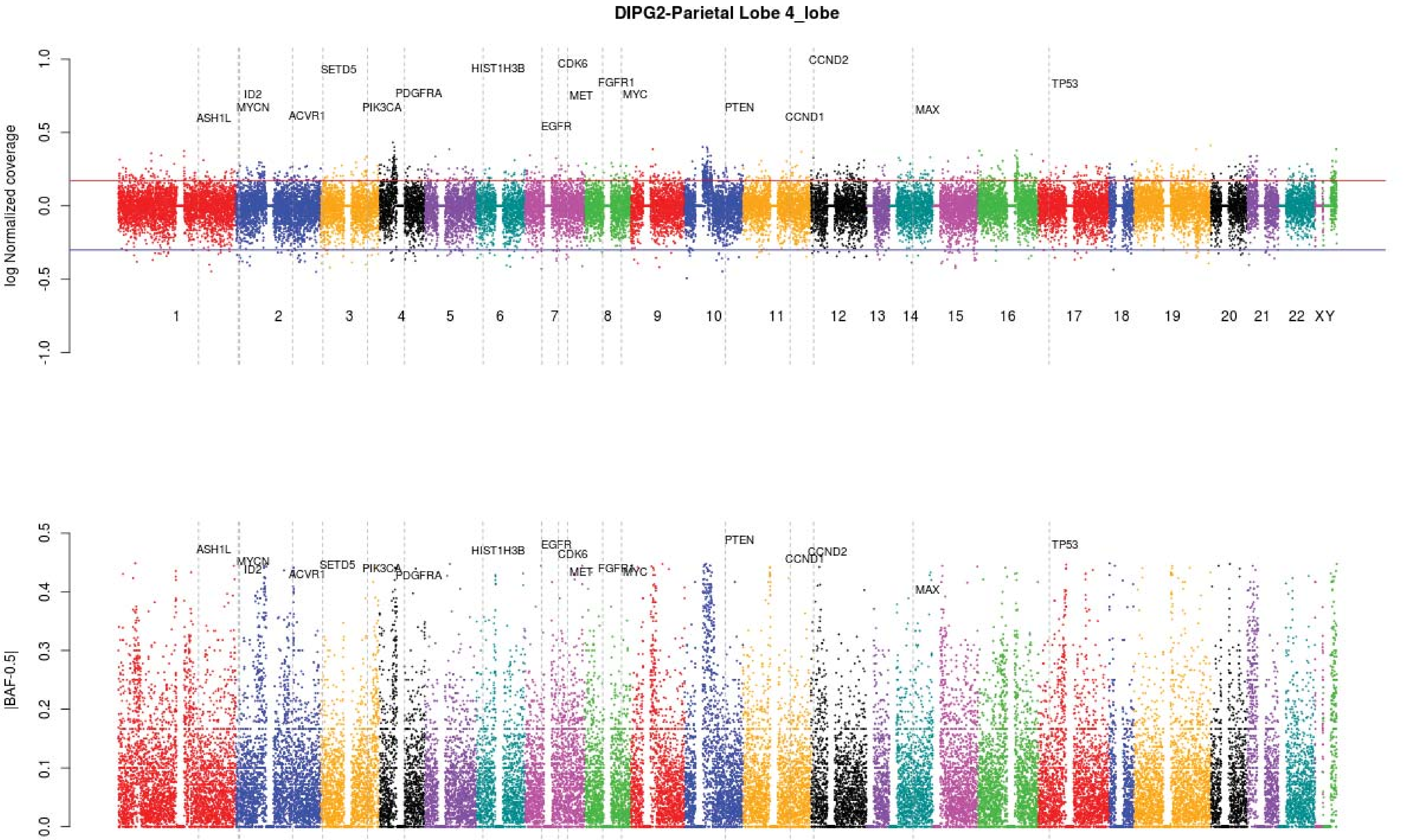
DIPG2-Parietal Lobe 4_lobe
log Normalized coverage
ASH1L
ID2
MYCN
ACVR1
SETD5
PIK3CA
PDGFRA
HIST1H3B
EGFR
CDK6
MET
FGFR1
MYC
PTEN
CCND1
CCND2
MAX
TP53
1 2 3 4 5 6 7 8 9 10 11 12 13 14 15 16 17 18 19 20 21 22 XY
|BAF-0.5|

DIPG2-Pons 1

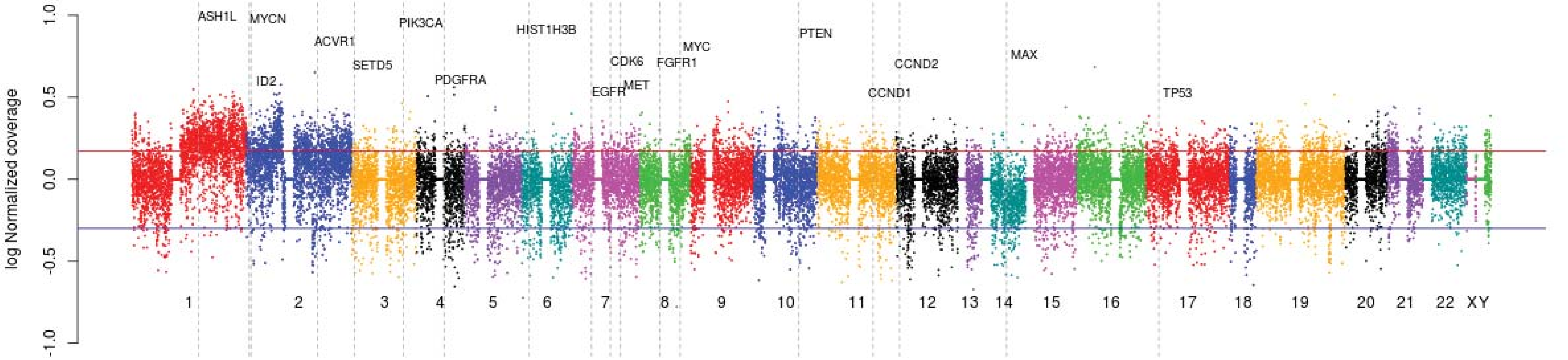

log Normalized coverage
1.0
0.5
0.0
-0.5
-1.0
ASH1L
MYCN
ID2
ACVR1
SETD5
PIK3CA
PDGFRA
HIST1H3B
EGFR
CDK6
MET
FGFR1
MYC
PTEN
CCND1
CCND2
MAX
TP53
1
2
3
4
5
6
7
8
9
10
11
12
13
14
15
16
17
18
19
20
21
22
XY

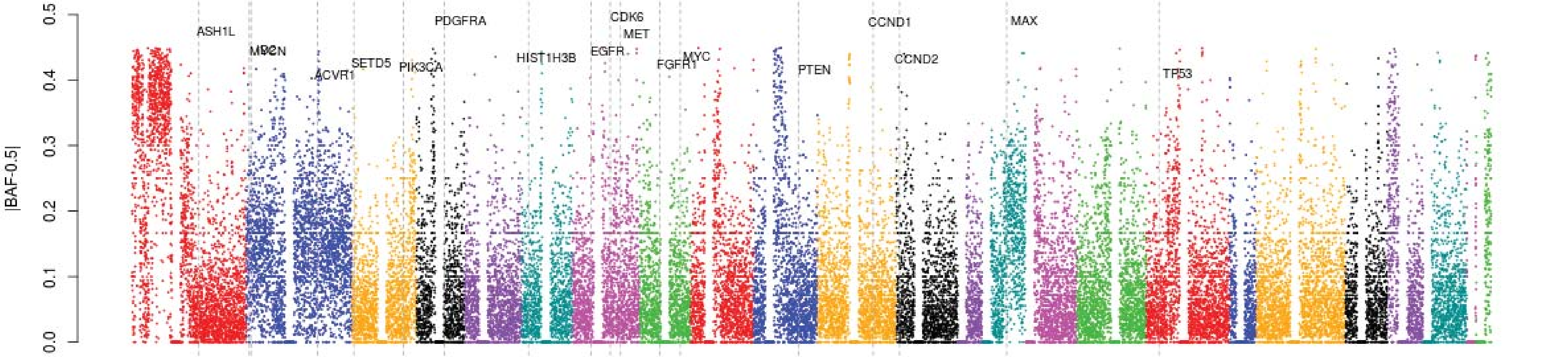

|BAF-0.5|
0.5
0.4
0.3
0.2
0.1
0.0
ASH1L
MYCN
ID2
ACVR1
SETD5
PIK3CA
PDGFRA
HIST1H3B
EGFR
CDK6
MET
FGFR1
MYC
PTEN
CCND1
CCND2
MAX
TP53

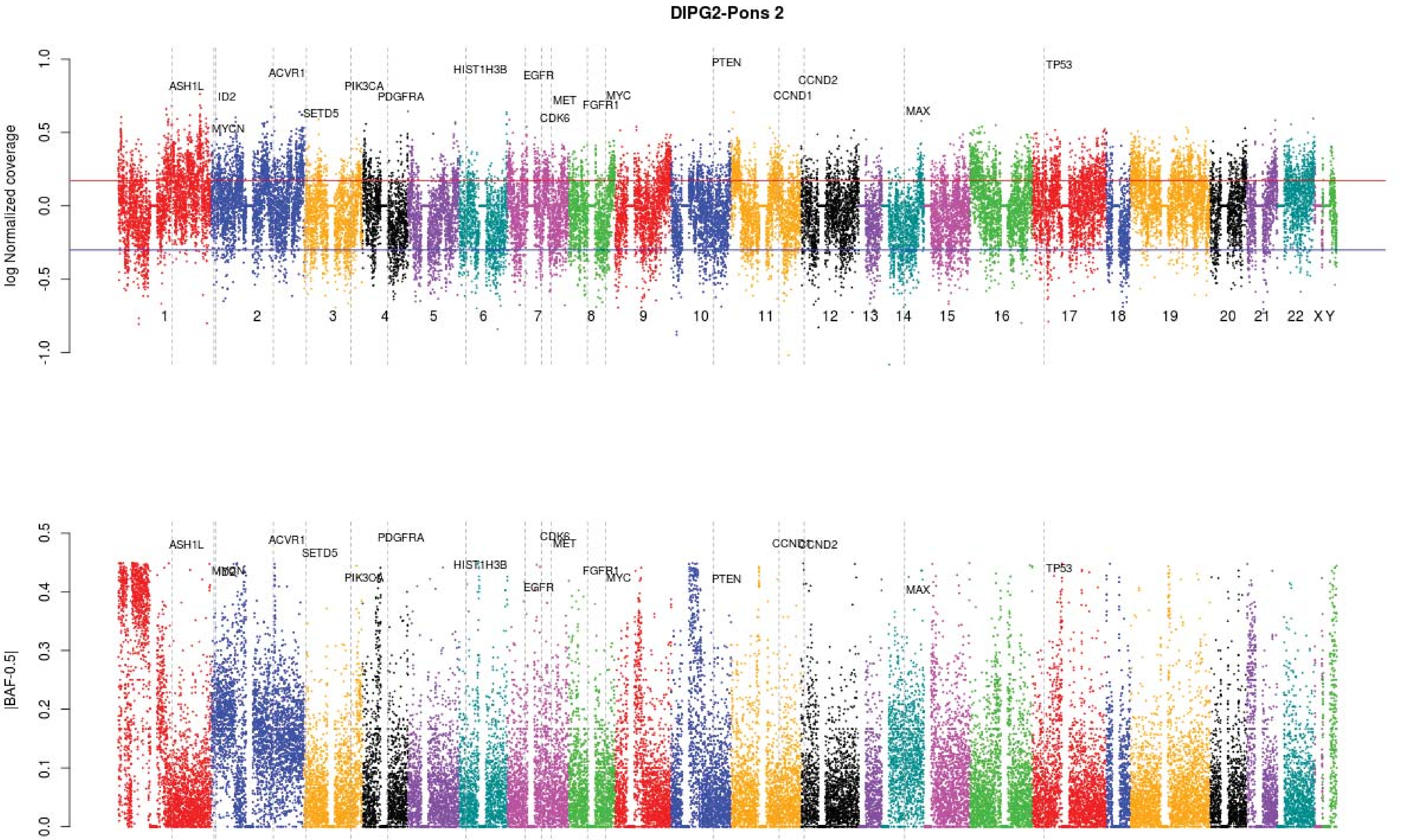

DIPG2-Pons 2
log Normalized coverage
|BAF-0.5|
ASH1L
ID2
MYCN
ACVR1
SETD5
PIK3CA
PDGFRA
HIST1H3B
EGFR
CDK6
MET
FGFR1
MYC
PTEN
CCND1
CCND2
MAX
TP53
1
2
3
4
5
6
7
8
9
10
11
12
13
14
15
16
17
18
19
20
21
22
X
Y

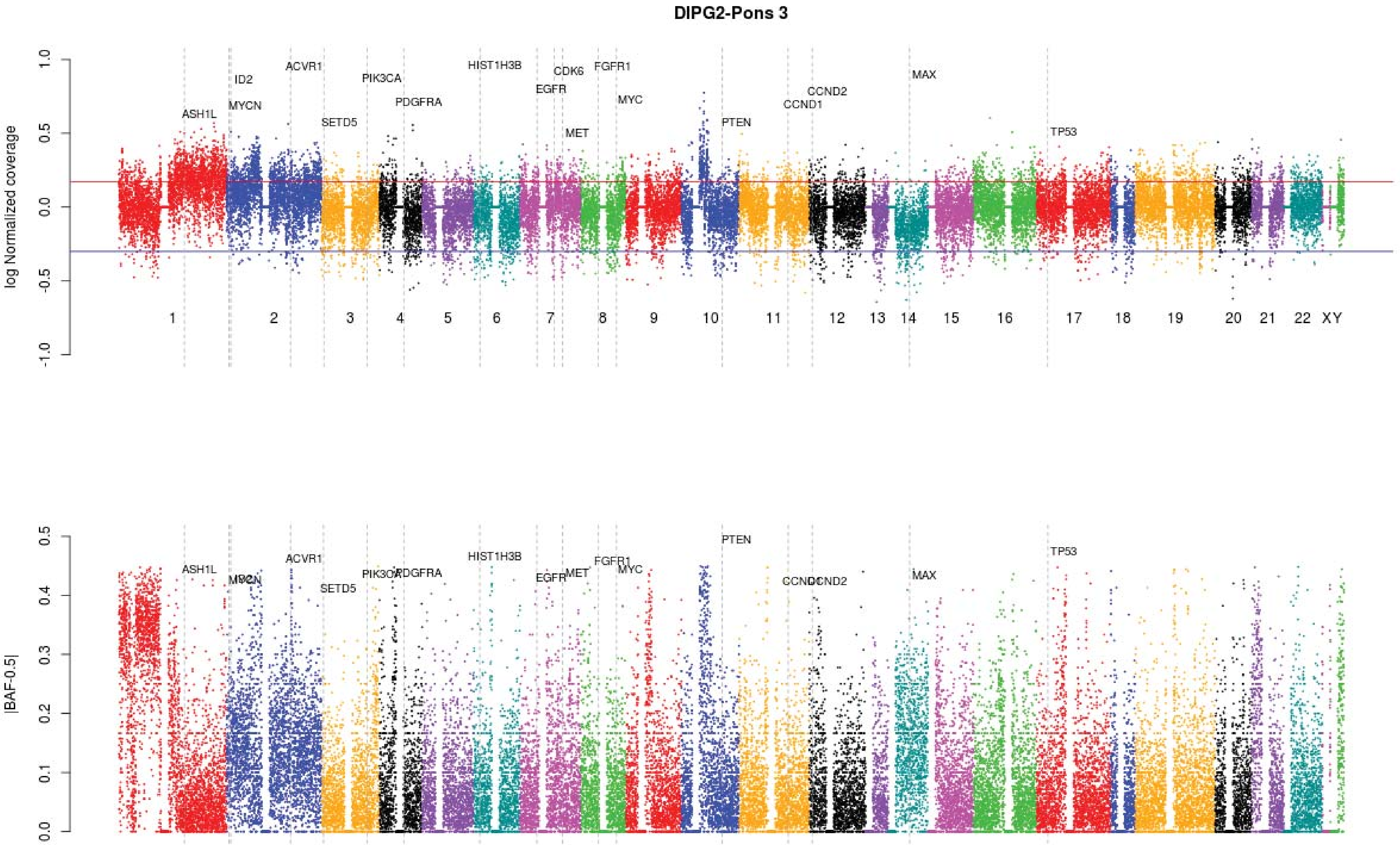
DIPG2-Pons 3
log Normalized coverage
1.0
0.5
0.0
-0.5
-1.0
ASH1L
ID2
MYCN
ACVR1
SETD5
PIK3CA
PDGFRA
HIST1H3B
EGFR
CDK6
MET
FGFR1
MYC
PTEN
CCND1
CCND2
MAX
TP53
1
2
3
4
5
6
7
8
9
10
11
12
13
14
15
16
17
18
19
20
21
22
XY
|BAF-0.5|
0.5
0.4
0.3
0.2
0.1
0.0

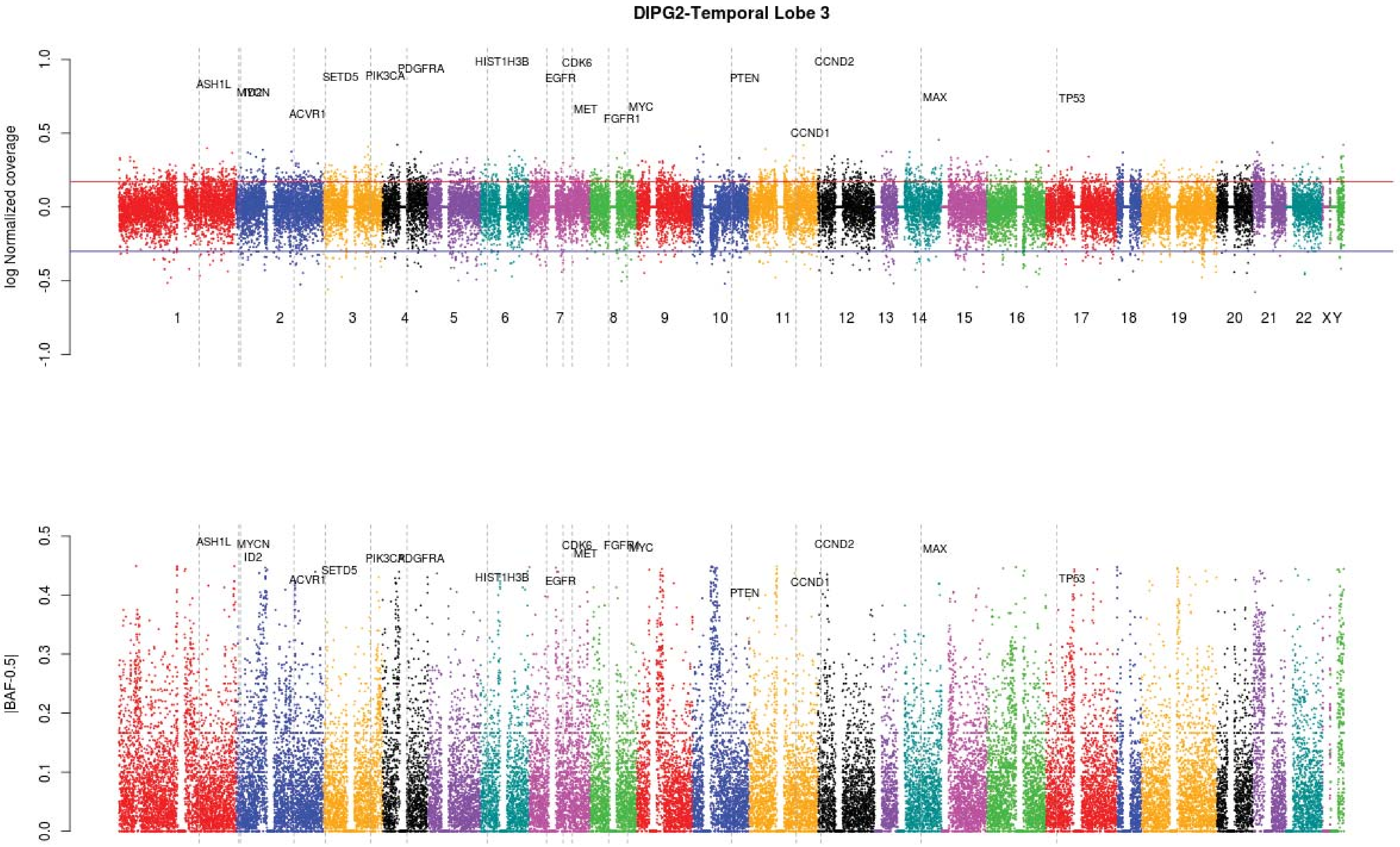
DIPG2-Temporal Lobe 3
log Normalized coverage
1.0
0.5
0.0
-0.5
-1.0
ASH1L
MYCN
ID2
ACVR1
SETD5
PIK3CA
PDGFRA
HIST1H3B
EGFR
CDK6
MET
FGFR1
MYC
PTEN
CCND1
CCND2
MAX
TP53
1
2
3
4
5
6
7
8
9
10
11
12
13
14
15
16
17
18
19
20
21
22
XY
|BAF-0.5|
0.5
0.4
0.3
0.2
0.1
0.0

DIPG2-Thalamus

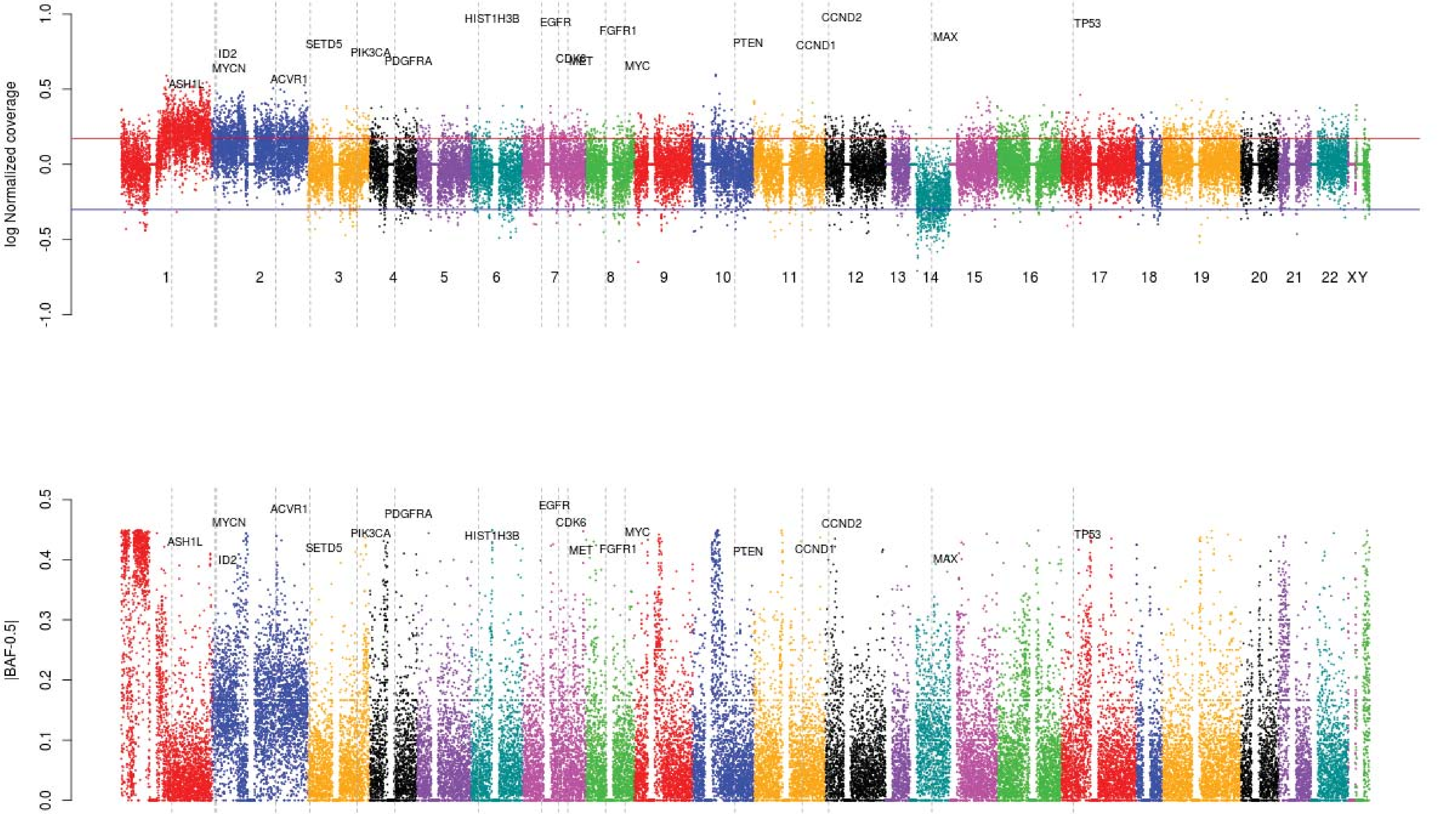

DIPG3-Pons 1

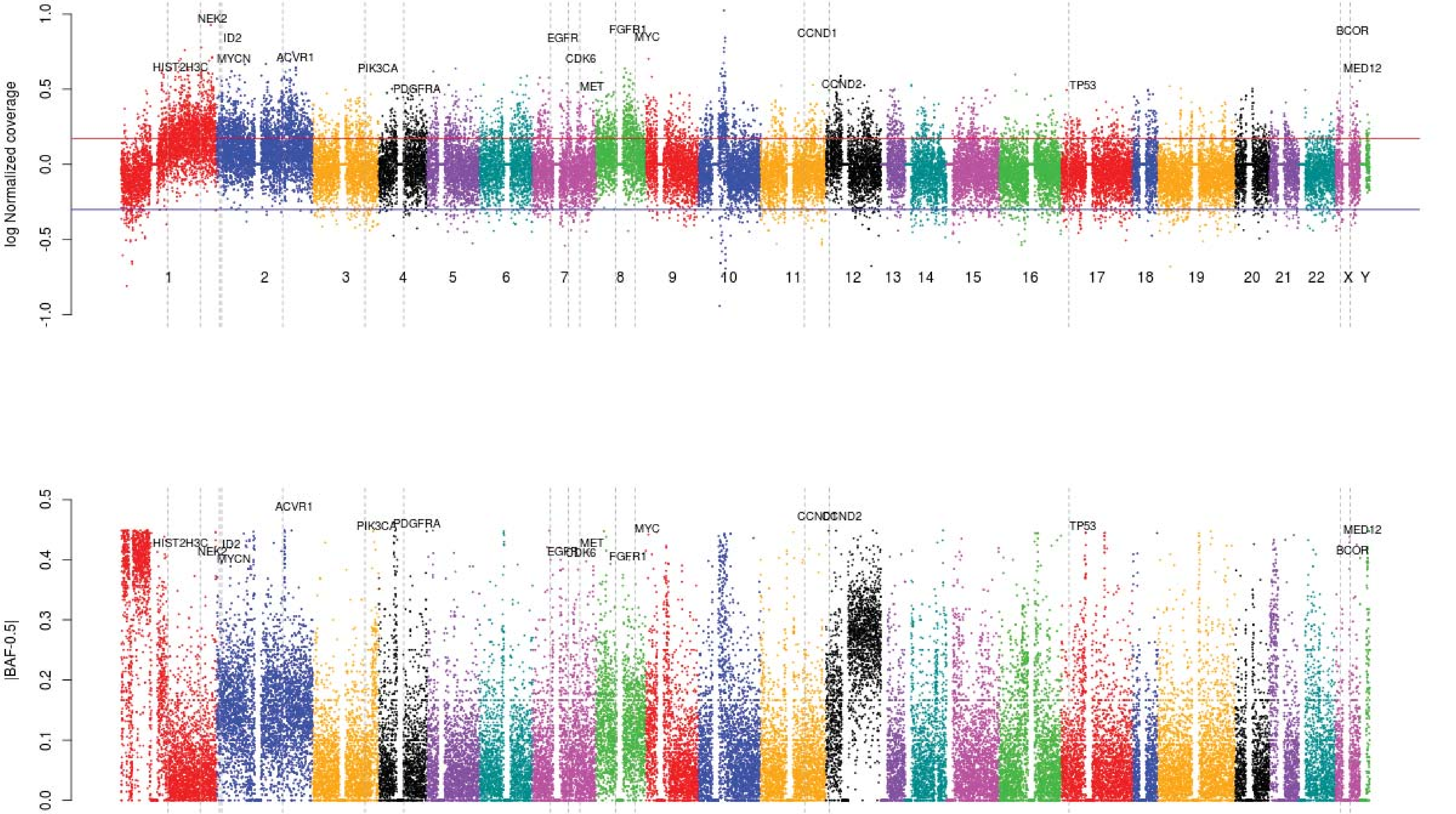
log Normalized coverage
1.0
0.5
0.0
-0.5
-1.0
NEK2
ID2
MYCN
HIST2H3C
ACVR1
PIK3CA
PDGFRA
EGFR
CDK6
MET
FGFR1
MYC
CCND1
CCND2
TP53
BCOR
MED12
1
2
3
4
5
6
7
8
9
10
11
12
13
14
15
16
17
18
19
20
21
22
X
Y
|BAF-0.5|
0.5
0.4
0.3
0.2
0.1
0.0

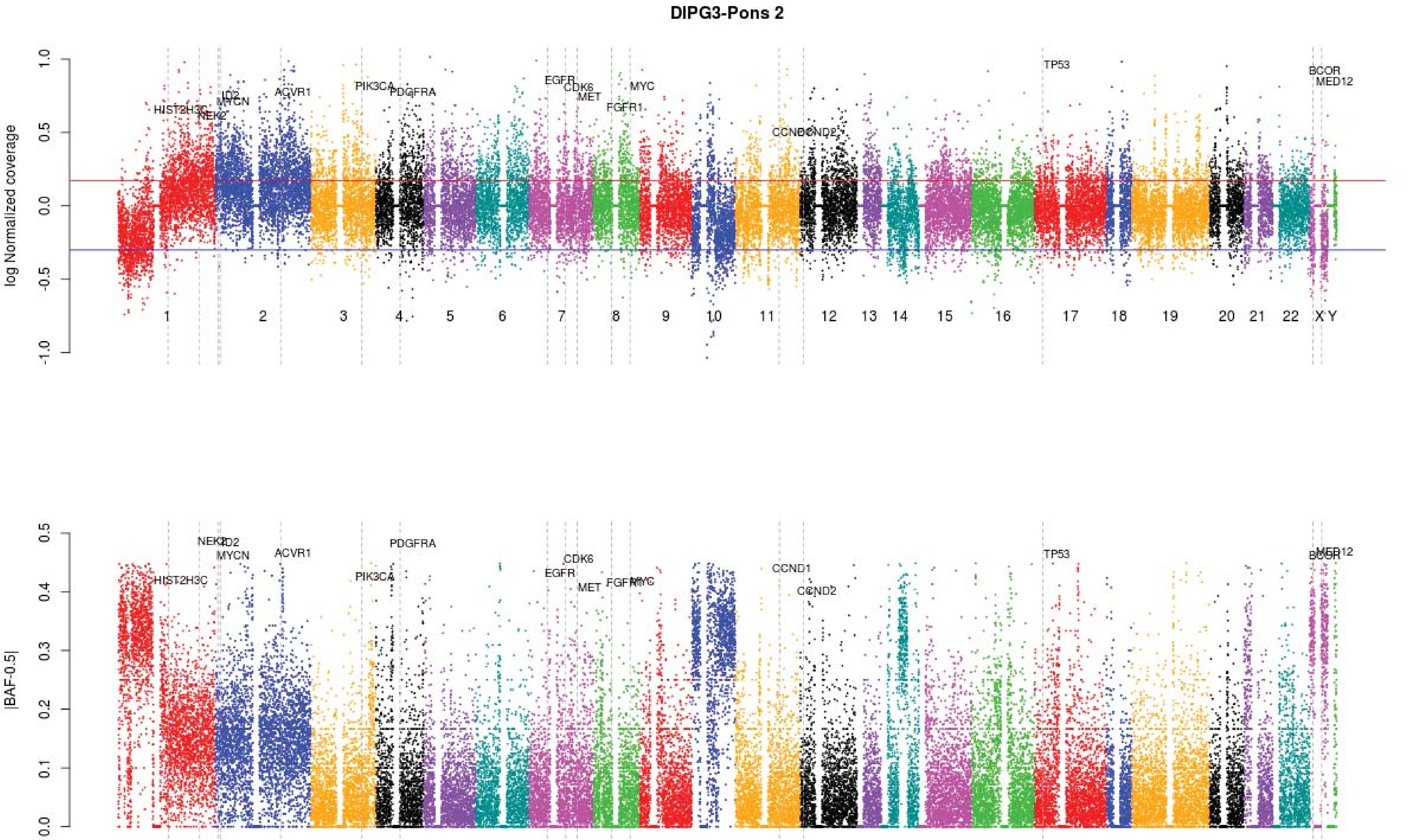

DIPG3-Pons 2
log Normalized coverage
|BAF-0.5|
HIST2H3C
NEK2
ID2
MYCN
ACVR1
PIK3CA
PDGFRA
EGFR
CDK6
MET
FGFR1
MYC
CCND1
CCND2
TP53
BCOR
MED12
1
2
3
4
5
6
7
8
9
10
11
12
13
14
15
16
17
18
19
20
21
22
X
Y

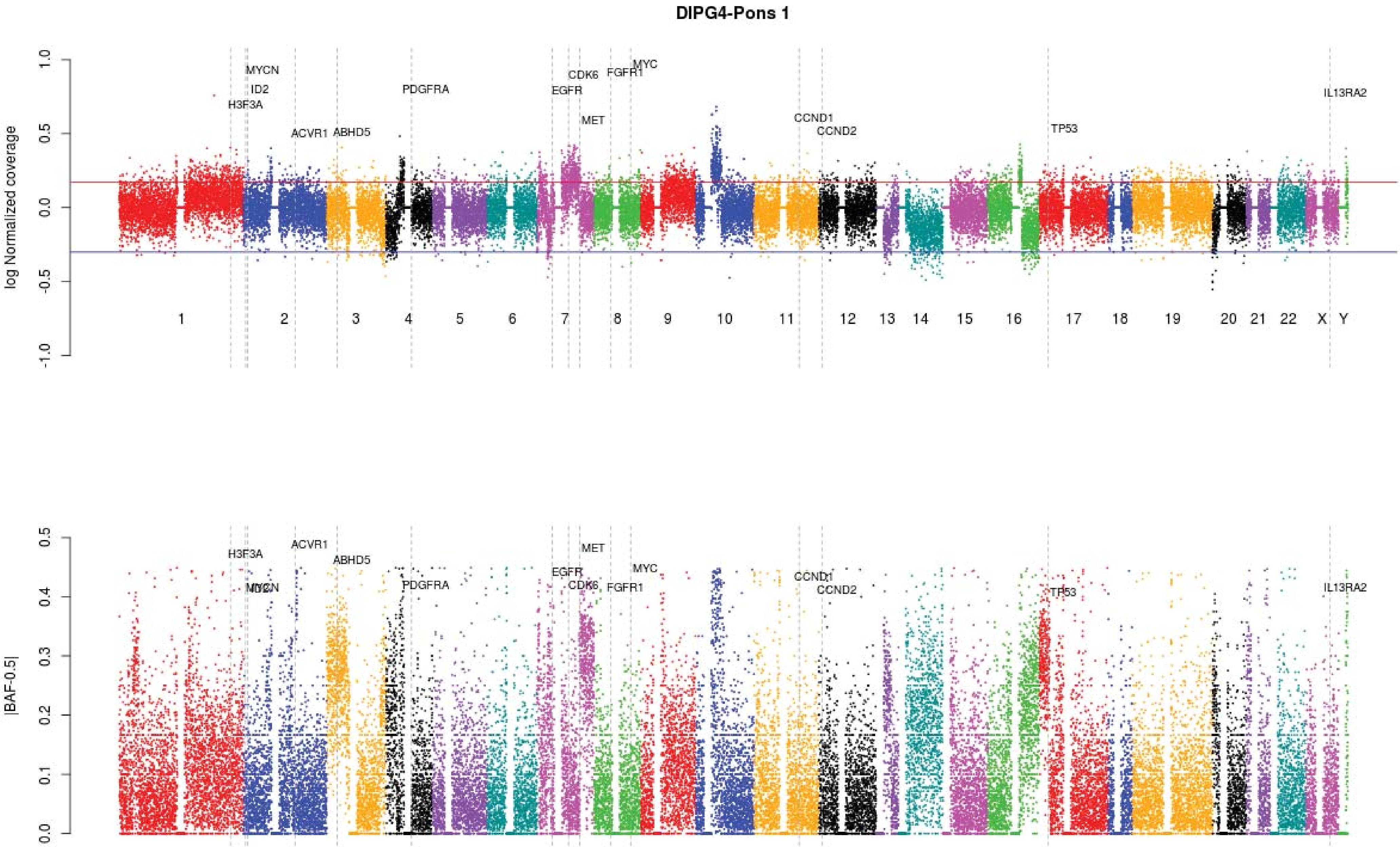
DIPG4-Pons 1
log Normalized coverage
|BAF-0.5|
MYCN
ID2
H3F3A
ACVR1
ABHD5
PDGFRA
EGFR
CDK6
MET
FGFR1
MYC
CCND1
CCND2
TP53
IL13RA2
1 2 3 4 5 6 7 8 9 10 11 12 13 14 15 16 17 18 19 20 21 22 X Y

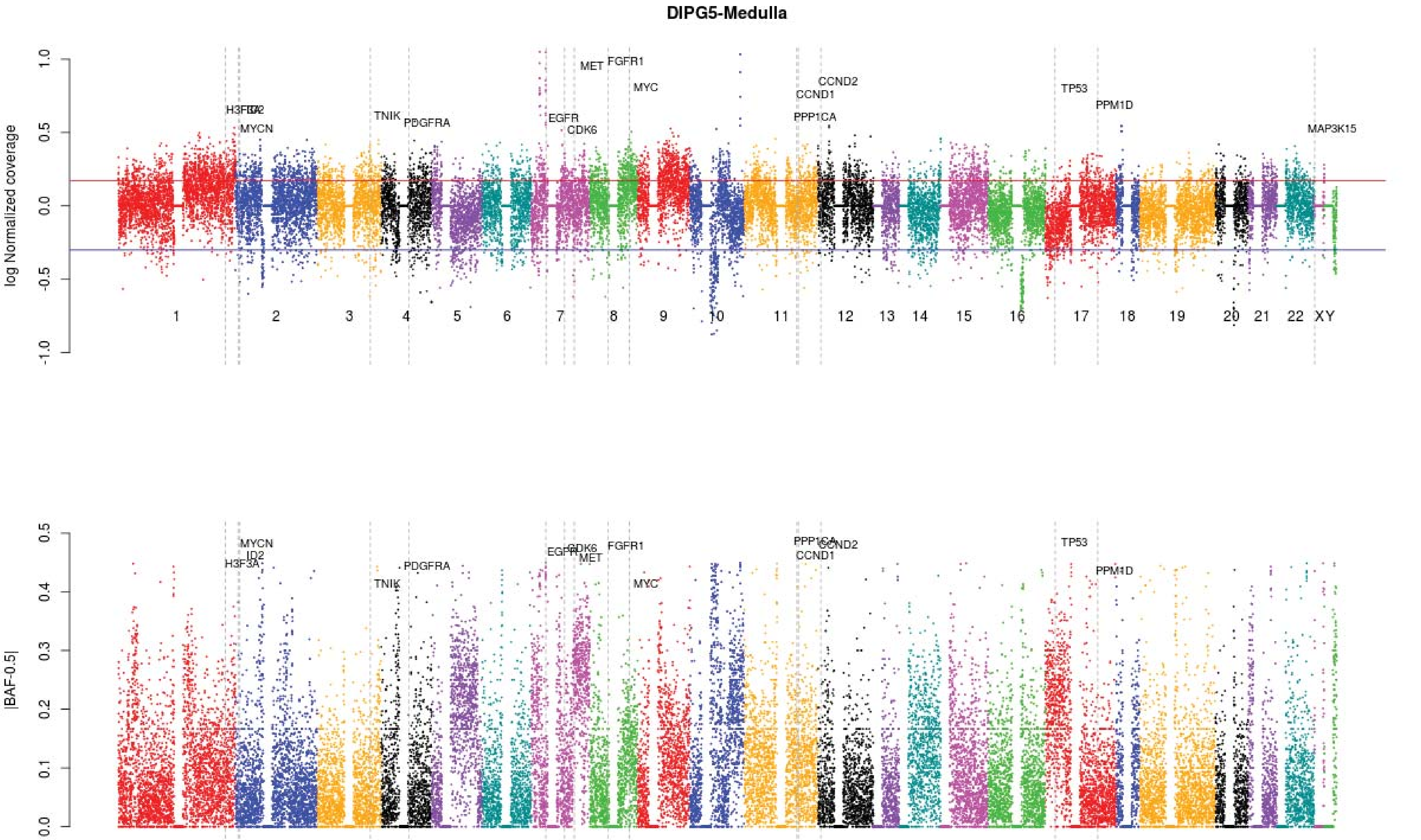

DIPG5-Medulla
log Normalized coverage
|BAF-0.5|
H3F3A
ID2
MYCN
TNIK
PDGFRA
EGFR
CDK6
MET
FGFR1
MYC
CCND2
CCND1
PPP1CA
TP53
PPM1D
MAP3K15
1
2
3
4
5
6
7
8
9
10
11
12
13
14
15
16
17
18
19
20
21
22
XY

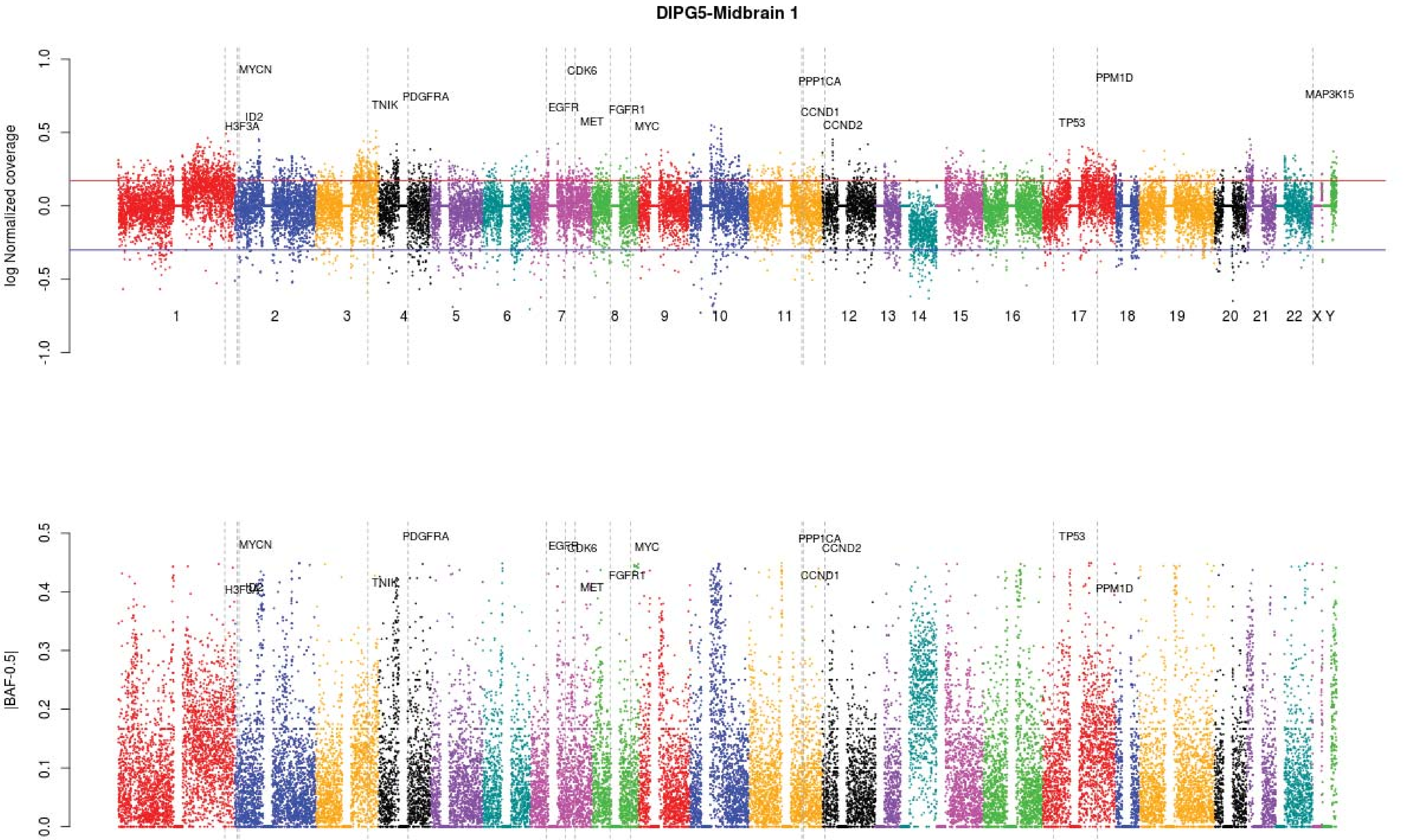

DIPG5-Midbrain 1
log Normalized coverage
1.0
0.5
0.0
-0.5
-1.0
MYCN
ID2
H3F3A
TNIK
PDGFRA
EGFR
CDK6
MET
FGFR1
MYC
PPP1CA
CCND1
CCND2
TP53
PPM1D
MAP3K15
1 2 3 4 5 6 7 8 9 10 11 12 13 14 15 16 17 18 19 20 21 22 X Y
|BAF-0.5|
0.5
0.4
0.3
0.2
0.1
0.0

DIPG5-Pons 1

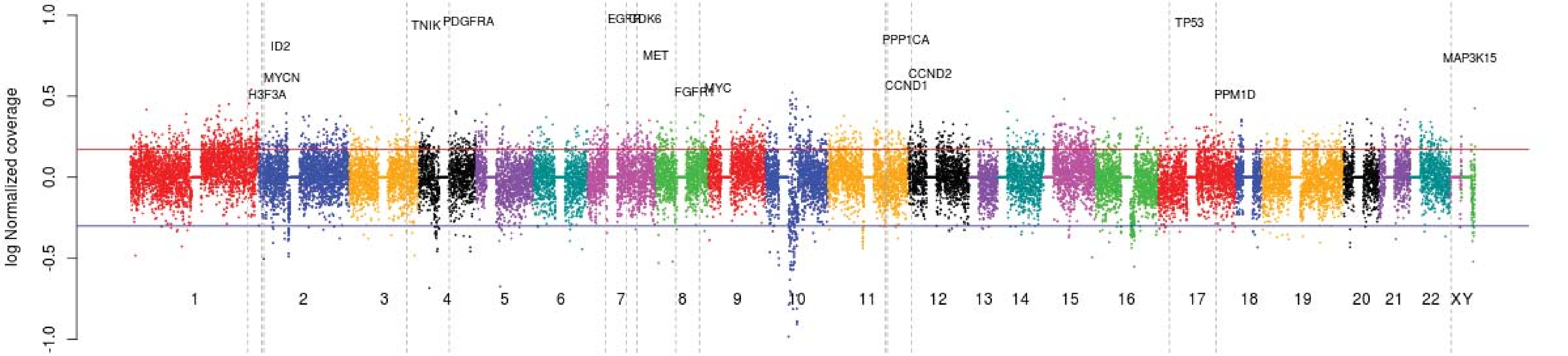

log Normalized coverage
1.0
0.5
0.0
-0.5
-1.0
H3F3A
MYCN
ID2
TNIK
PDGFRA
EGFR
CDK6
MET
FGFR1
MYC
PPP1CA
CCND2
CCND1
TP53
PPM1D
MAP3K15
1
2
3
4
5
6
7
8
9
10
11
12
13
14
15
16
17
18
19
20
21
22
XY

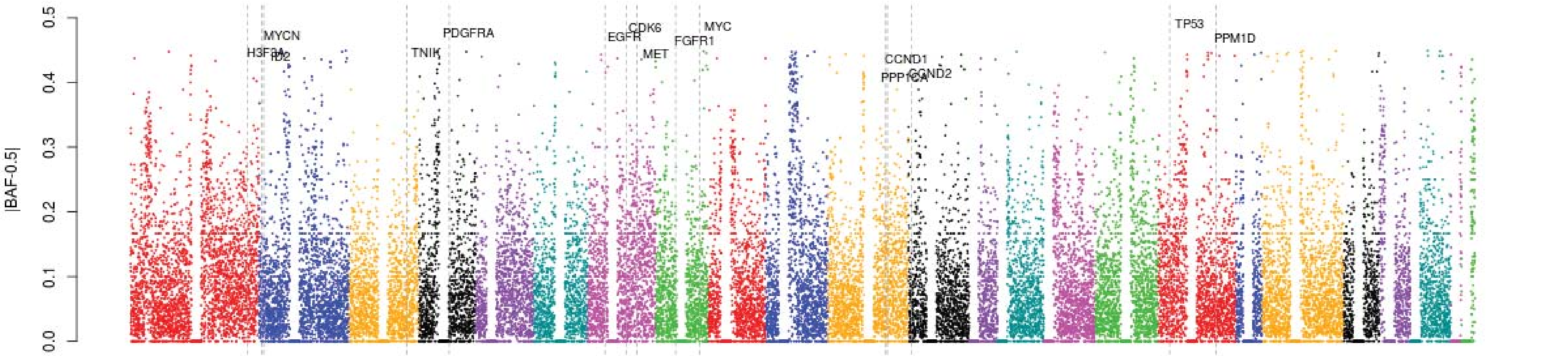

|BAF-0.5|
0.5
0.4
0.3
0.2
0.1
0.0
H3F3A
MYCN
ID2
TNIK
PDGFRA
EGFR
CDK6
MET
FGFR1
MYC
CCND1
PPP1CA
CCND2
TP53
PPM1D

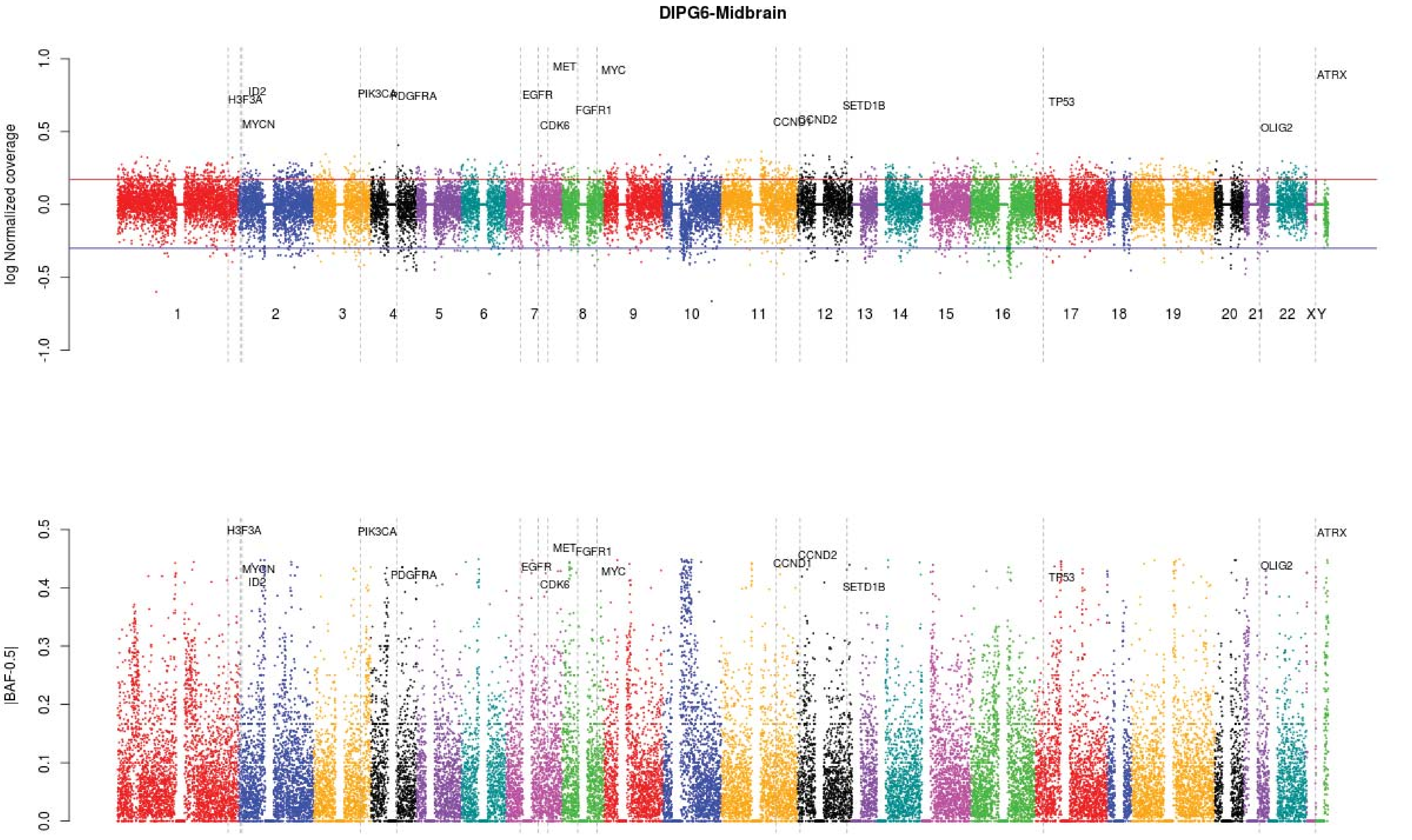

DIPG6-Midbrain
log Normalized coverage
|BAF-0.5|
H3F3A
ID2
MYCN
PIK3CA
PDGFRA
EGFR
CDK6
MET
FGFR1
MYC
CCND1
CCND2
SETD1B
TP53
OLIG2
ATRX
1
2
3
4
5
6
7
8
9
10
11
12
13
14
15
16
17
18
19
20
21
22
XY

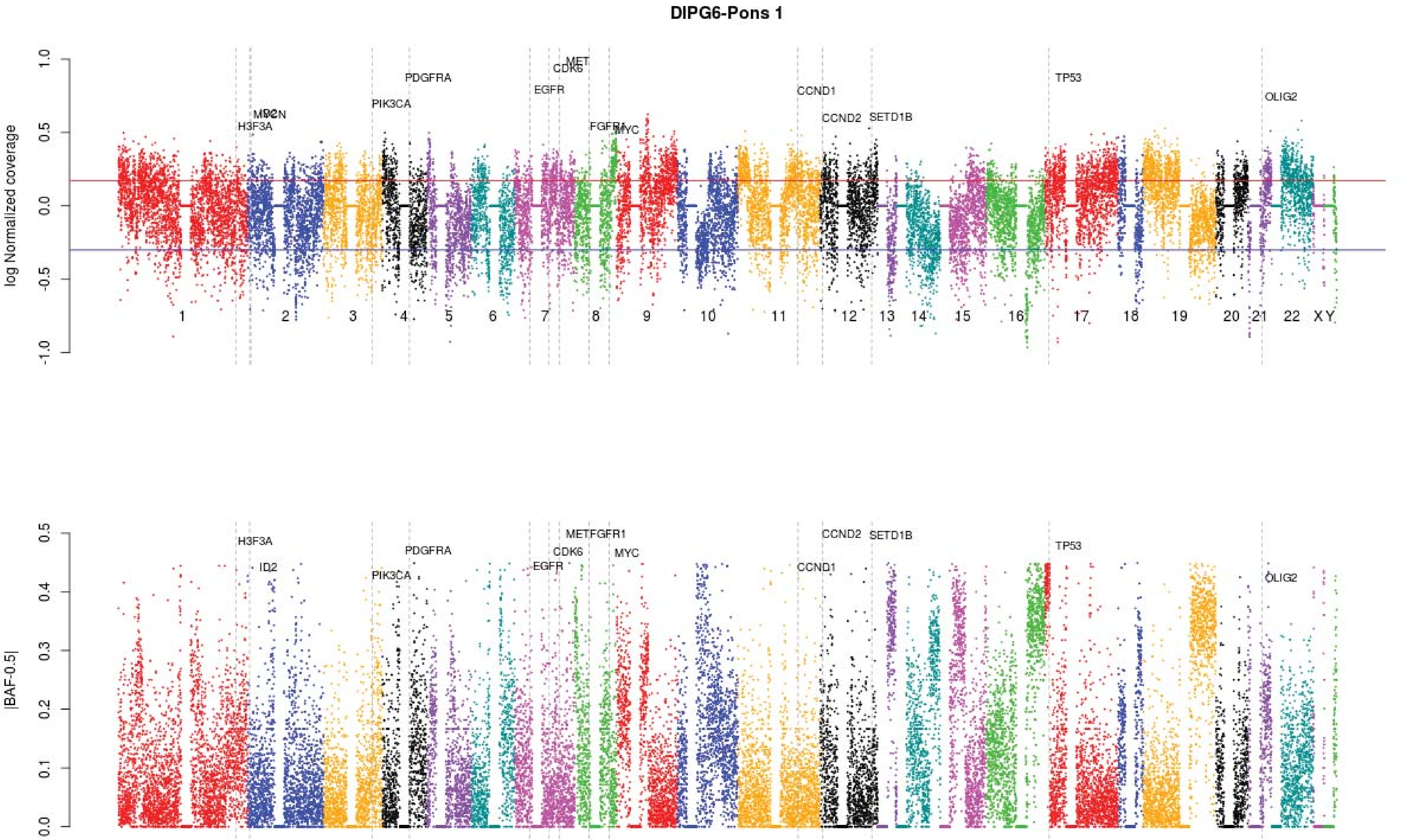

DIPG6-Pons 1
log Normalized coverage
|BAF-0.5|
H3F3A
ID2
MYCN
PIK3CA
PDGFRA
EGFR
CDK6
MET
FGFR1
MYC
CCND1
CCND2
SETD1B
TP53
OLIG2
1 2 3 4 5 6 7 8 9 10 11 12 13 14 15 16 17 18 19 20 21 22 X Y

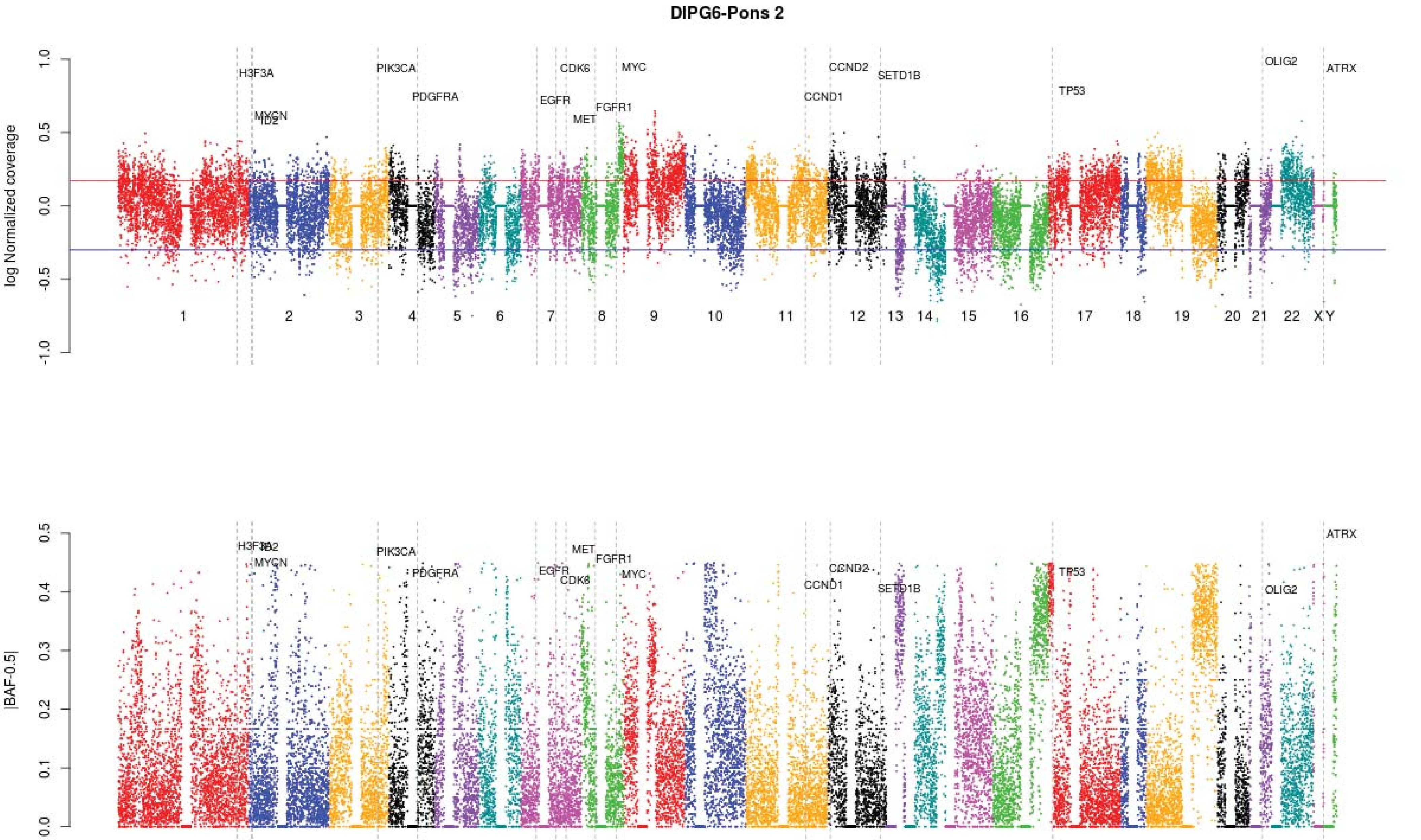

DIPG6-Pons 2
log Normalized coverage
|BAF-0.5|
H3F3A
MYCN
ID2
PIK3CA
PDGFRA
EGFR
CDK6
MET
FGFR1
MYC
CCND1
CCND2
SETD1B
TP53
OLIG2
ATRX
1
2
3
4
5
6
7
8
9
10
11
12
13
14
15
16
17
18
19
20
21
22
X
Y

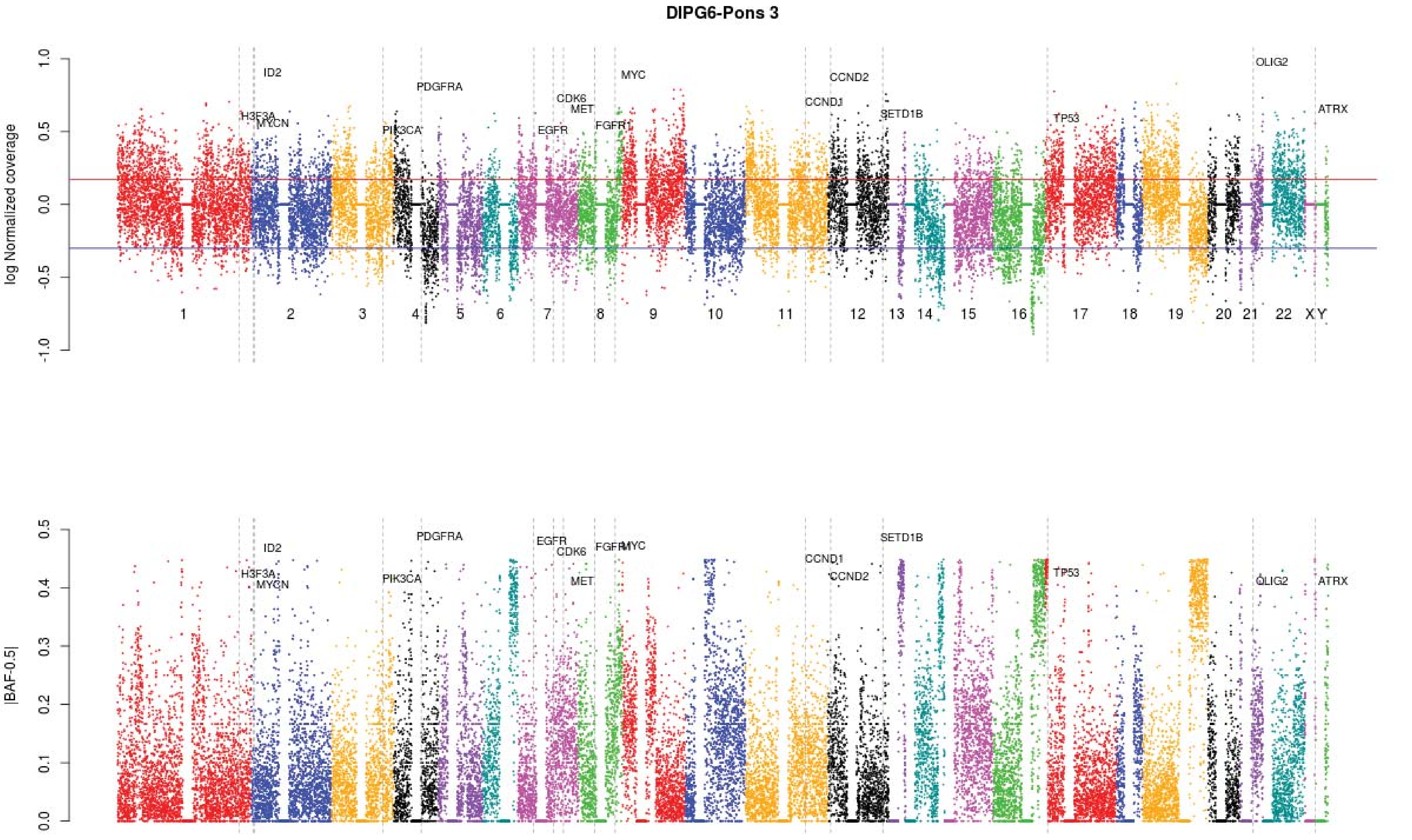
DIPG6-Pons 3
log Normalized coverage
|BAF-0.5|
ID2
H3F3A
MYCN
PIK3CA
PDGFRA
EGFR
CDK6
MET
FGFR1
MYC
CCND1
CCND2
SETD1B
TP53
OLIG2
ATRX
1
2
3
4
5
6
7
8
9
10
11
12
13
14
15
16
17
18
19
20
21
22
X
Y

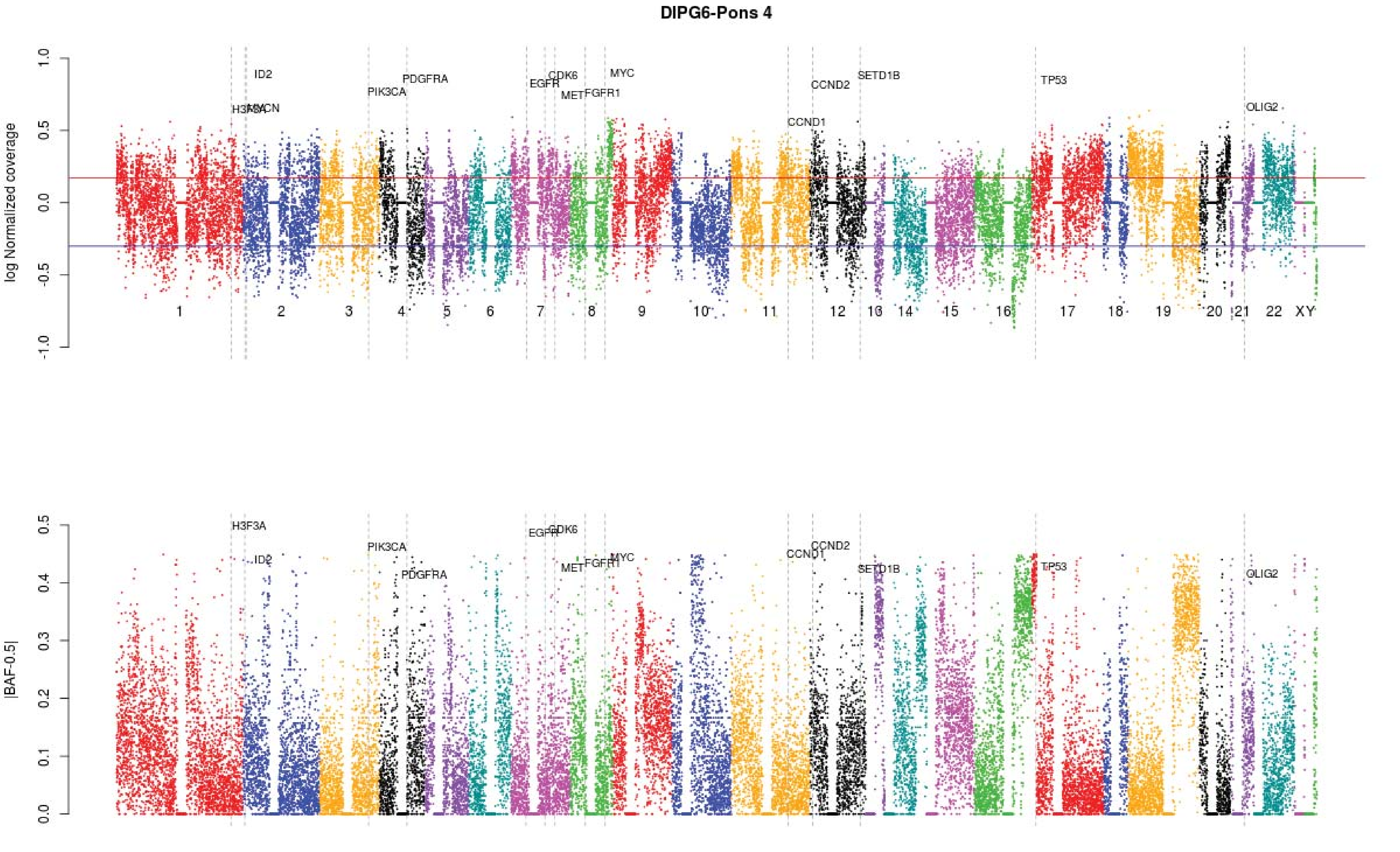
DIPG6-Pons 4
log Normalized coverage
|BAF-0.5|
H3F3A
ID2
MYCN
PIK3CA
PDGFRA
EGFR
CDK6
MET
FGFR1
MYC
CCND1
CCND2
SETD1B
TP53
OLIG2
1
2
3
4
5
6
7
8
9
10
11
12
13
14
15
16
17
18
19
20
21
22
XY

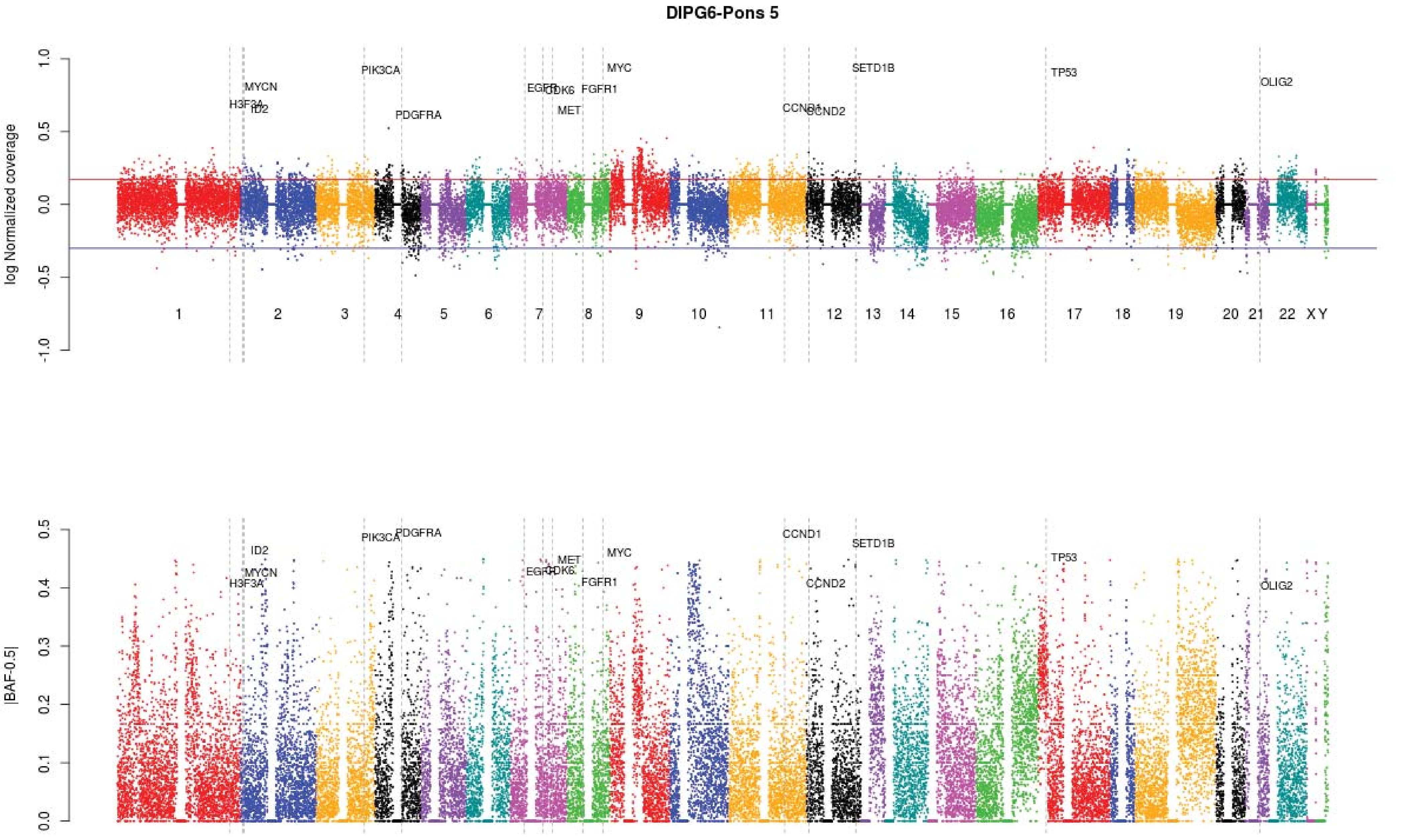
DIPG6-Pons 5
log Normalized coverage
|BAF-0.5|
H3F3A
MYCN
ID2
PIK3CA
PDGFRA
EGFR
CDK6
MET
FGFR1
MYC
CCND1
CCND2
SETD1B
TP53
OLIG2
1
2
3
4
5
6
7
8
9
10
11
12
13
14
15
16
17
18
19
20
21
22
X
Y

DIPG7-Cerebellum 1

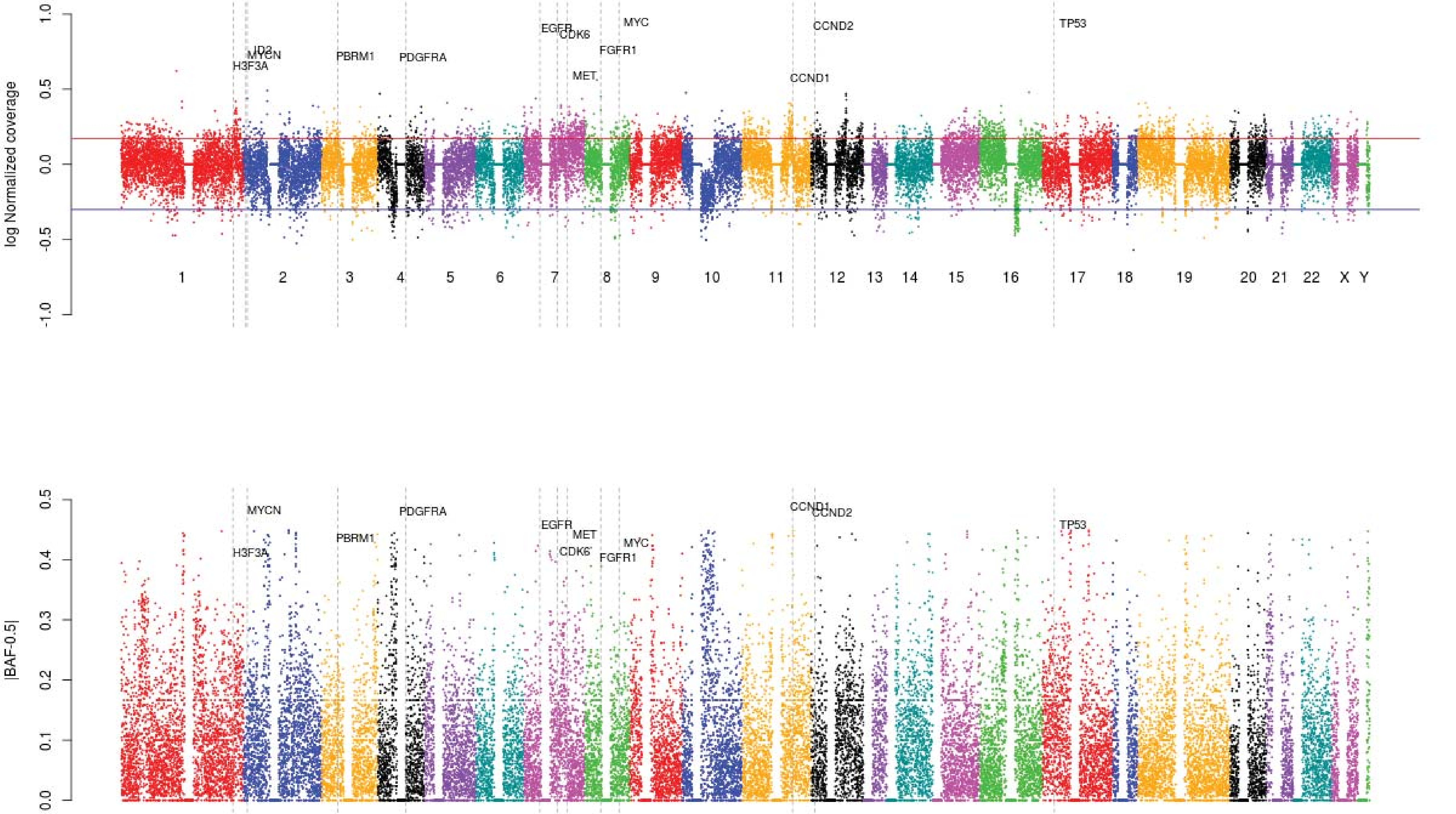

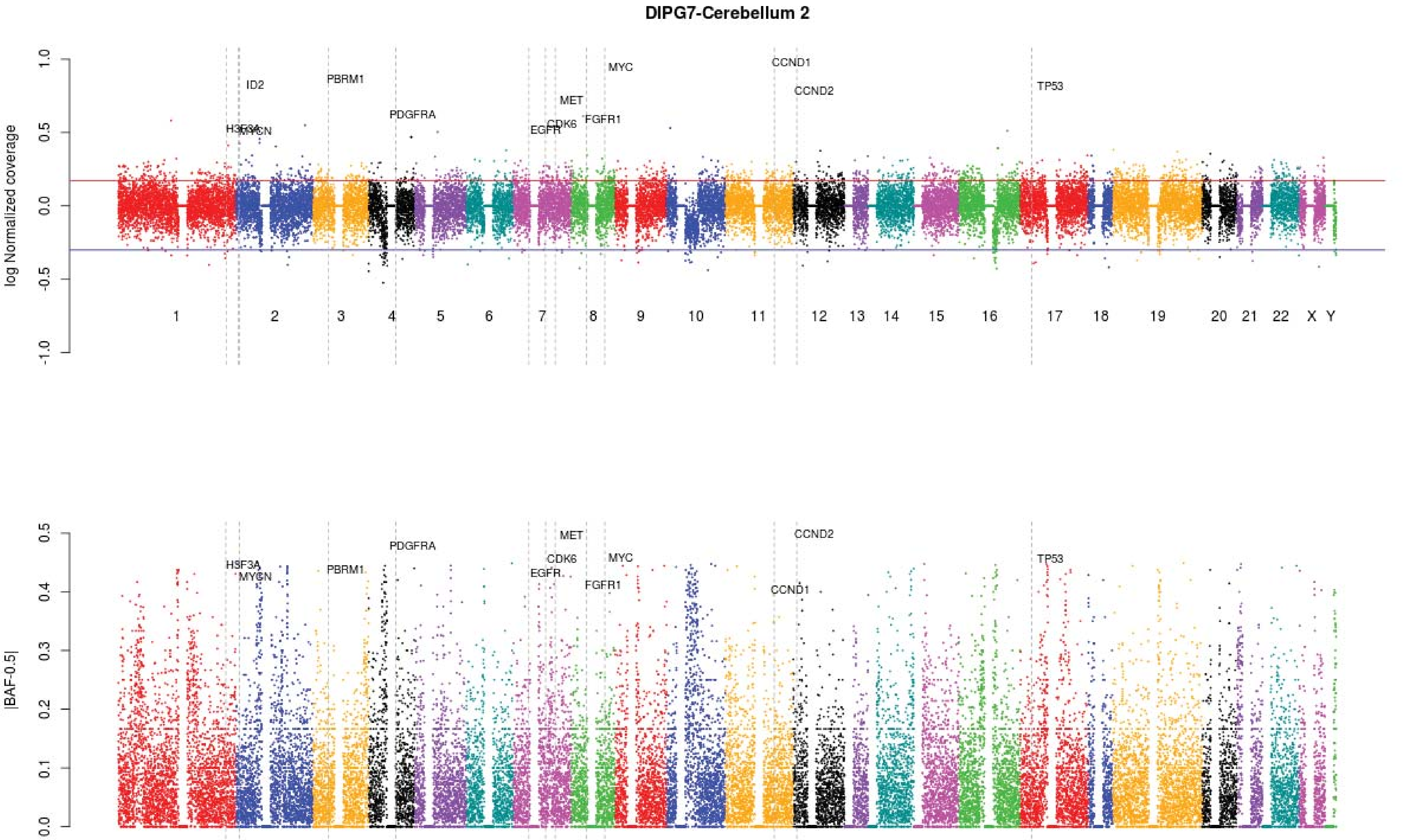
DIPG7-Cerebellum 2
log Normalized coverage
|BAF-0.5|
ID2
H3F3A
MYCN
PBRM1
PDGFRA
EGFR
CDK6
MET
FGFR1
MYC
CCND1
CCND2
TP53
1
2
3
4
5
6
7
8
9
10
11
12
13
14
15
16
17
18
19
20
21
22
X
Y

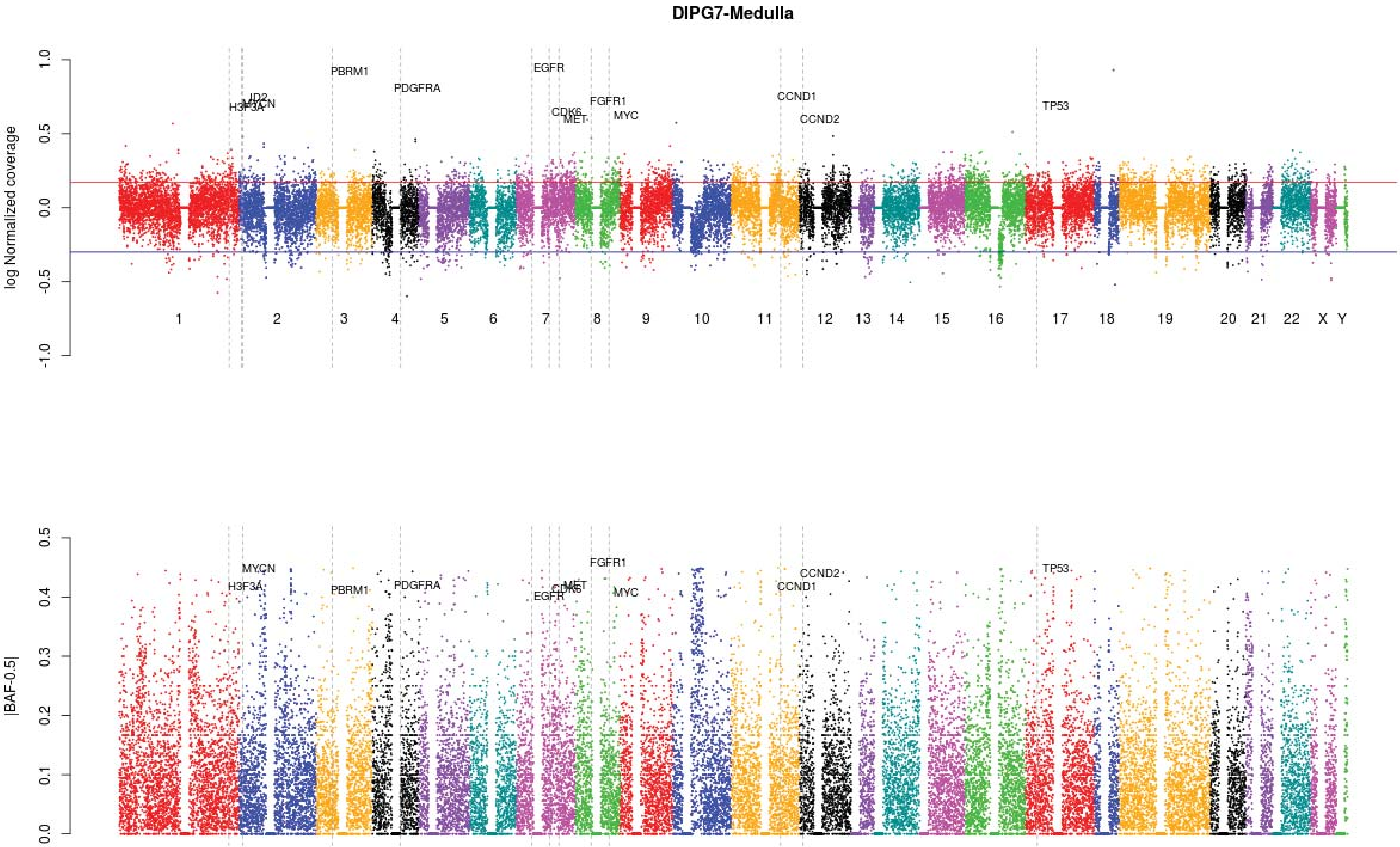
DIPG7-Medulla
log Normalized coverage
|BAF-0.5|
H3F3A
ID2
MYCN
PBRM1
PDGFRA
EGFR
CDK6
MET
FGFR1
MYC
CCND1
CCND2
TP53
1
2
3
4
5
6
7
8
9
10
11
12
13
14
15
16
17
18
19
20
21
22
X
Y

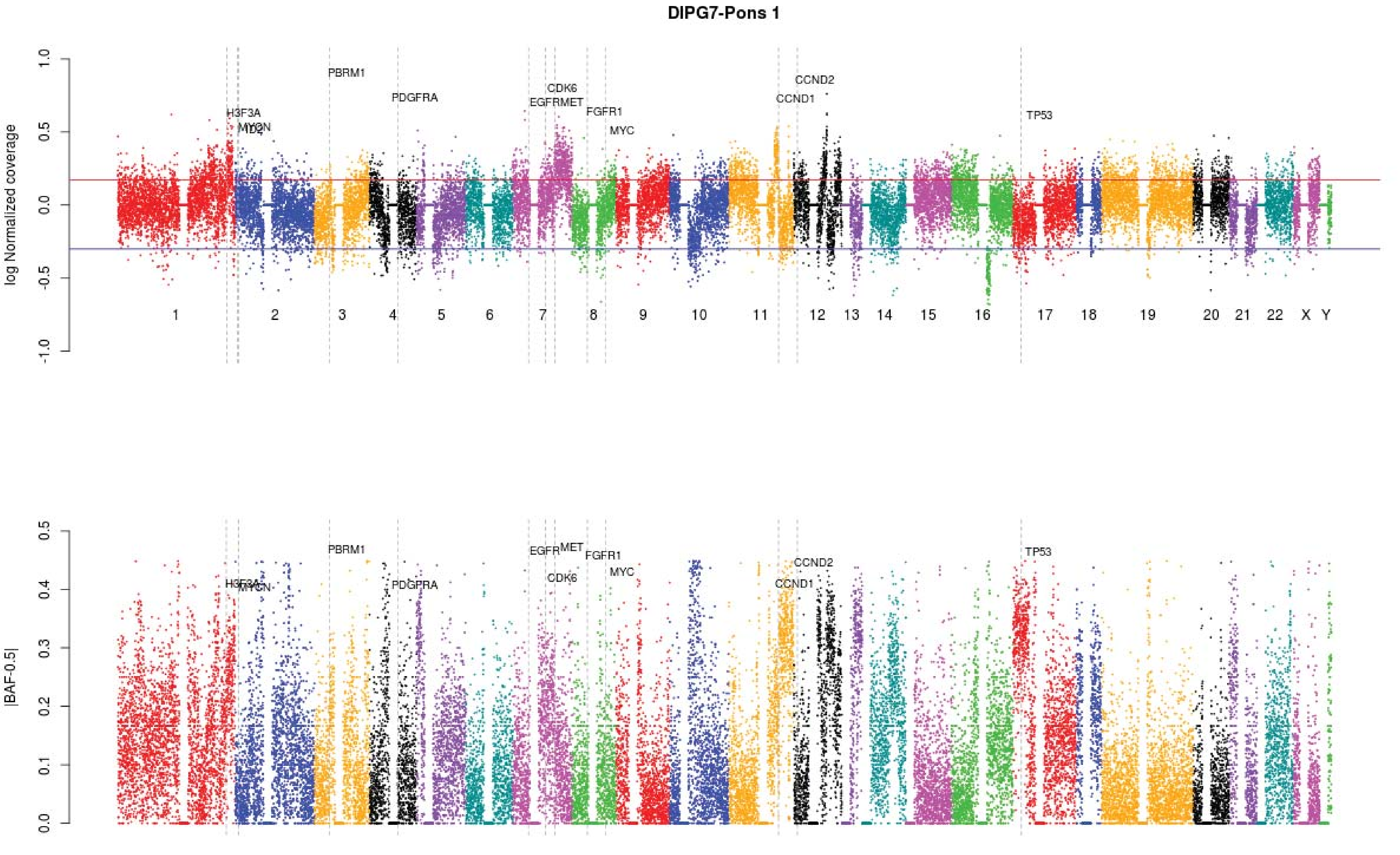
DIPG7-Pons 1
log Normalized coverage
1.0
0.5
0.0
-0.5
-1.0
H3F3A
MYCN
PBRM1
PDGFRA
CDK6
EGFR
MET
FGFR1
MYC
CCND1
CCND2
TP53
1
2
3
4
5
6
7
8
9
10
11
12
13
14
15
16
17
18
19
20
21
22
X
Y
|BAF-0.5|
0.5
0.4
0.3
0.2
0.1
0.0

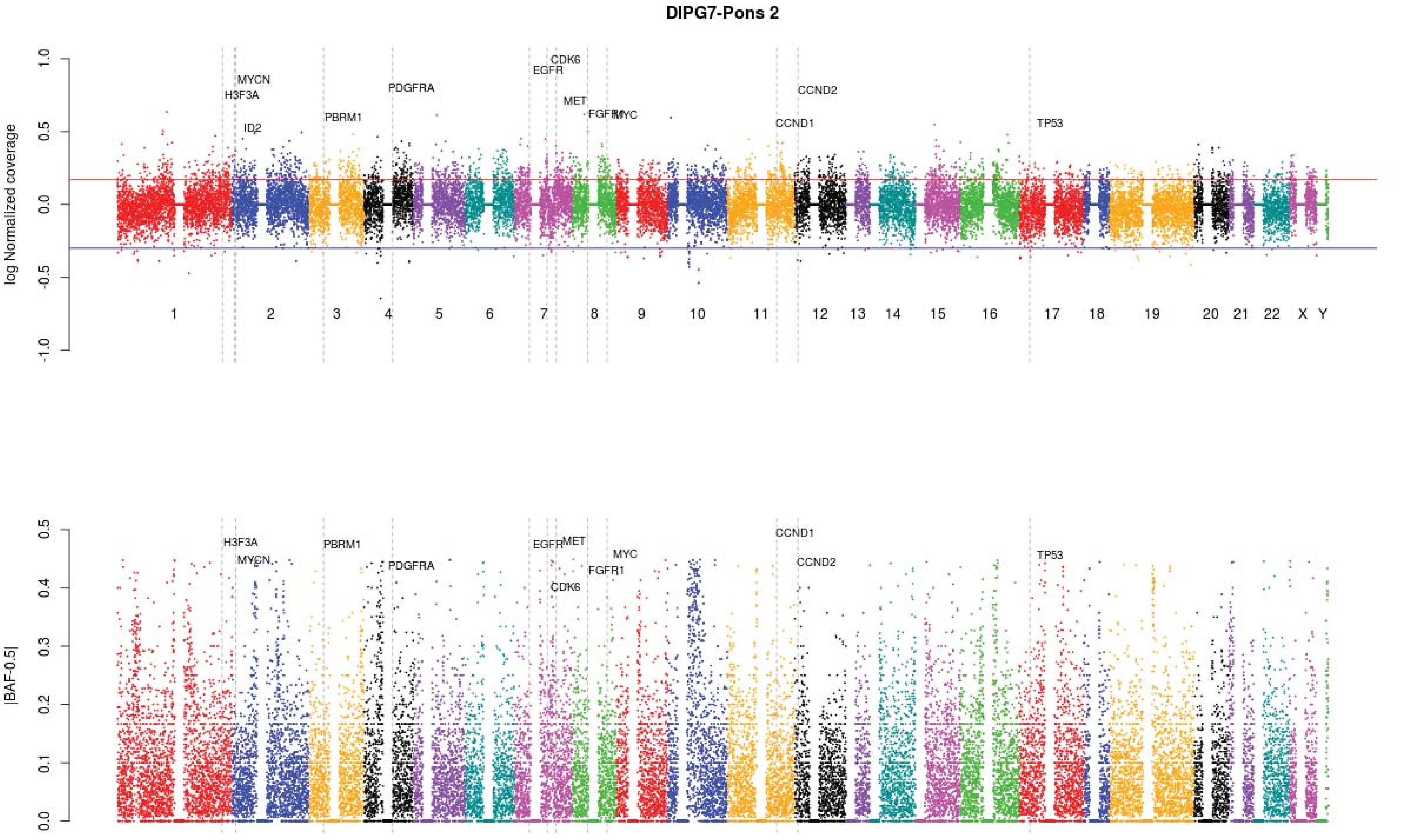
DIPG7-Pons 2
log Normalized coverage
1.0
0.5
0.0
-0.5
-1.0
MYCN
H3F3A
ID2
PBRM1
PDGFRA
CDK6
EGFR
MET
FGFR1
MYC
CCND2
CCND1
TP53
1
2
3
4
5
6
7
8
9
10
11
12
13
14
15
16
17
18
19
20
21
22
X
Y
|BAF-0.5|
0.5
0.4
0.3
0.2
0.1
0.0

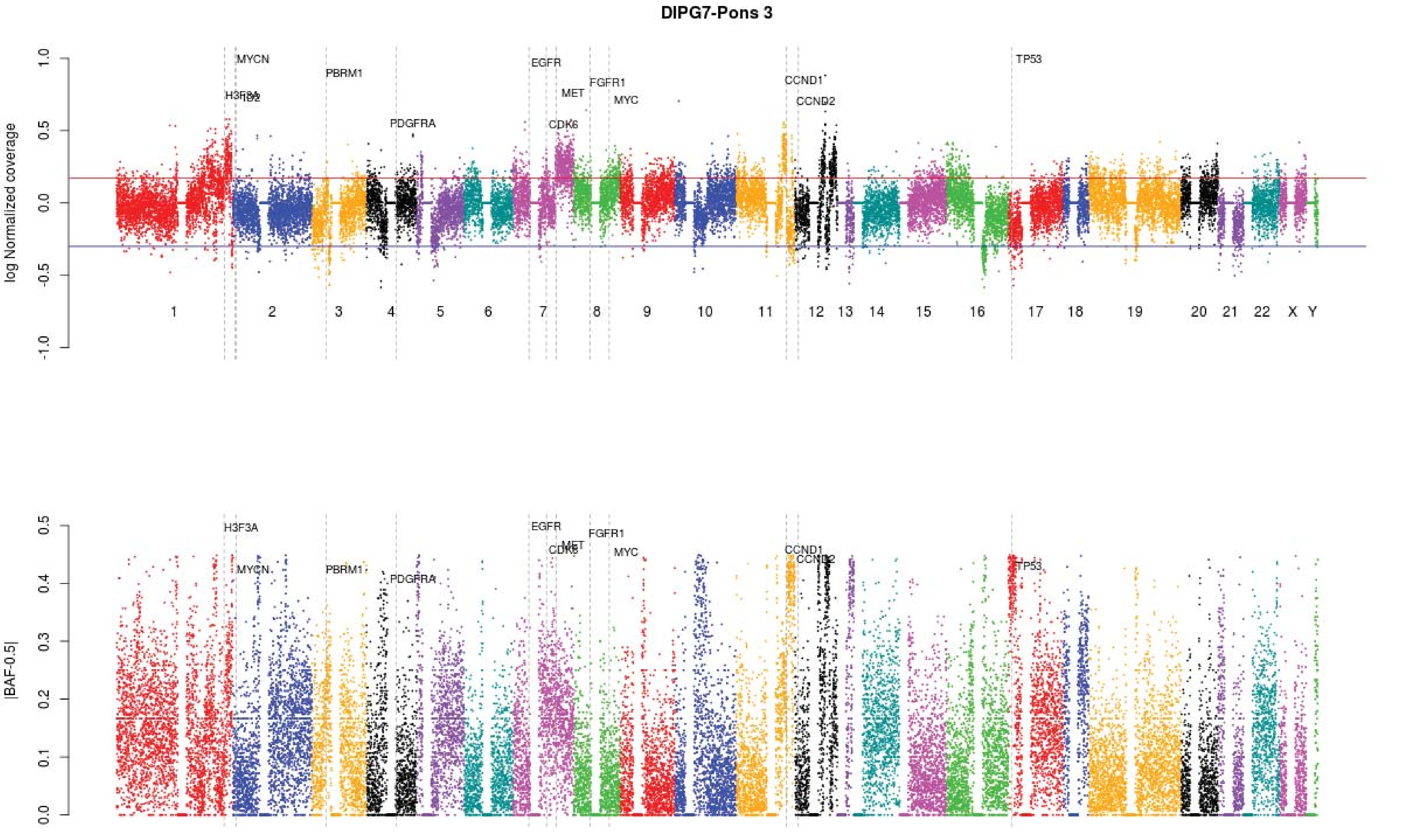

DIPG7-Pons 3
log Normalized coverage
|BAF-0.5|
MYCN
H3F3A
ID2
PBRM1
PDGFRA
EGFR
MET
CDK6
FGFR1
MYC
CCND1
CCND2
TP53
1 2 3 4 5 6 7 8 9 10 11 12 13 14 15 16 17 18 19 20 21 22 X Y

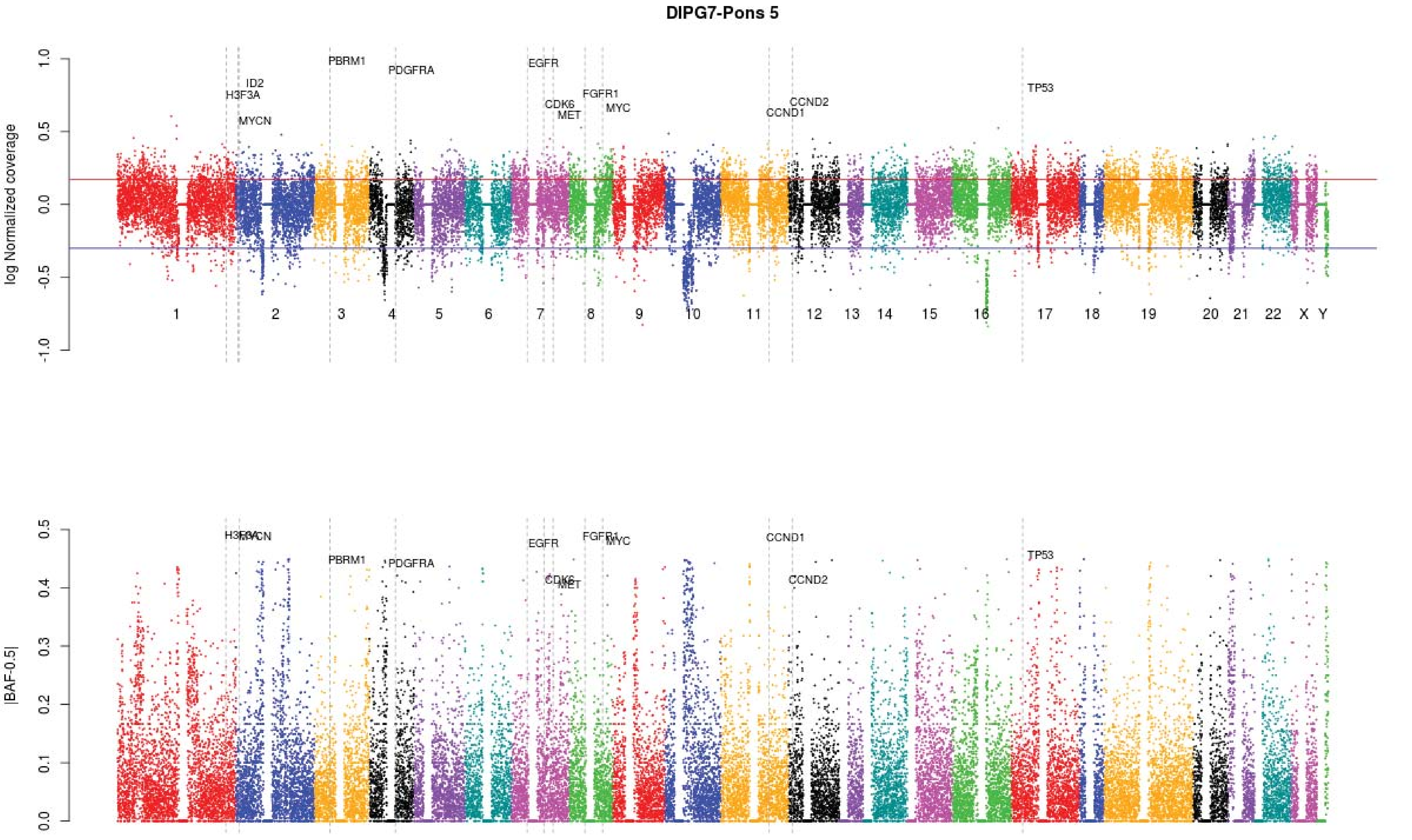

DIPG7-Pons 5
log Normalized coverage
1.0
0.5
0.0
-0.5
-1.0
H3F3A
ID2
MYCN
PBRM1
PDGFRA
EGFR
CDK6
MET
FGFR1
MYC
CCND1
CCND2
TP53
1
2
3
4
5
6
7
8
9
10
11
12
13
14
15
16
17
18
19
20
21
22
X
Y
|BAF-0.5|
0.5
0.4
0.3
0.2
0.1
0.0

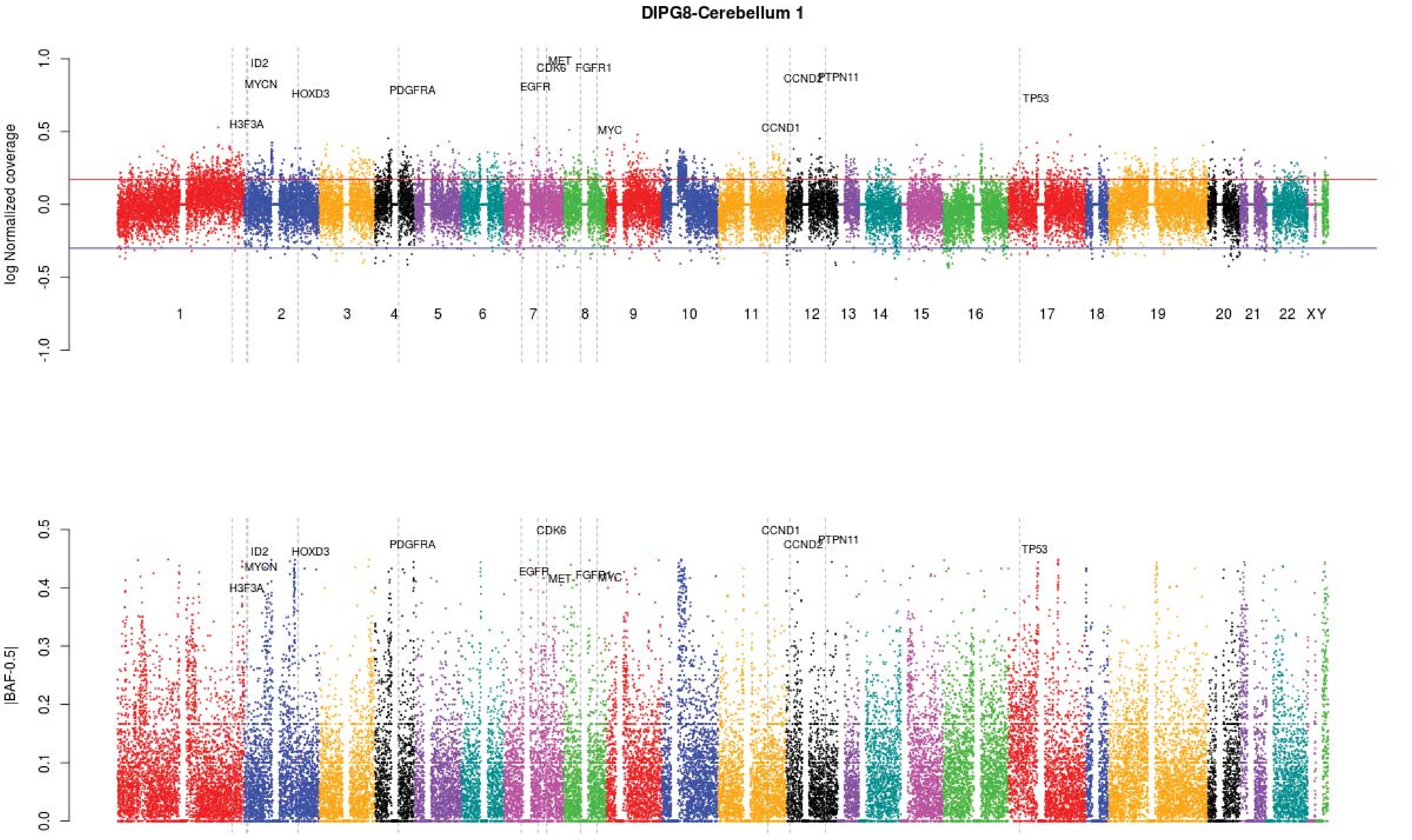
DIPG8-Cerebellum 1
log Normalized coverage
|BAF-0.5|
H3F3A
ID2
MYCN
HOXD3
PDGFRA
EGFR
CDK6
MET
FGFR1
MYC
CCND1
CCND2
PTPN11
TP53
1
2
3
4
5
6
7
8
9
10
11
12
13
14
15
16
17
18
19
20
21
22
XY

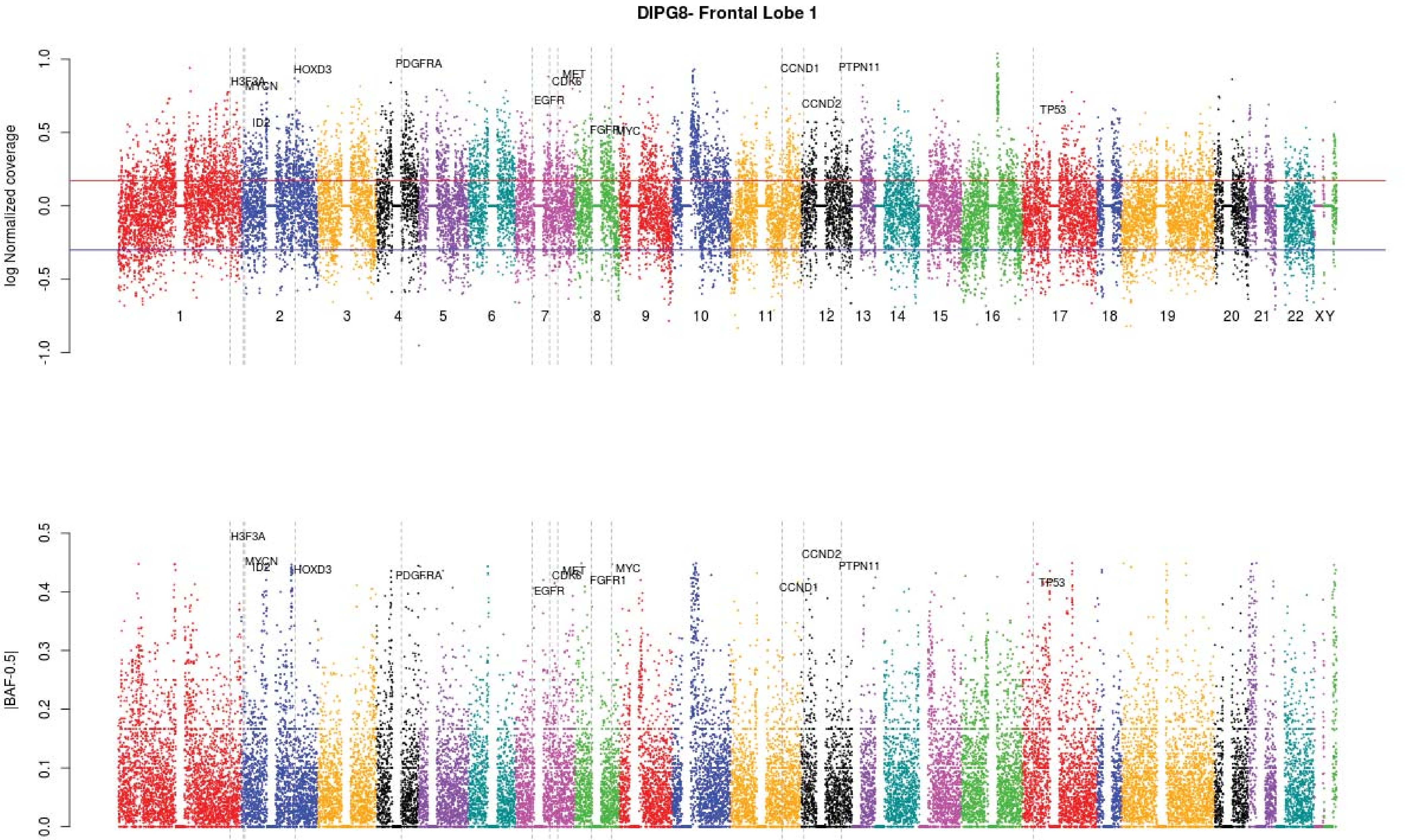
DIPG8- Frontal Lobe 1
log Normalized coverage
1.0
0.5
0.0
-0.5
-1.0
H3F3A
MYCN
ID2
HOXD3
PDGFRA
MET
CDK6
EGFR
FGFR1
MYC
CCND1
CCND2
PTPN11
TP53
1 2 3 4 5 6 7 8 9 10 11 12 13 14 15 16 17 18 19 20 21 22 XY
|BAF-0.5|
0.5
0.4
0.3
0.2
0.1
0.0

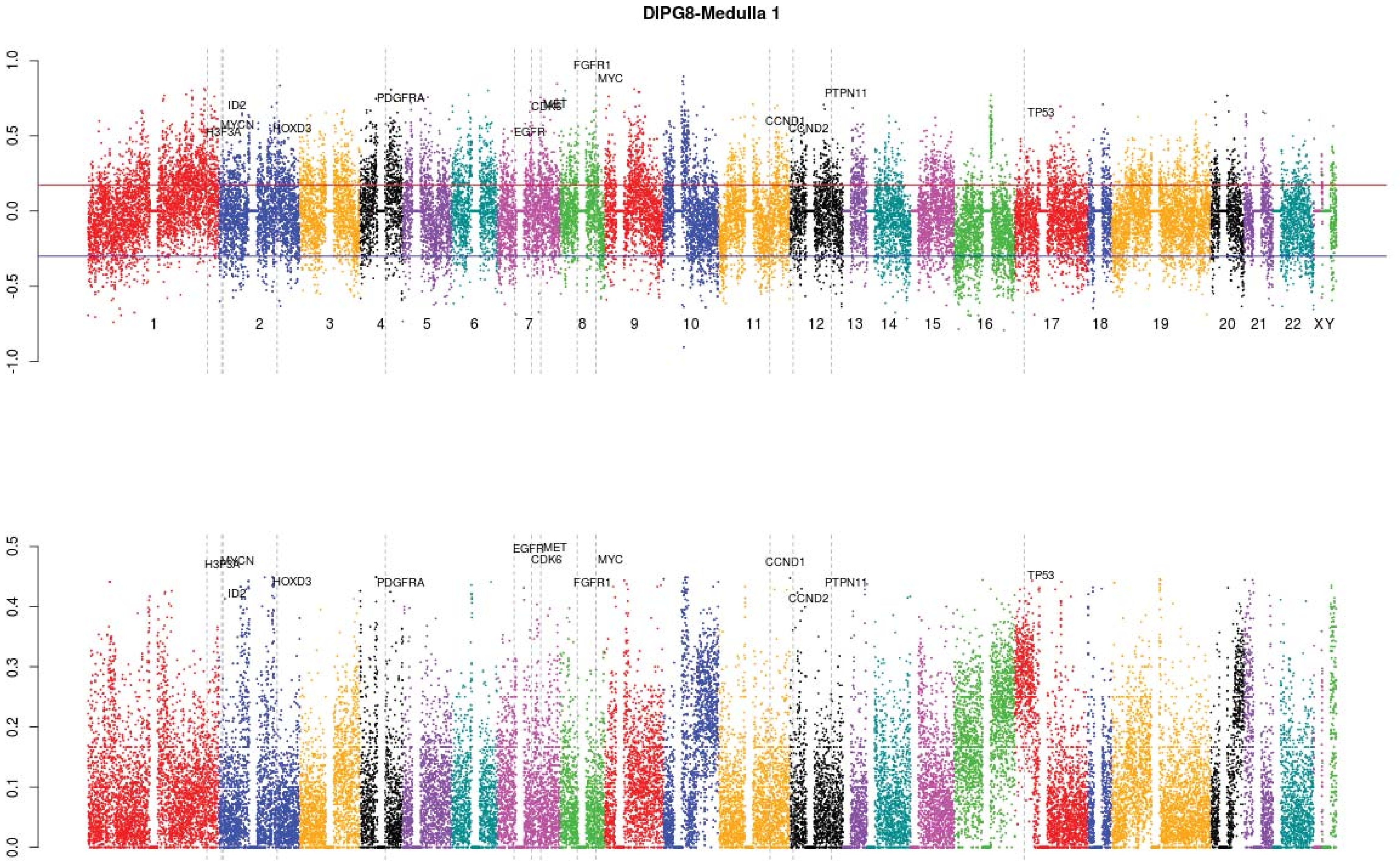

DIPG8-Medulla 1
log Normalized coverage
1.0
0.5
0.0
-0.5
-1.0
FGFR1
MYC
PTPN11
ID2
PDGFRA
MET
CDK6
TP53
CCND1
MYCN
H3F3A
HOXD3
EGFR
CCND2
1
2
3
4
5
6
7
8
9
10
11
12
13
14
15
16
17
18
19
20
21
22
XY
|BAF-0.5|
0.5
0.4
0.3
0.2
0.1
0.0

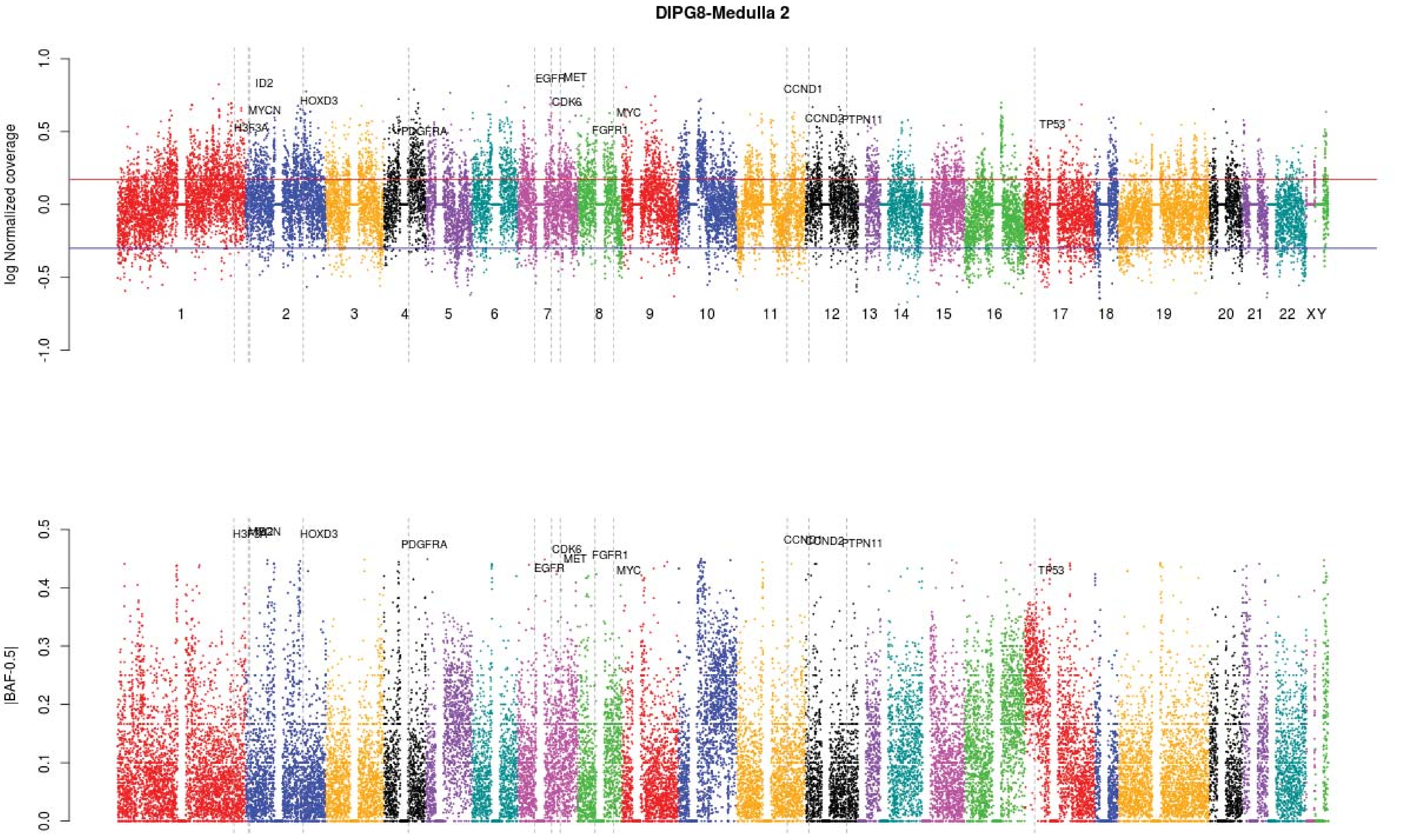
DIPG8-Medulla 2
log Normalized coverage
1.0
0.5
0.0
-0.5
-1.0
ID2
MYCN
H3F3A
HOXD3
PDGFRA
EGFR
MET
CDK6
FGFR1
MYC
CCND1
CCND2
PTPN11
TP53
1 2 3 4 5 6 7 8 9 10 11 12 13 14 15 16 17 18 19 20 21 22 XY
|BAF-0.5|
0.5
0.4
0.3
0.2
0.1
0.0

DIPG8-Medulla 3

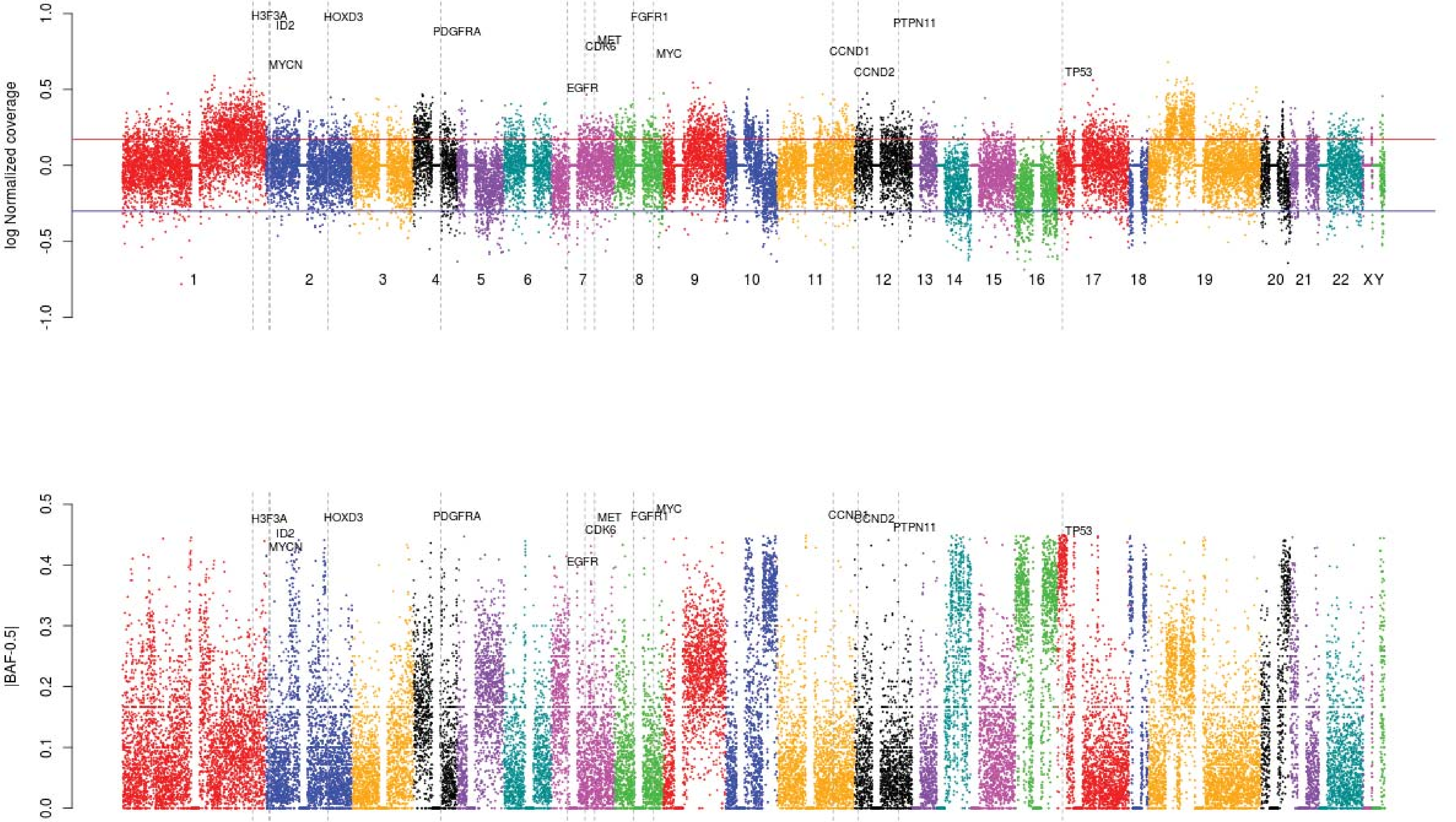

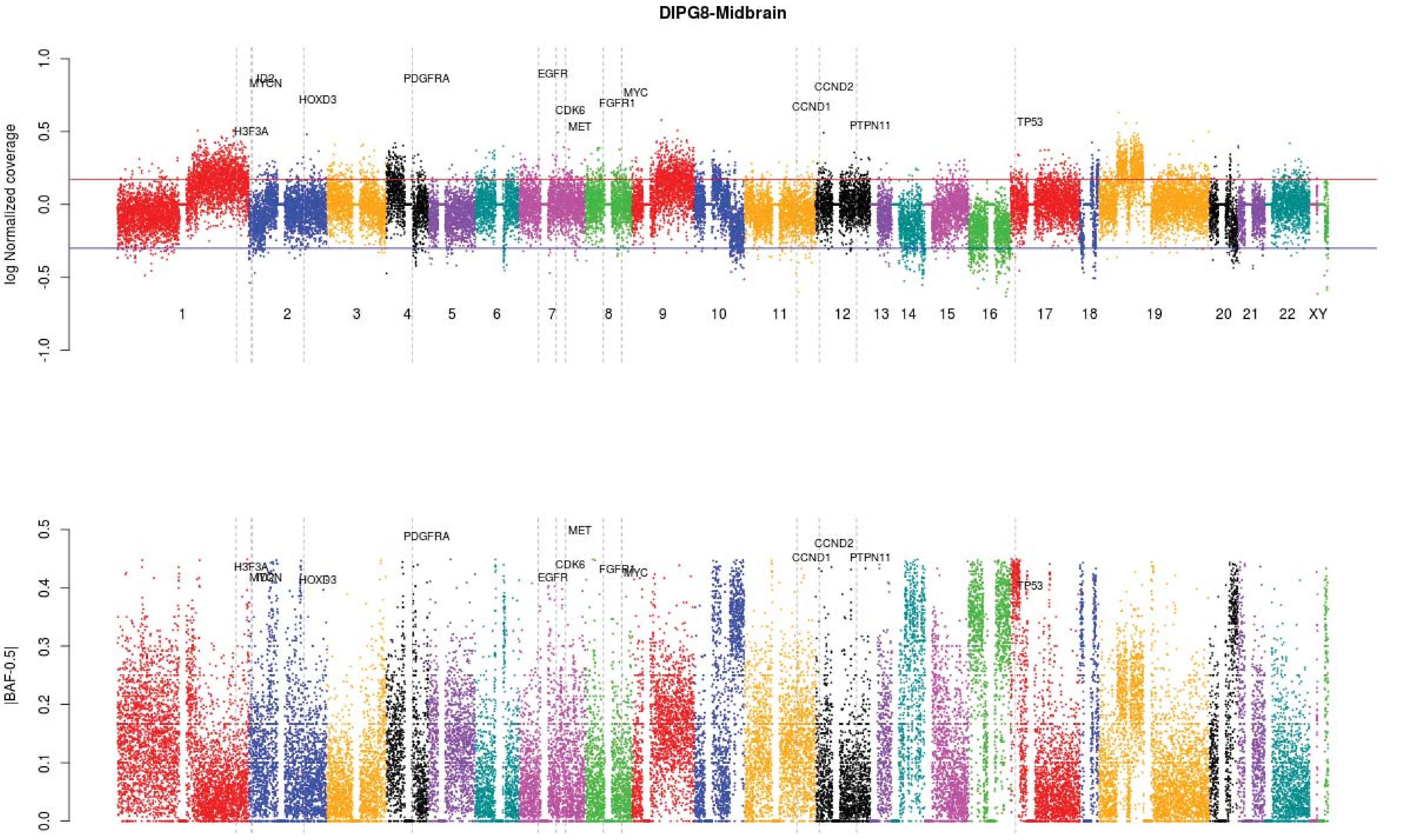
DIPG8-Midbrain
log Normalized coverage
1.0
0.5
0.0
-0.5
-1.0
H3F3A
ID2
MYCN
HOXD3
PDGFRA
EGFR
CDK6
MET
FGFR1
MYC
CCND1
CCND2
PTPN11
TP53
1
2
3
4
5
6
7
8
9
10
11
12
13
14
15
16
17
18
19
20
21
22
XY
|BAF-0.5|
0.5
0.4
0.3
0.2
0.1
0.0

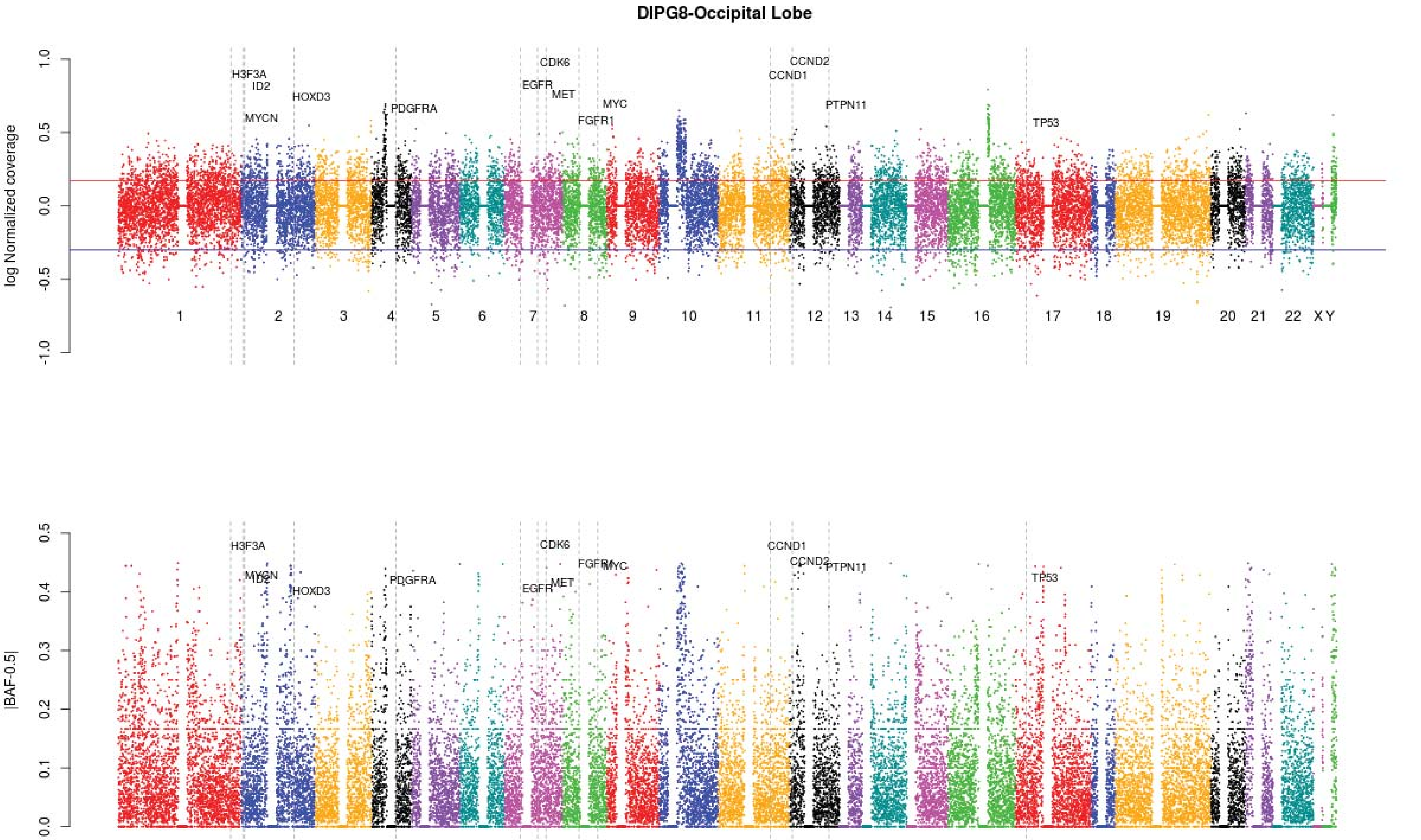

DIPG8-Occipital Lobe
log Normalized coverage
1.0
0.5
0.0
-0.5
-1.0
H3F3A
ID2
MYCN
HOXD3
PDGFRA
CDK6
EGFR
MET
FGFR1
MYC
CCND2
CCND1
PTPN11
TP53
1 2 3 4 5 6 7 8 9 10 11 12 13 14 15 16 17 18 19 20 21 22 X Y
|BAF-0.5|
0.5
0.4
0.3
0.2
0.1
0.0

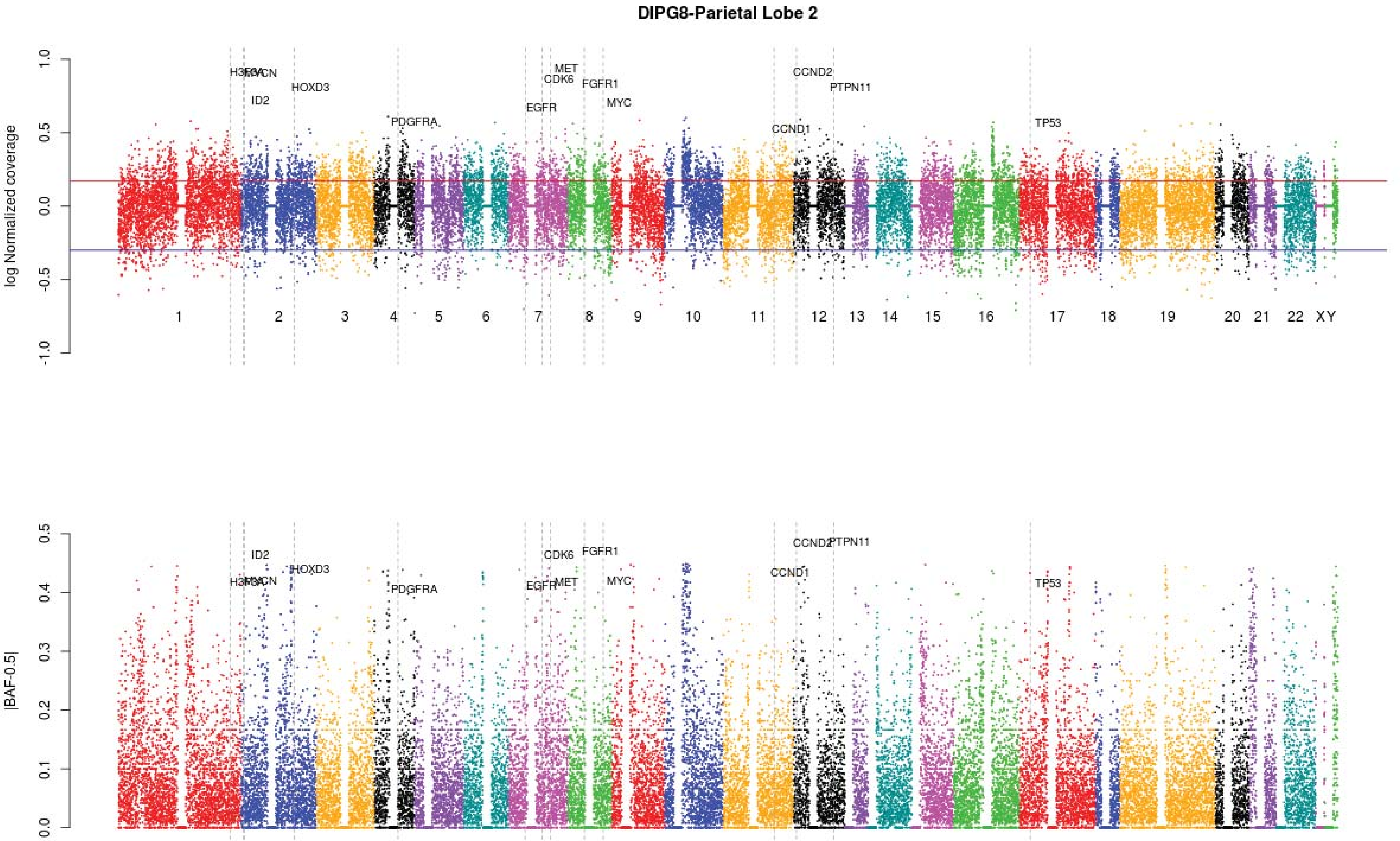
DIPG8-Parietal Lobe 2
log Normalized coverage
1.0
0.5
0.0
-0.5
-1.0
H3F3A
MYCN
ID2
HOXD3
PDGFRA
EGFR
CDK6
MET
FGFR1
MYC
CCND1
CCND2
PTPN11
TP53
1
2
3
4
5
6
7
8
9
10
11
12
13
14
15
16
17
18
19
20
21
22
XY
|BAF-0.5|
0.5
0.4
0.3
0.2
0.1
0.0

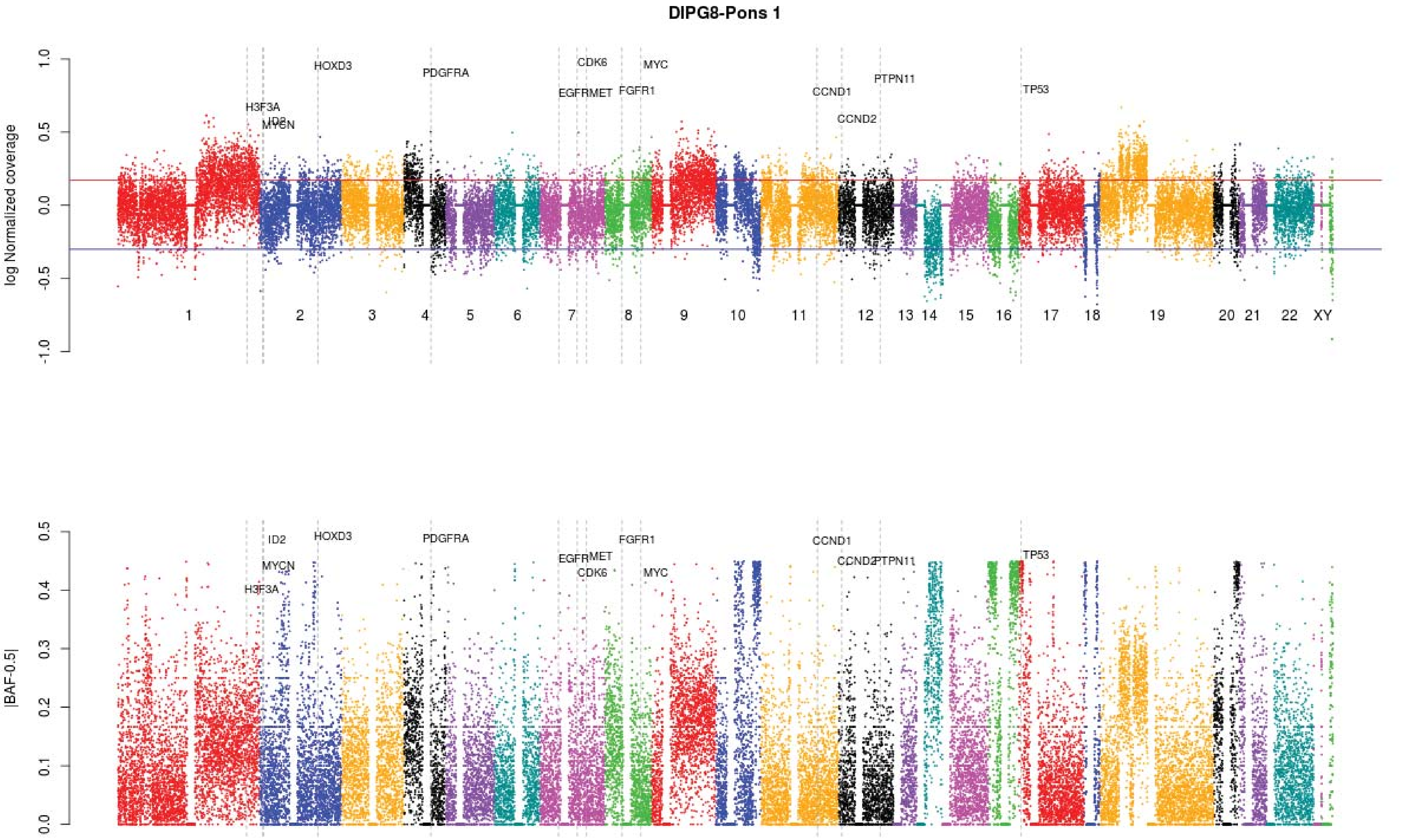
DIPG8-Pons 1
log Normalized coverage
1.0
0.5
0.0
-0.5
-1.0
H3F3A
ID2
MYCN
HOXD3
PDGFRA
EGFR
MET
CDK6
FGFR1
MYC
CCND1
CCND2
PTPN11
TP53
1
2
3
4
5
6
7
8
9
10
11
12
13
14
15
16
17
18
19
20
21
22
XY
|BAF-0.5|
0.5
0.4
0.3
0.2
0.1
0.0

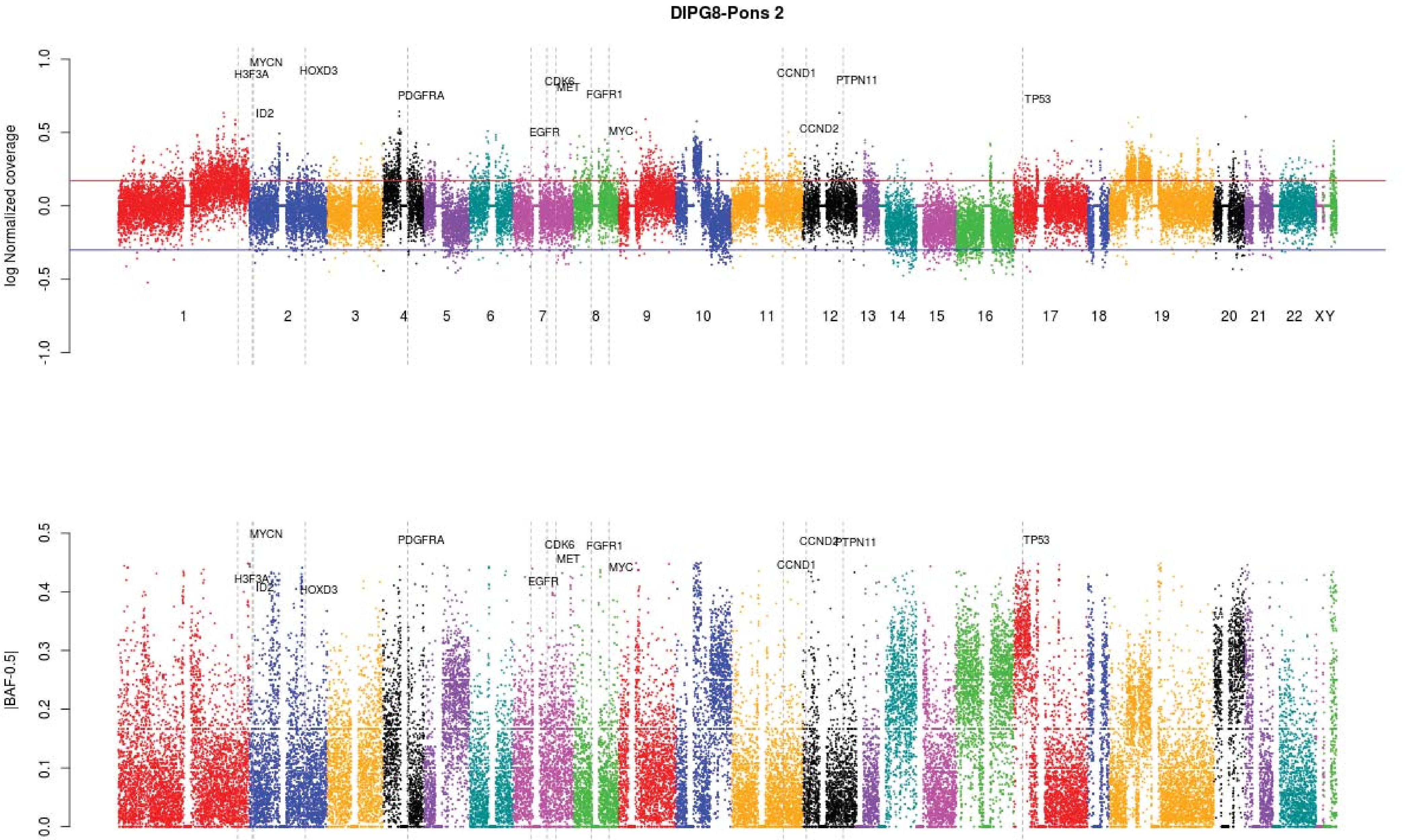
DIPG8-Pons 2
log Normalized coverage
1.0
0.5
0.0
-0.5
-1.0
MYCN
H3F3A
HOXD3
ID2
PDGFRA
CDK6
MET
FGFR1
EGFR
MYC
CCND1
CCND2
PTPN11
TP53
1
2
3
4
5
6
7
8
9
10
11
12
13
14
15
16
17
18
19
20
21
22
XY
|BAF-0.5|
0.5
0.4
0.3
0.2
0.1
0.0

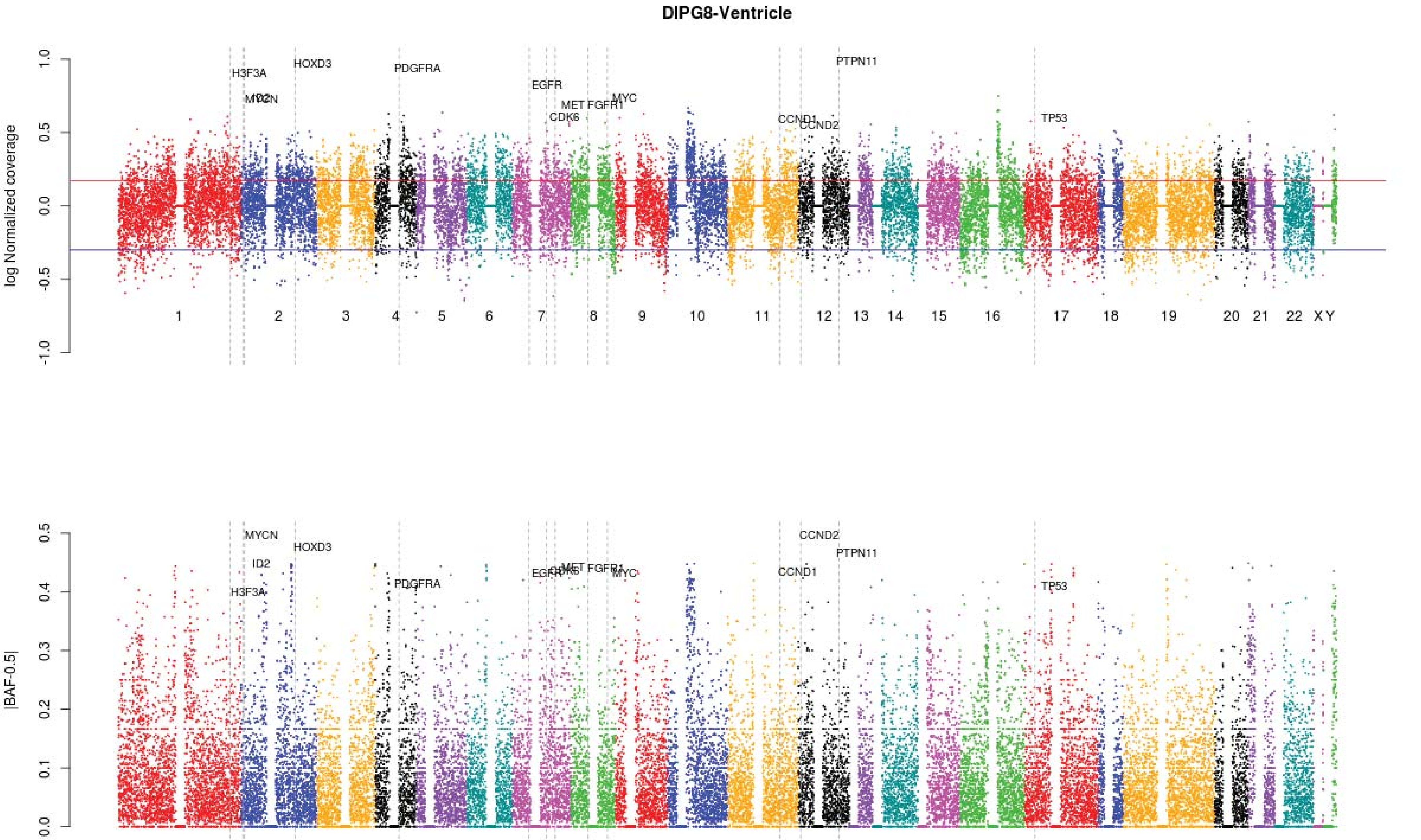
DIPG8-Ventricle
log Normalized coverage
1.0
0.5
0.0
-0.5
-1.0
H3F3A
MYCN
ID2
HOXD3
PDGFRA
EGFR
CDK6
MET
FGFR1
MYC
CCND1
CCND2
PTPN11
TP53
1
2
3
4
5
6
7
8
9
10
11
12
13
14
15
16
17
18
19
20
21
22
X
Y
|BAF-0.5|
0.5
0.4
0.3
0.2
0.1
0.0

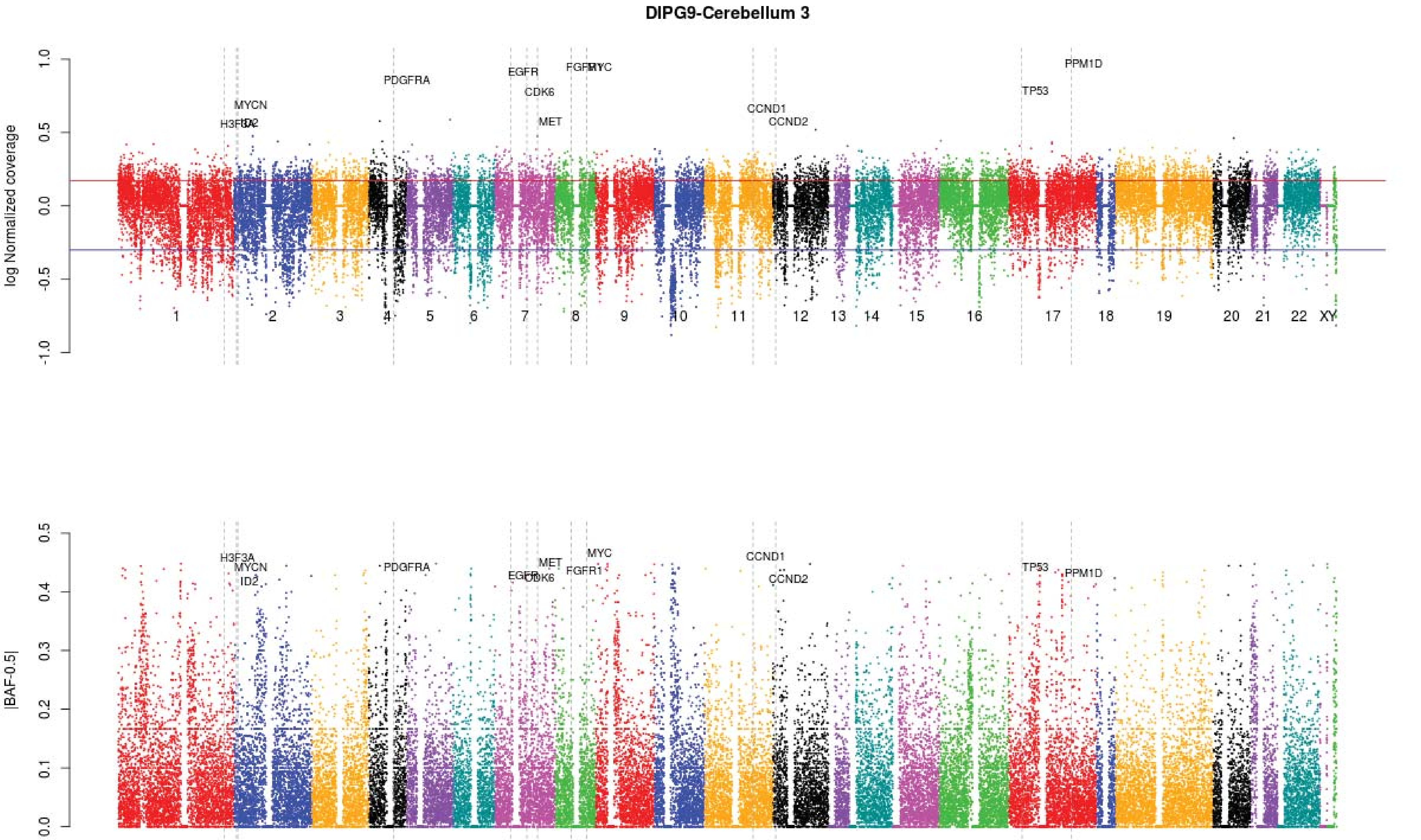
DIPG9-Cerebellum 3
log Normalized coverage
1.0
0.5
0.0
-0.5
-1.0
H3F3A
MYCN
ID2
PDGFRA
EGFR
CDK6
MET
FGFR1
MYC
CCND1
CCND2
TP53
PPM1D
1
2
3
4
5
6
7
8
9
10
11
12
13
14
15
16
17
18
19
20
21
22
XY
|BAF-0.5|
0.5
0.4
0.3
0.2
0.1
0.0

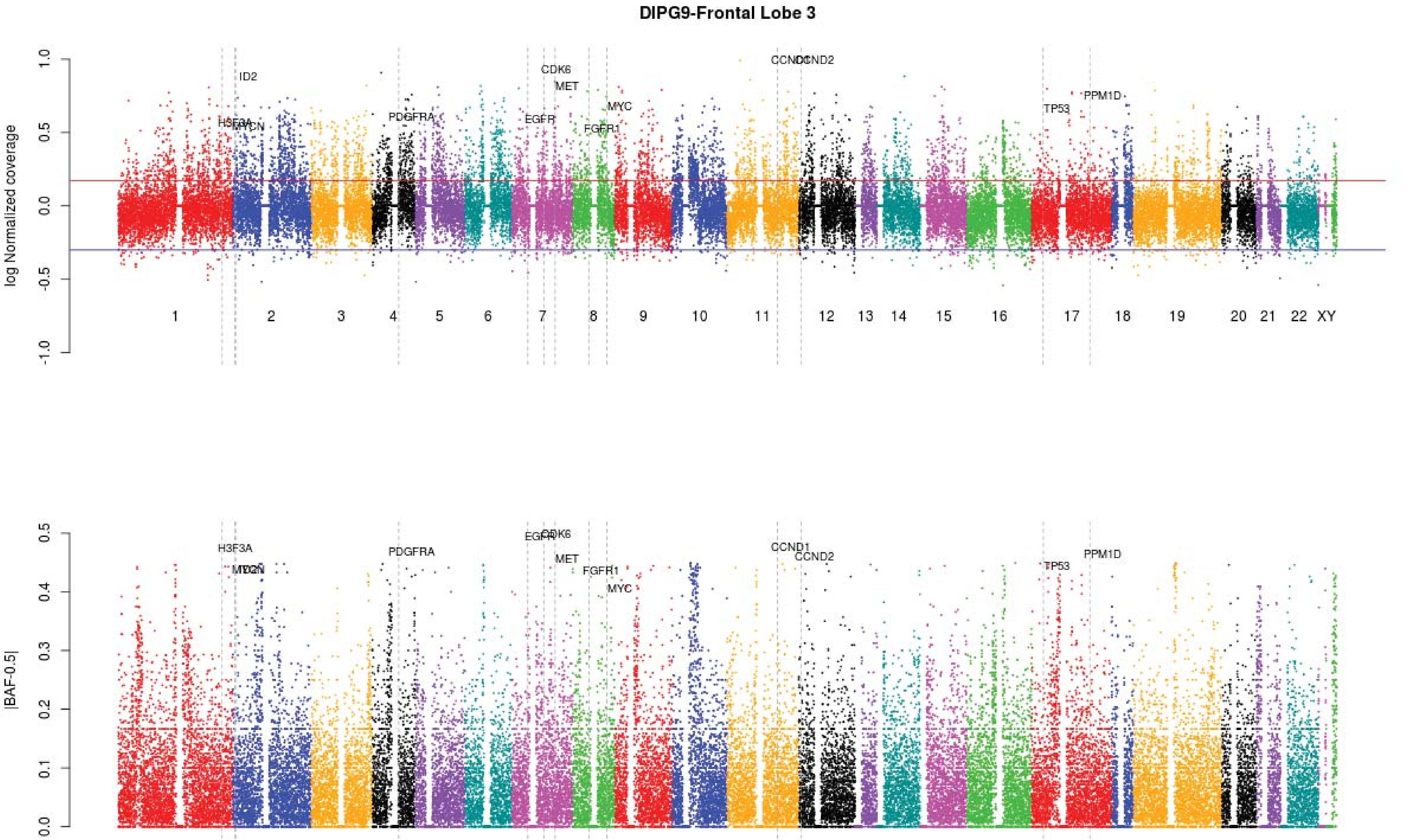

DIPG9-Frontal Lobe 3
log Normalized coverage
1.0
0.5
0.0
-0.5
-1.0
ID2
H3F3A
MYCN
PDGFRA
CDK6
MET
EGFR
MYC
FGFR1
CCND1
CCND2
TP53
PPM1D
1
2
3
4
5
6
7
8
9
10
11
12
13
14
15
16
17
18
19
20
21
22
XY
|BAF-0.5|
0.5
0.4
0.3
0.2
0.1
0.0

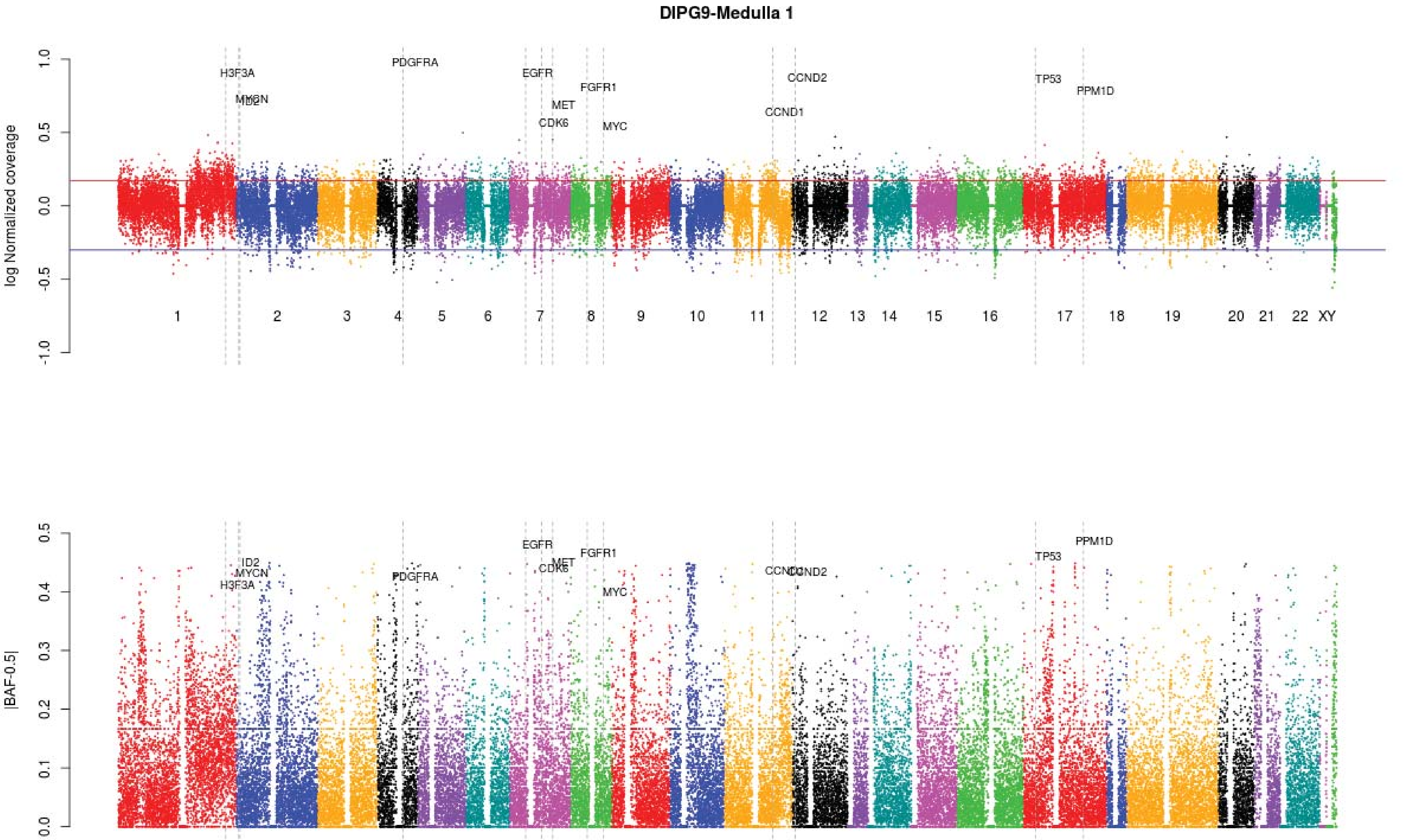
DIPG9-Medulla 1
log Normalized coverage
1.0
0.5
0.0
-0.5
-1.0
H3F3A
MYCN
ID2
PDGFRA
EGFR
MET
CDK6
FGFR1
MYC
CCND1
CCND2
TP53
PPM1D
1
2
3
4
5
6
7
8
9
10
11
12
13
14
15
16
17
18
19
20
21
22
XY
|BAF-0.5|
0.5
0.4
0.3
0.2
0.1
0.0

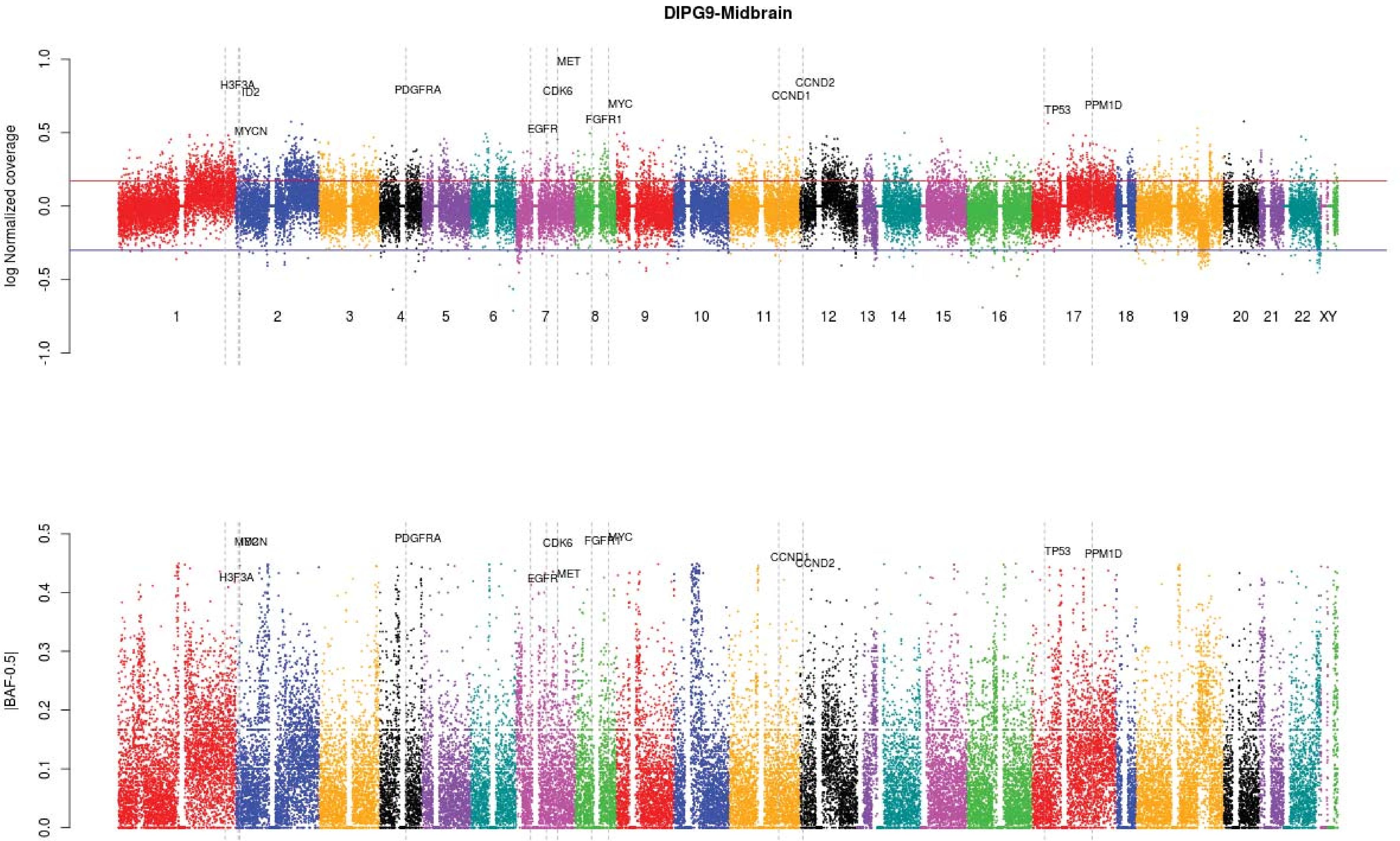
DIPG9-Midbrain
log Normalized coverage
1.0
0.5
0.0
-0.5
-1.0
H3F3A
ID2
MYCN
PDGFRA
EGFR
CDK6
MET
FGFR1
MYC
CCND1
CCND2
TP53
PPM1D
1
2
3
4
5
6
7
8
9
10
11
12
13
14
15
16
17
18
19
20
21
22
XY
|BAF-0.5|
0.5
0.4
0.3
0.2
0.1
0.0

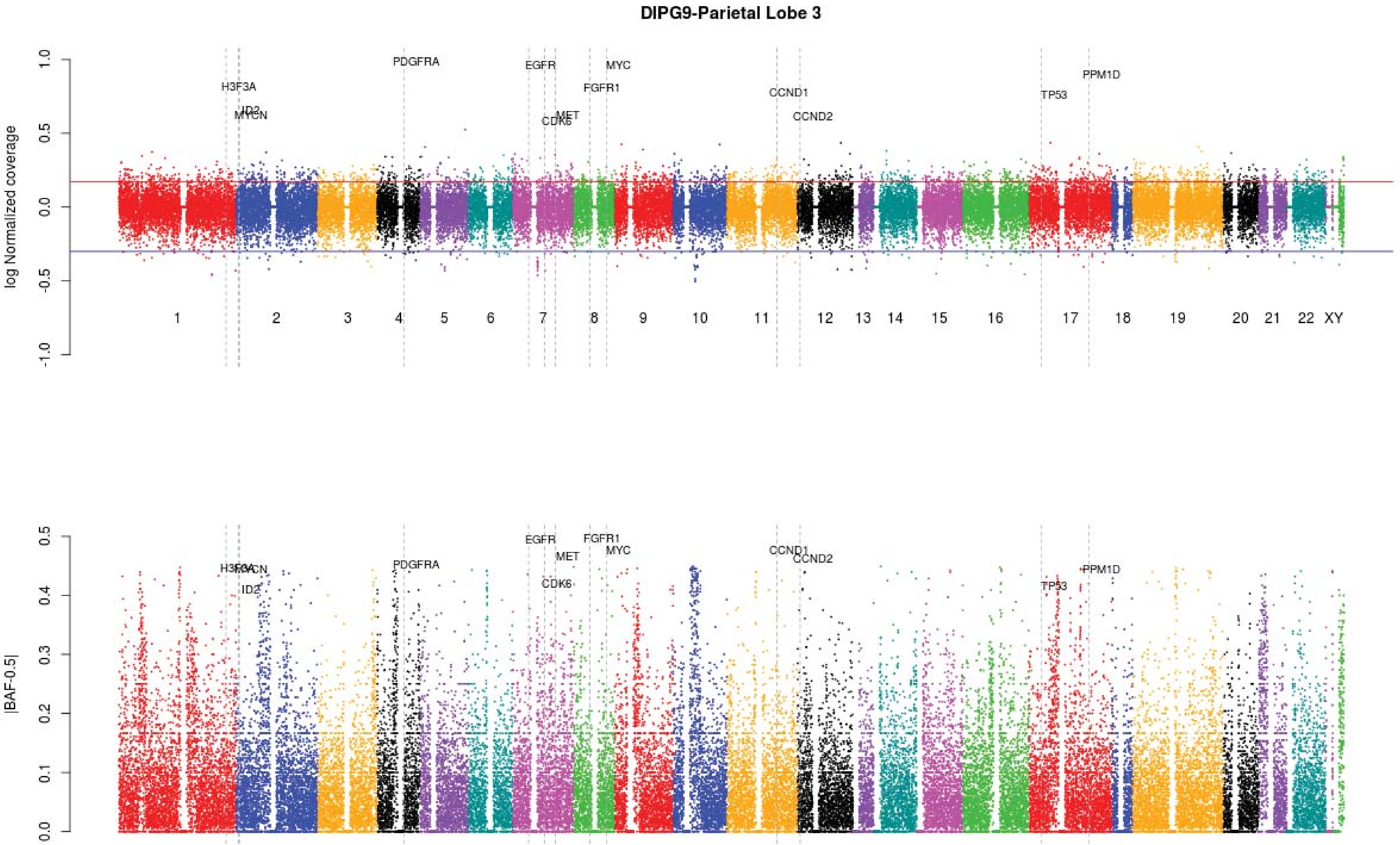

DIPG9-Parietal Lobe 3
log Normalized coverage
1.0
0.5
0.0
-0.5
-1.0
H3F3A
ID2
MYCN
PDGFRA
EGFR
CDK6
MET
FGFR1
MYC
CCND1
CCND2
TP53
PPM1D
1
2
3
4
5
6
7
8
9
10
11
12
13
14
15
16
17
18
19
20
21
22
XY
|BAF-0.5|
0.5
0.4
0.3
0.2
0.1
0.0

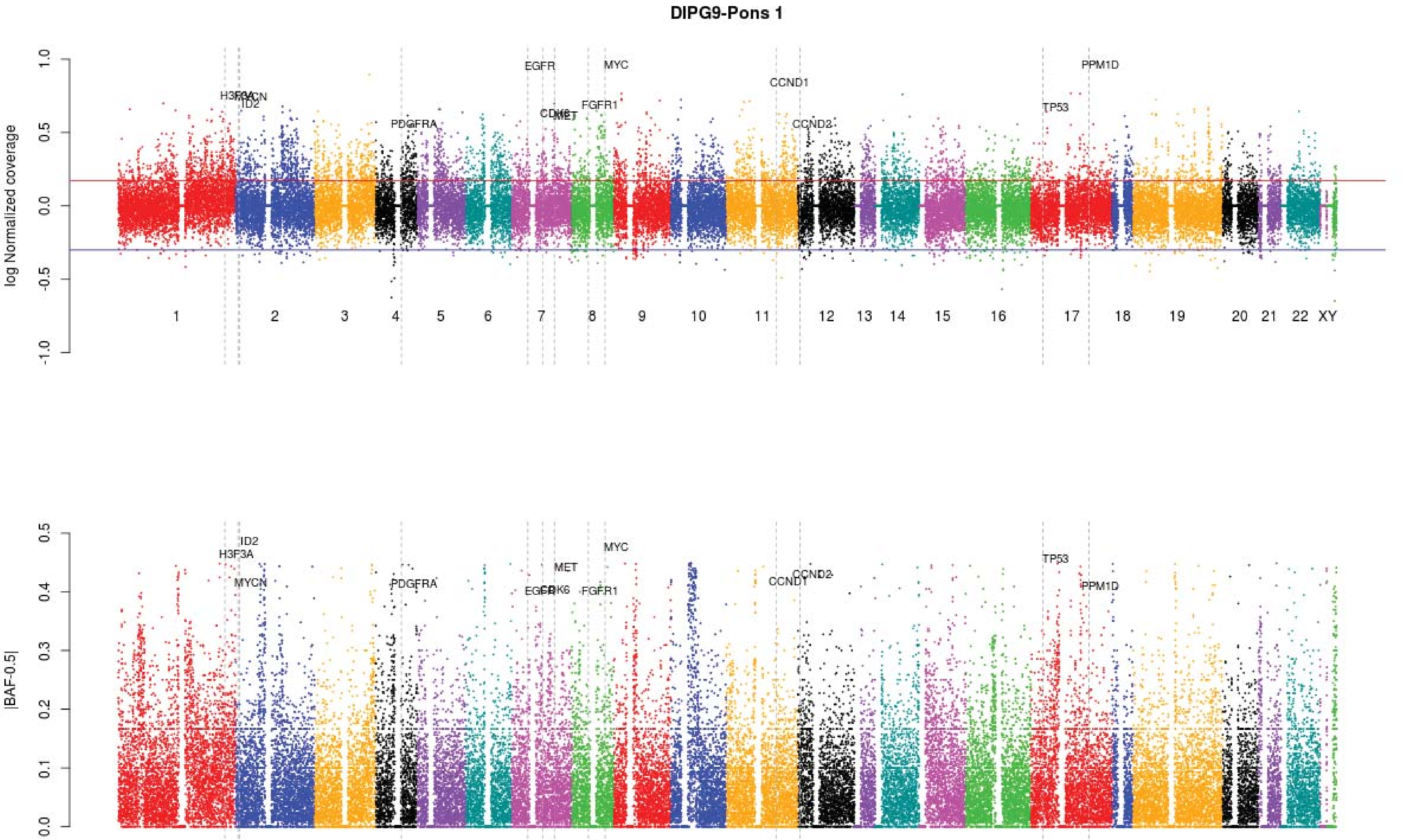

DIPG9-Pons 1
log Normalized coverage
|BAF-0.5|
H3F3A
MYCN
ID2
PDGFRA
EGFR
CDK6
MET
FGFR1
MYC
CCND1
CCND2
TP53
PPM1D
1
2
3
4
5
6
7
8
9
10
11
12
13
14
15
16
17
18
19
20
21
22
XY

DIPG9-Temporal Lobe 3

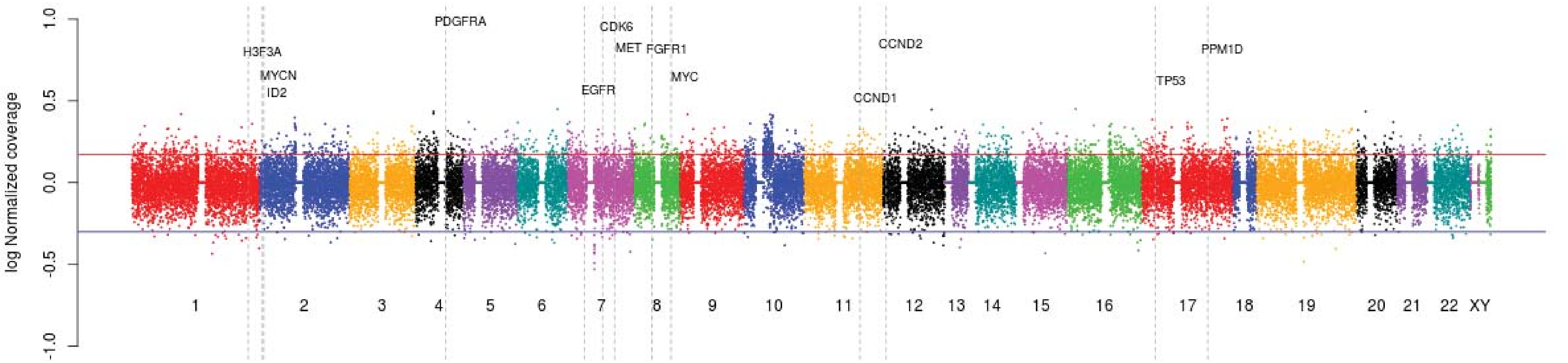

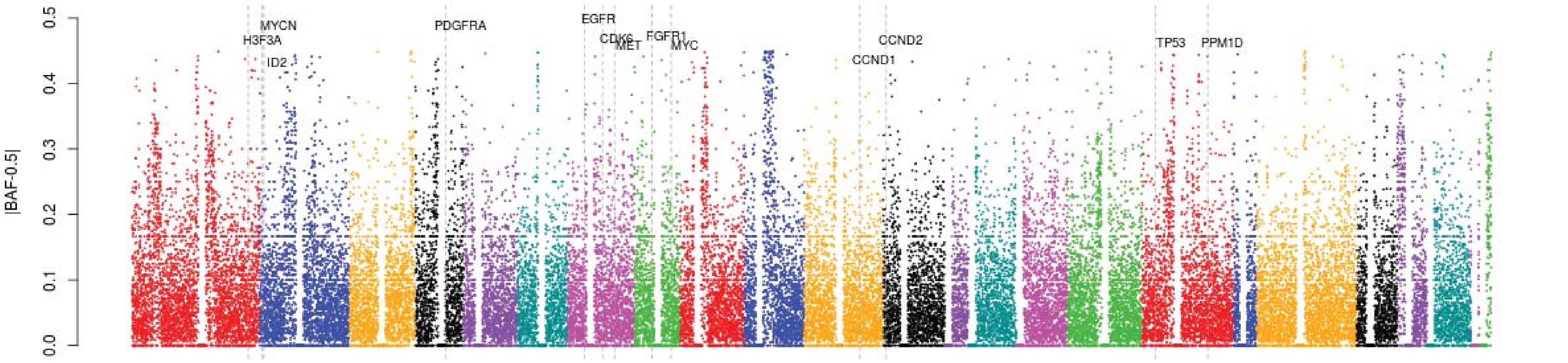

DIPG9-Ventricle

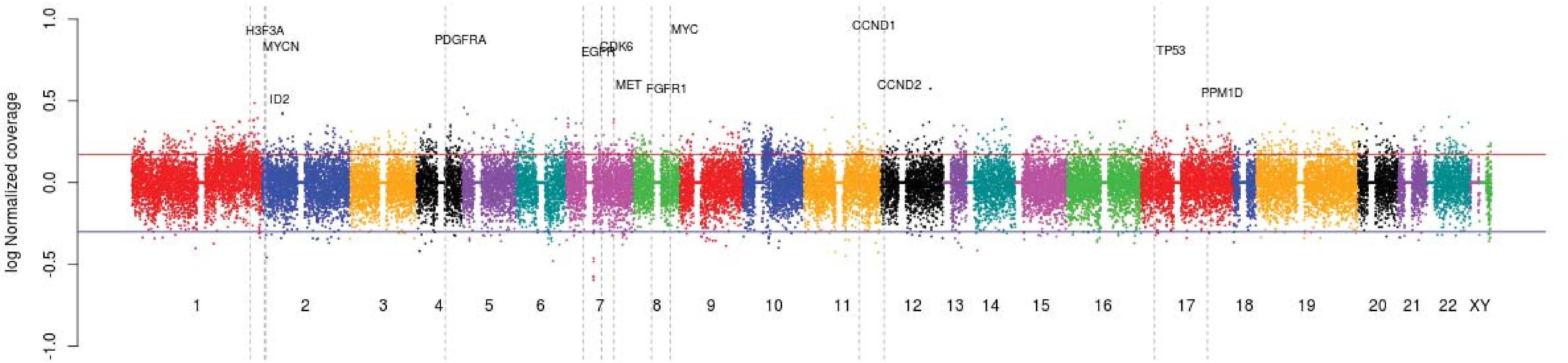

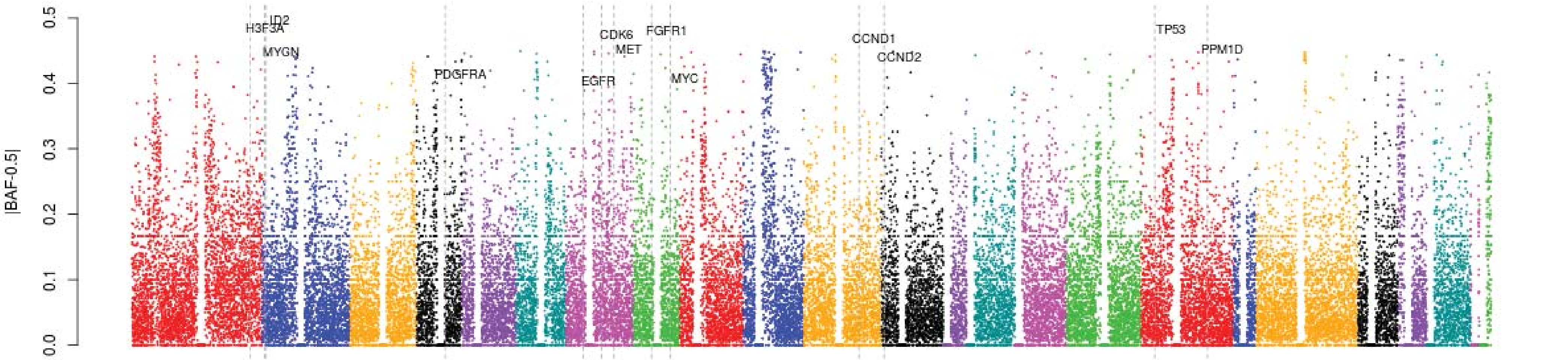

Supplementary Figure 5: CNV calling using whole exome sequencing data. Whole exome sequencing data was used to estimate copy number variation events in DIPG samples using deviation of B allele frequency from 50% as well as normalized coverage data (See online methods).

Supplementary Figure 6

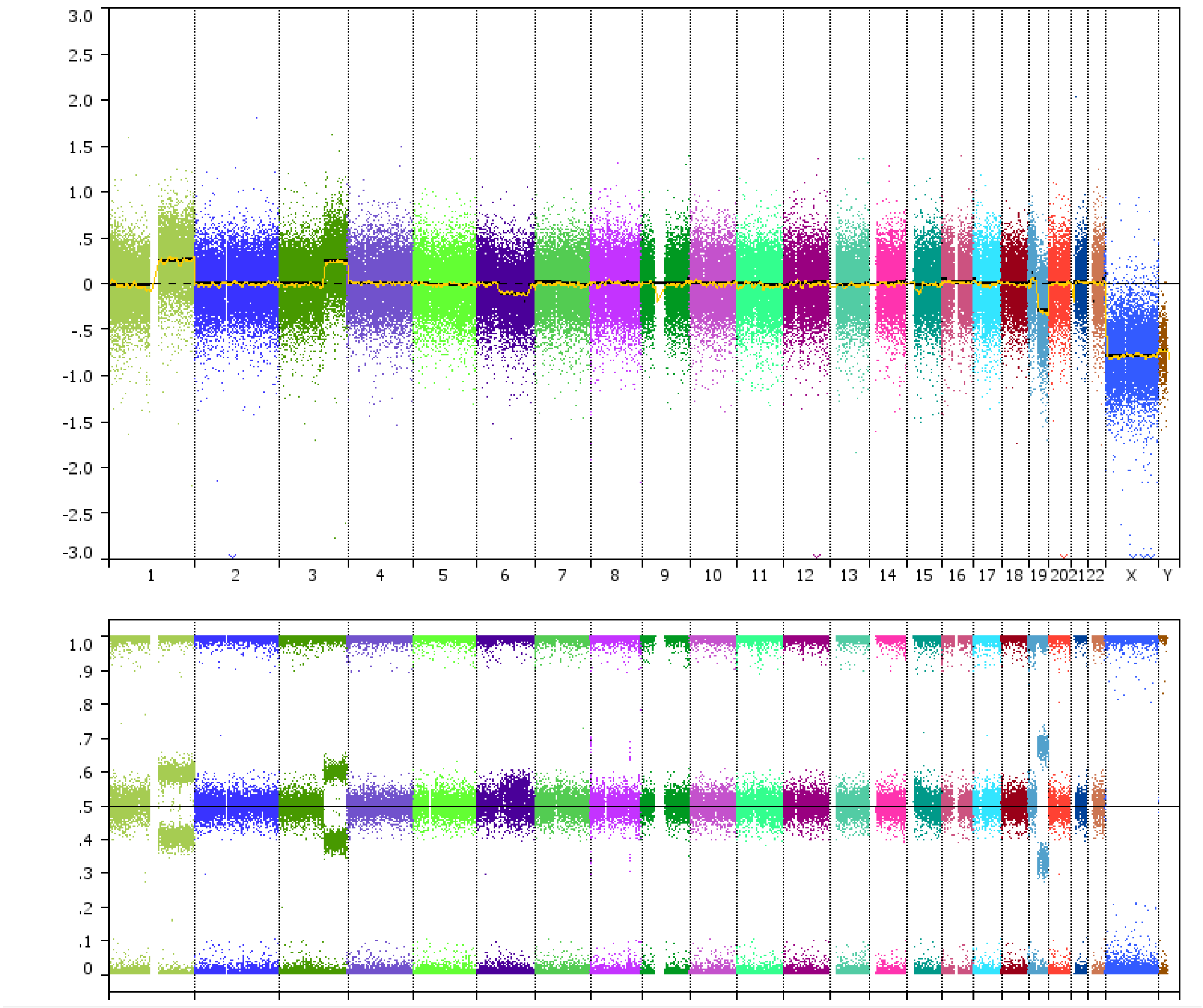

DIPG1-Pons1

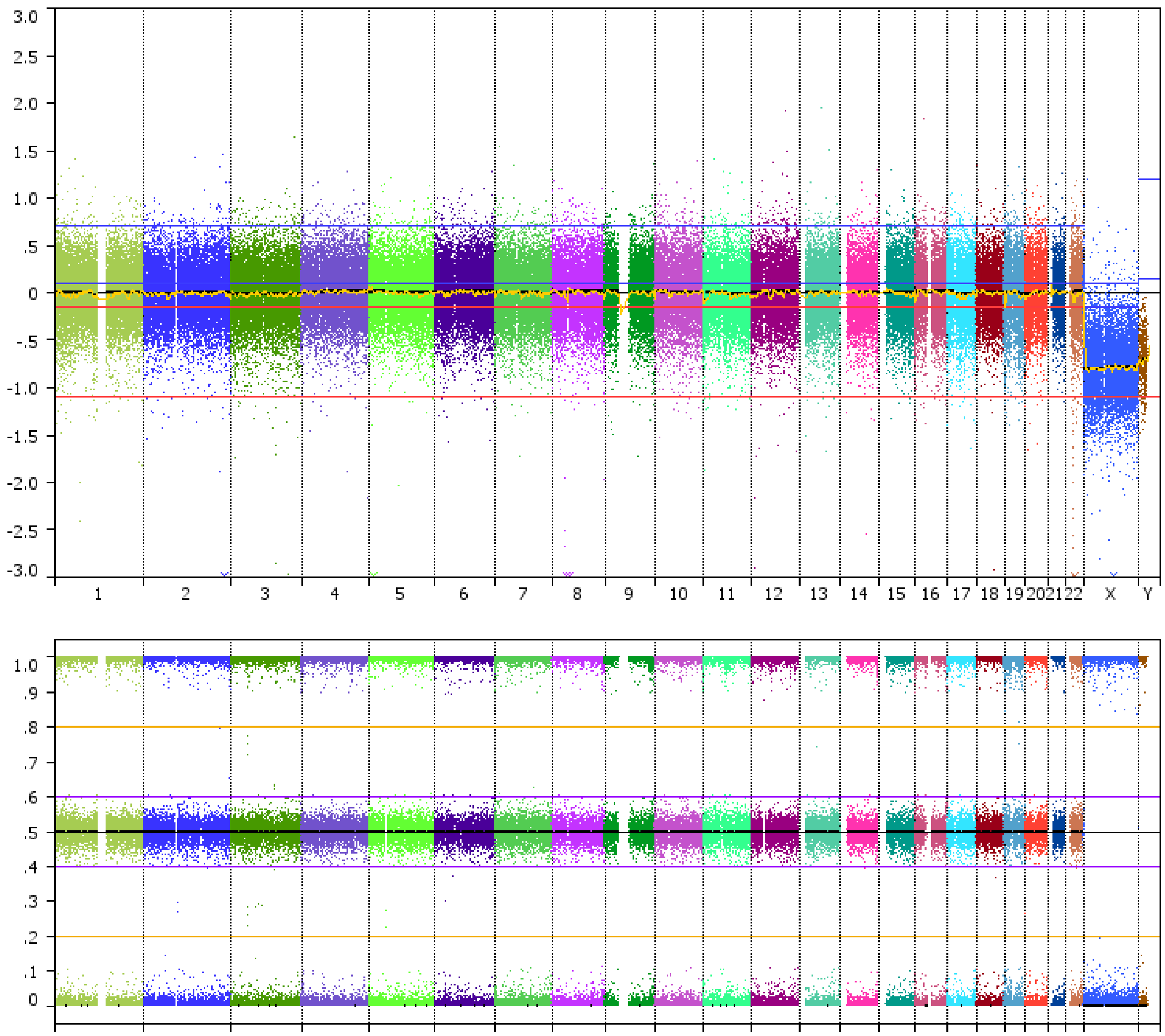

DIPG2-Cerebellum3

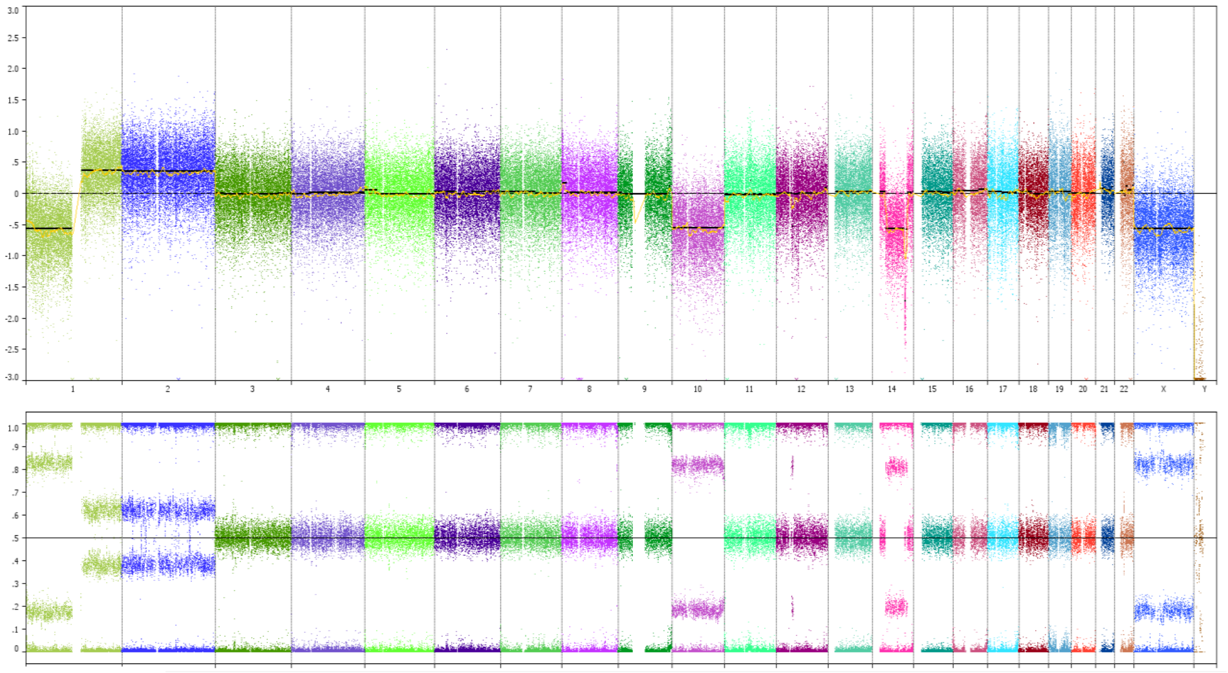

3.0
2.5
2.0
1.5
1.0
.5
0
-.5
-1.0
-1.5
-2.0
-2.5
-3.0
1
2
3
4
5
6
7
8
9
10
11
12
13
14
15
16
17
18
19
20
21
22
X
Y
1.0
.9
.8
.7
.6
.5
.4
.3
.2
.1
0

DIPG3-Pons2

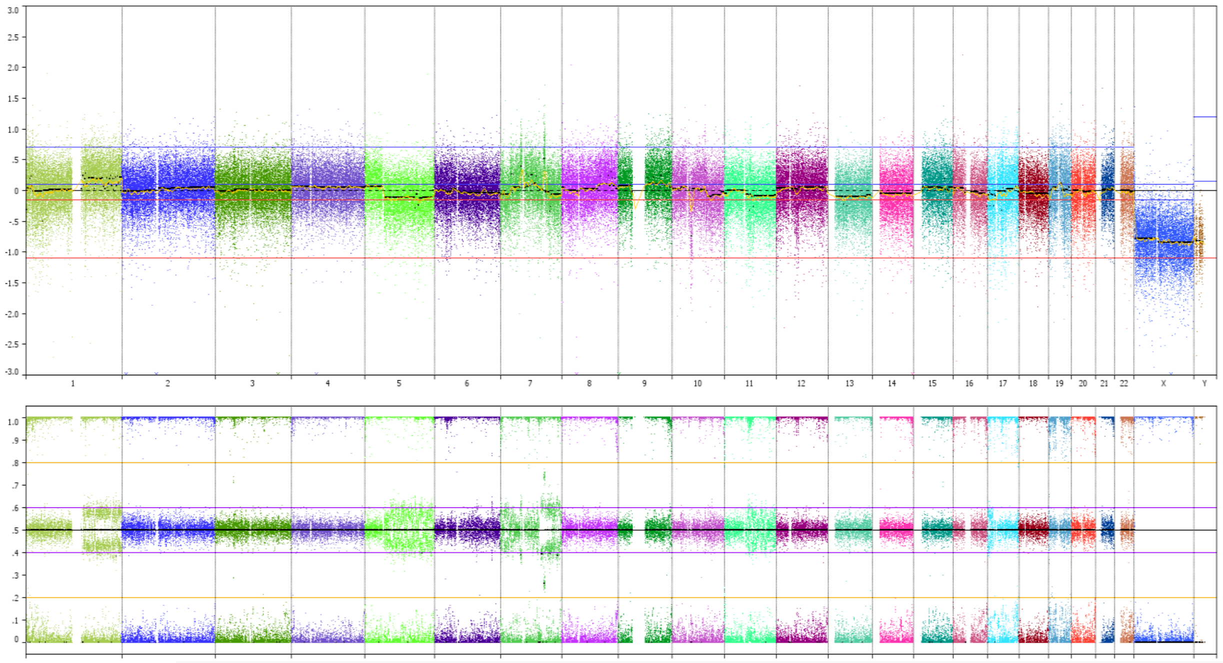
3.0
2.5
2.0
1.5
1.0
.5
0
-.5
-1.0
-1.5
-2.0
-2.5
-3.0
1
2
3
4
5
6
7
8
9
10
11
12
13
14
15
16
17
18
19
20
21
22
X
Y
1.0
.9
.8
.7
.6
.5
.4
.3
.2
.1
0

DIPG5-Pons1

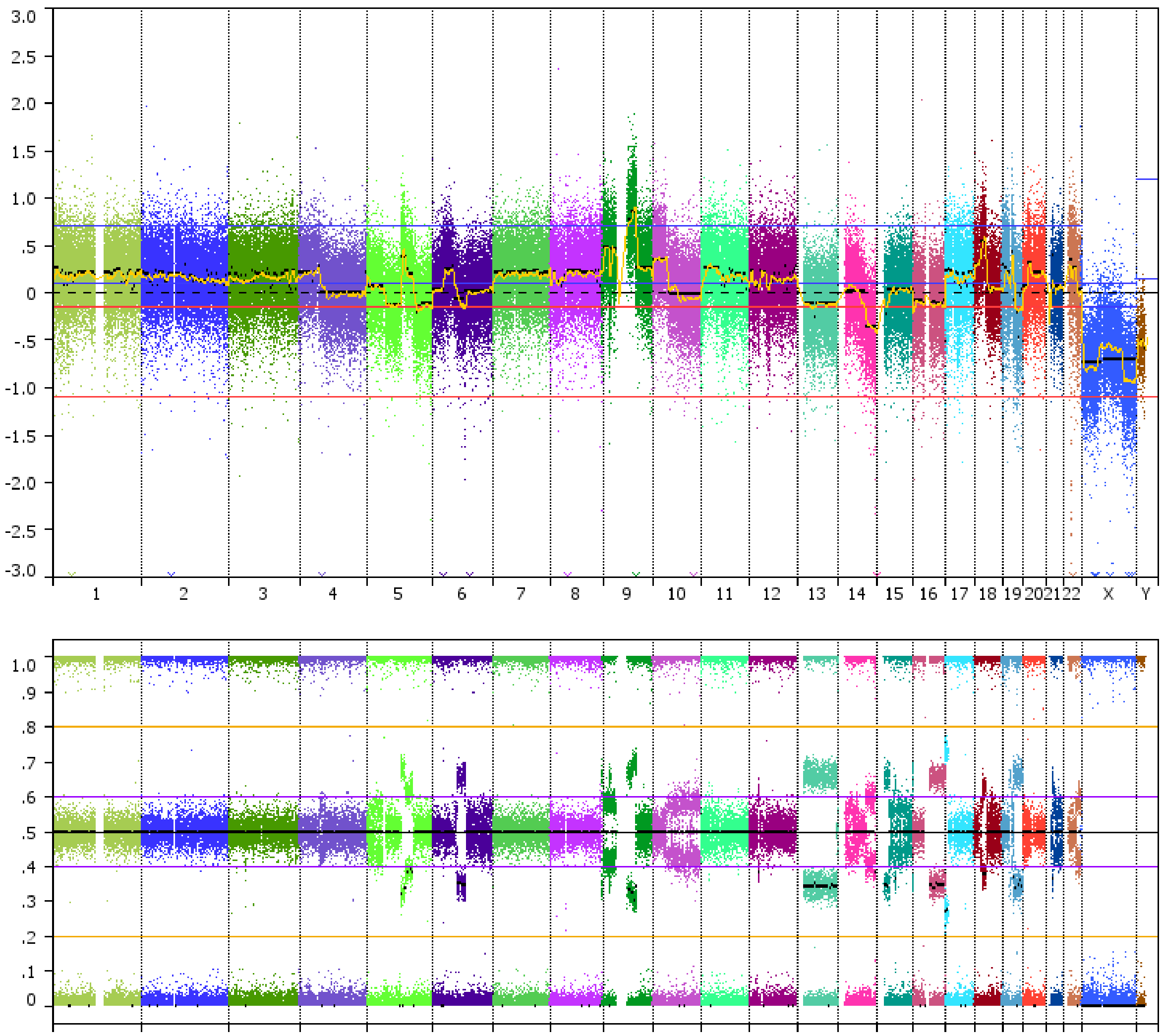
3.0
2.5
2.0
1.5
1.0
.5
0
-.5
-1.0
-1.5
-2.0
-2.5
-3.0
1
2
3
4
5
6
7
8
9
10
11
12
13
14
15
16
17
18
19
20
21
22
X
Y
1.0
.9
.8
.7
.6
.5
.4
.3
.2
.1
0

DIPG6-Pons5

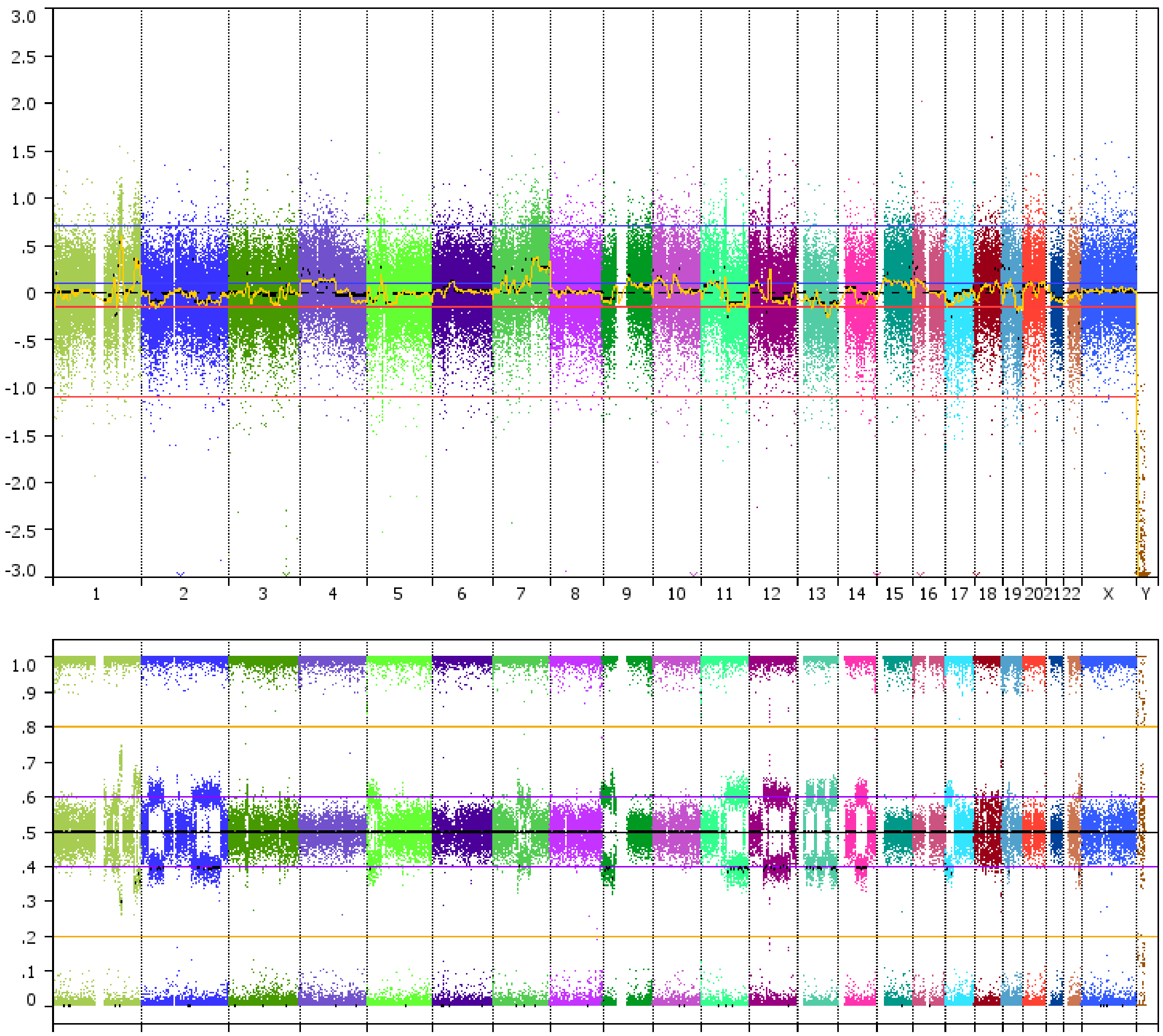

DIPG7-Cerebellum1

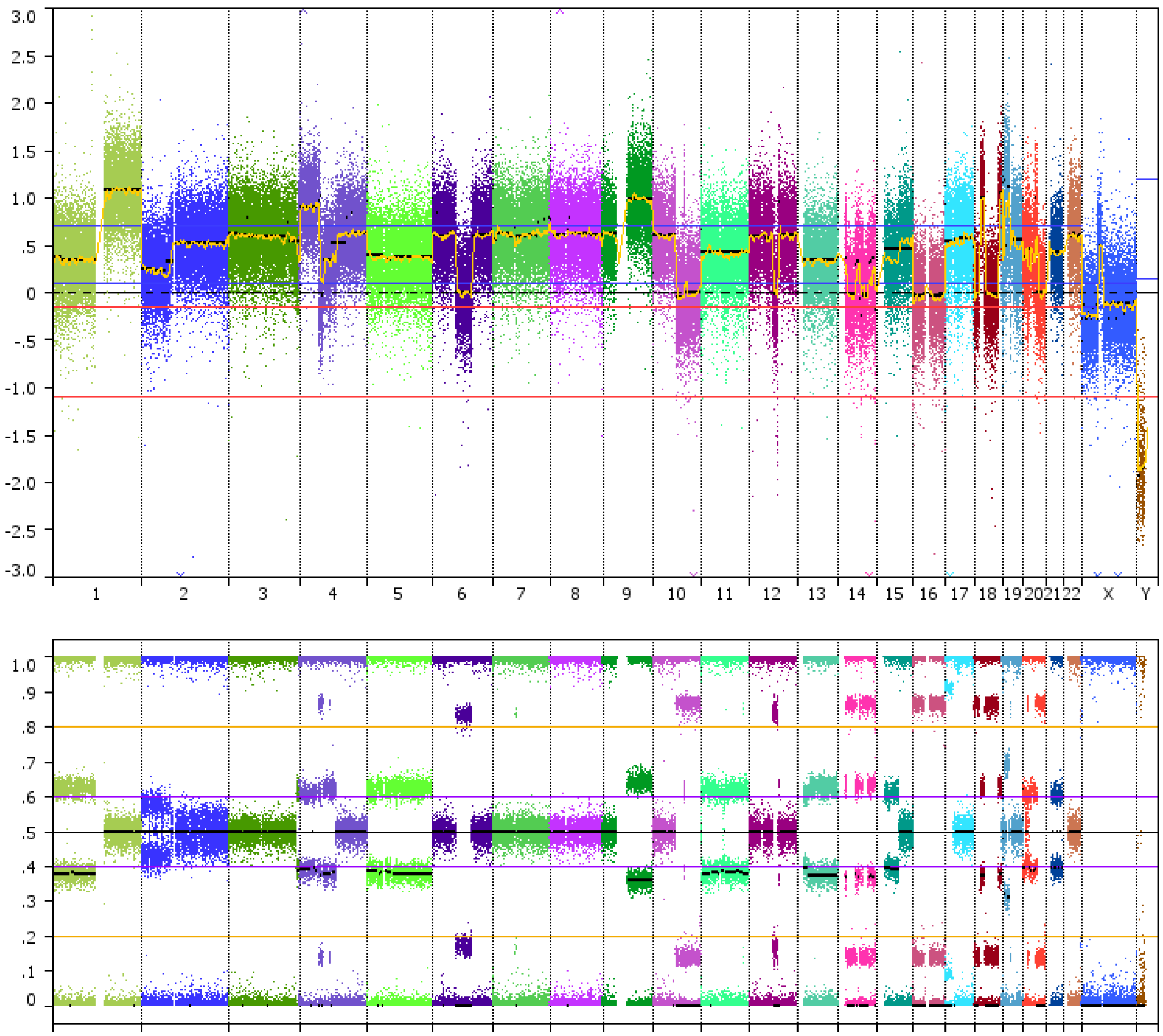

DIPG8-Midbrain

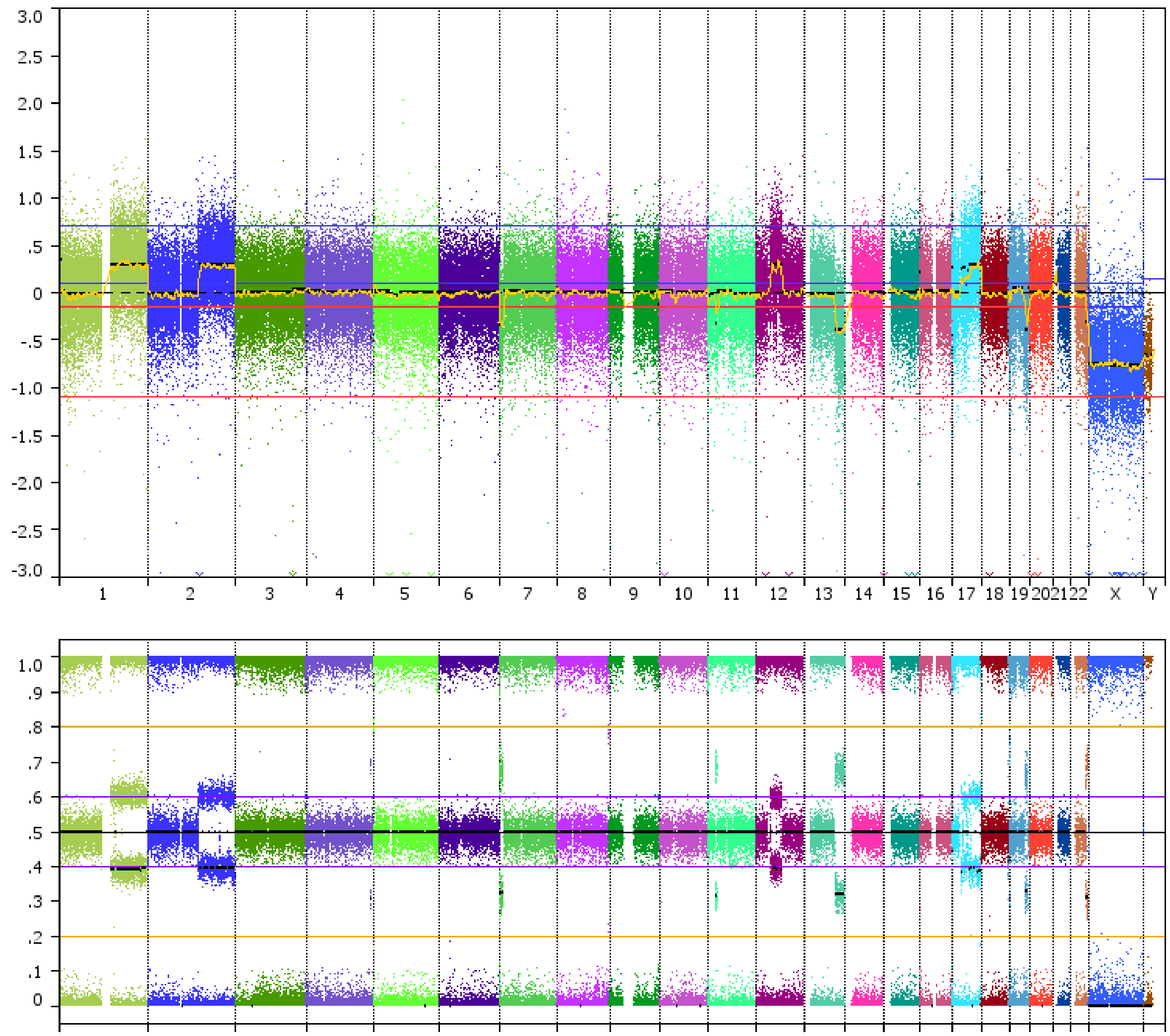

DIPG9-Midbrain

Supplementary Figure 6: OncoScan CNV arrays were used on selected samples to validate whole exome sequencing based CNV calling. Using the same DNA used for whole exome sequencing, one sample from each patient was selected to assess copy number variation events (except for patient DIPG4 for which no material was available). OncoSan results shown here correlated with whole exome based CNV calling.

Supplementary Figure 7

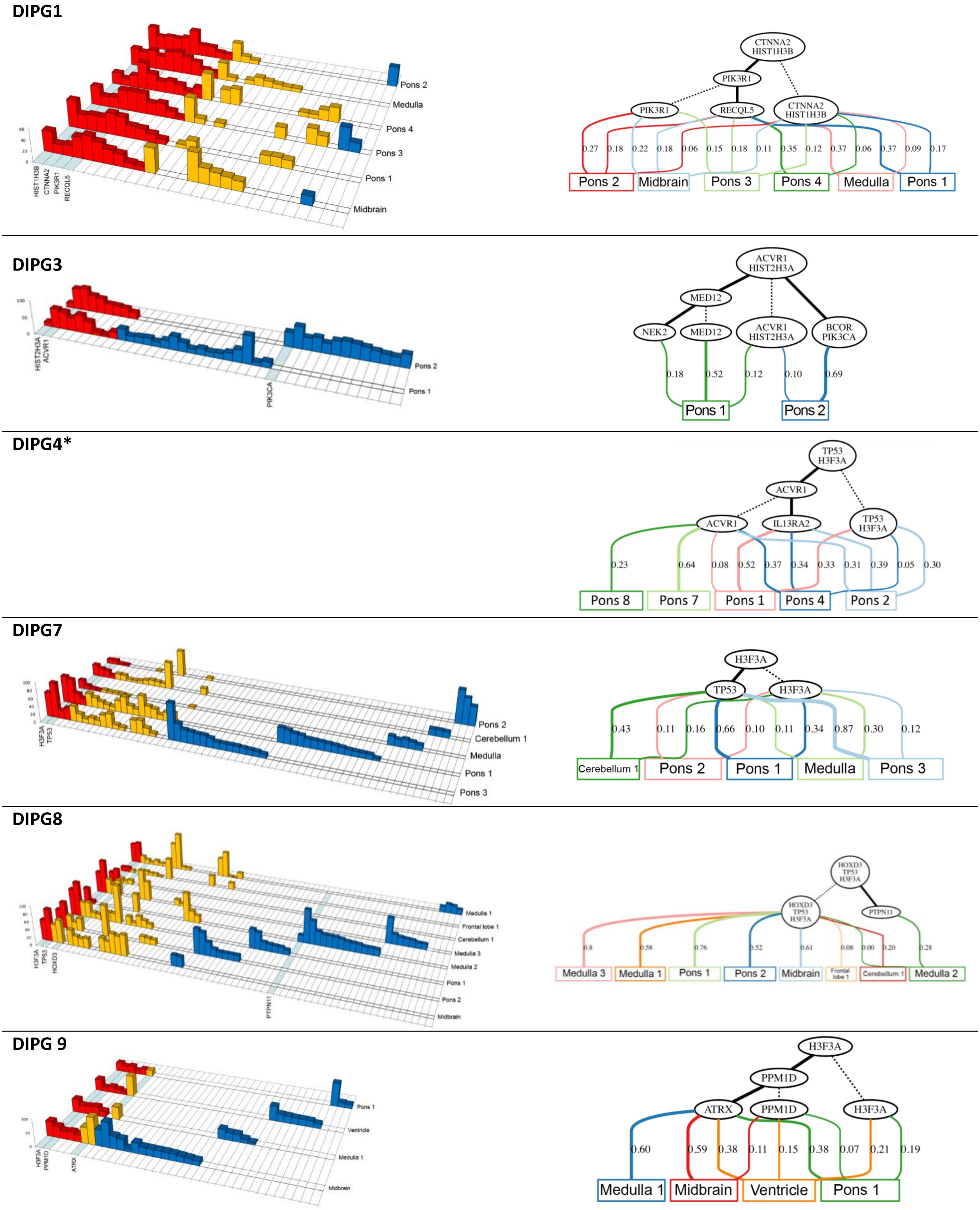

Supplementary Figure 7: Evolutionary reconstruction and non-synonymous somatic mutations allele frequency values for six patients (not included in Figure 2). Left: Histograms represent the raw allele frequency values from whole exome sequencing data (Supplementary table 2.) Right: Evolutionary trees reconstructed using CNV corrected allele frequencies from the deep amplicon sequencing data targeting the candidate genes found in whole exome sequencing.

*We had access to whole exome sequencing data from only one sample for patient DIPG4. The evolutionary tree for this patient was reconstructed based on deep amplicon sequencing data, except the frequency values were not corrected for CNV events (due to lack of whole exome sequencing data on the rest of the samples from this patient.) Lacking a robust correction for copy number, we consider this phylogeny less reliable and hence do not discuss it in the main text.

Supplementary Figure 8

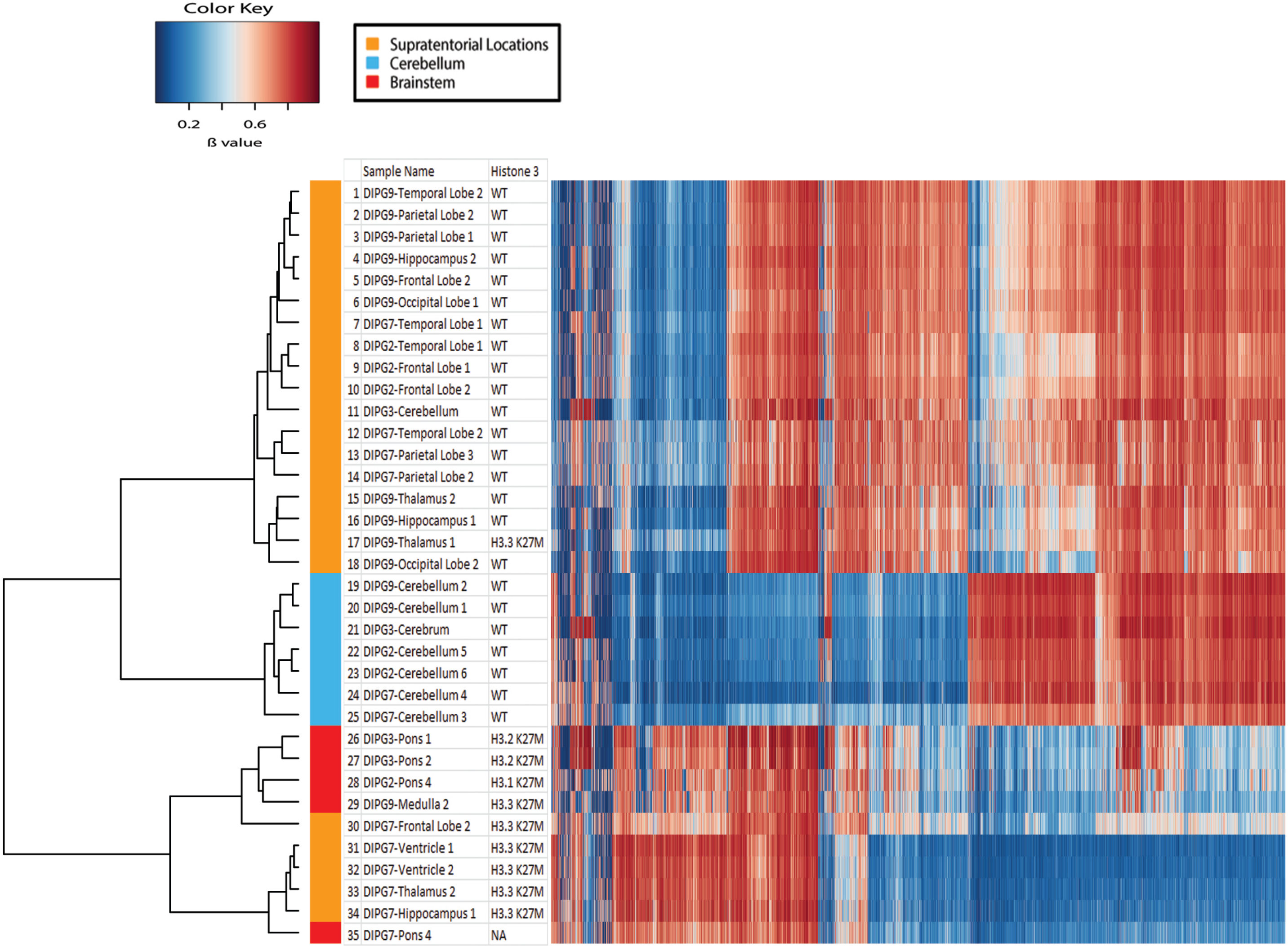

Supplementary Figure 8. Clustering analysis of global DNA methylation from various neuroanatomical locations for four DIPGs. Samples representing histone 3 K27M and wild type neuroanatomical locations for DIPG2 (H3.1-K27M), DIPG3 (H3.2-K27M), DIPG7 and DIPG9 (H3.3-K27M) were analyzed using unsupervised hierarchical clustering for the 5000 most variable probes. Global DNA methylation clustering demonstrates clustering patterns based on histone 3 mutation status, rather than neuroanatomical location. DIPG9 thalamus 1 is a H3-K27M sample clustering with the H3-WT group possible due to the presence of only few tumor cells only detectable by sensitive genomic screening and which based on their low content do not alter the global methylation profile.

Supplementary Figure 9

DIPG7

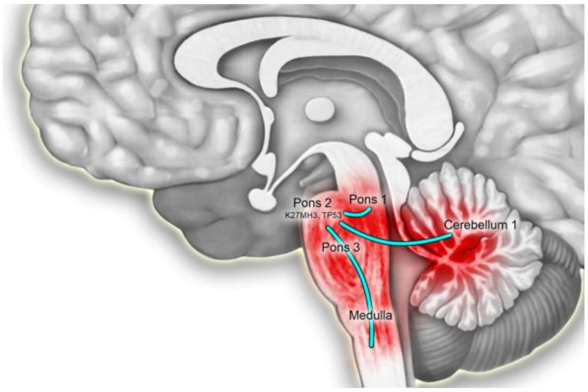

DIPG8

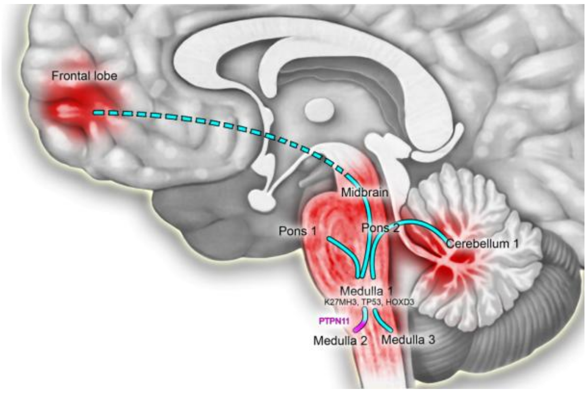

DIPG9

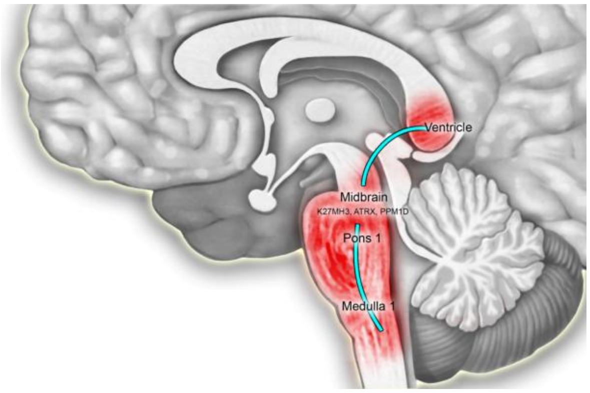

Supplementary Figure 9: Tumor spread outside the brain stem in patients DIPG7, DIPG8, and DIPG9. In DIPG7, spread to cerebellum and across the brain stem is consistent with early event in tumor evolution as it does not harbor additional mutations. In DIPG8, the anatomical spread path towards the frontal lobe is not known and hence is represented as a dashed line (--). Spread to “Medulla 2” in this patient is presumed to happen relatively later than the spread towards the cerebellum since it harbors an additional PTPN1 mutation. The spread towards the medulla and ventricle in DIPG9 is suggested to also happen relatively late in tumor evolution since both samples in these areas share the subclonal ATRX and PPM1D mutations seen in the primary tumor.

Supplementary Figure 10

DIPG	130
H3.1 K27M	29
H3.2 K27M	1
H3.3 K27M	86
H3 WT	14

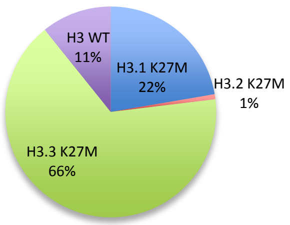

H3.1/2 K27M	30
ACVR1	9
ACVR1 + TP53	3
ACVR1 + PIK3CA	5
ACVR1 + PIK3CA + EGFR	1
ACVR1 + PIK3CA + PTEN	1
ACVR1 + PPM1D + PIK3CA	1
ACVR1 + PIK3R1	4
ACVR1 + PTEN	1
TP53	3
PIK3CA	1
PIK3R1	1

Pie Chart (H3.1/2)	30
ACVR1	9
ACVR1 + other mutations	16
TP53	3
PIK3CA	1
PIK3R1	1

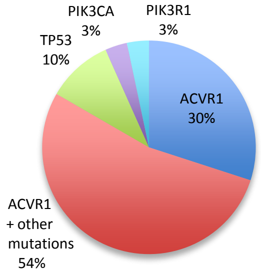

H3.3 K27M	86
ACVR1	1
ACVR1 + PIK3CA	2
TP53 + ACVR1	2
TP53	50
TP53 + PPM1D	1
TP53 + PIK3CA	5
TP53 + PIK3R1	2
TP53 + PDGFRA	5
PPM1D	3
PPM1D + PIK3CA	3
PPM1D + PIK3CA + ACVR1	2
PPM1D + PIK3R1	1
PIK3R1	2
FGFR1	1
CHEK2	1
H3.3 K27M only	5

Pie Chart (H3.3 K27M)	86
ACVR1	1
ACVR1 + other mutations	2
TP53	50
TP53 + other mutations	15
PPM1D	3
PPM1D + other mutations	6
PIK3R1	2
FGFR1	1
CHEK2	1
H3.3 K27M only	5

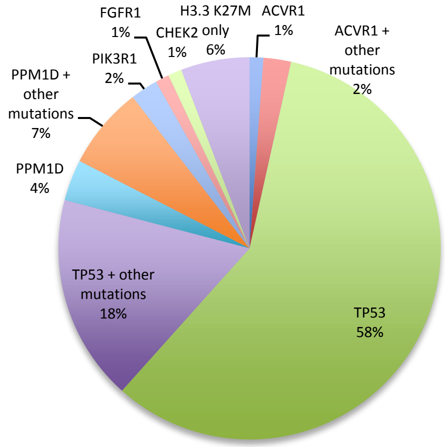

H3 WT	14
ACVR1	1
ACVR1 + PIK3CA + PIK3R1	1
ACVR1 + PIK3CA + PPM1D	2
TP53	6
WT	4

Pie chart (H3 WT)	14
ACVR1	1
ACVR1 + other mutations	3
TP53	6
WT	4

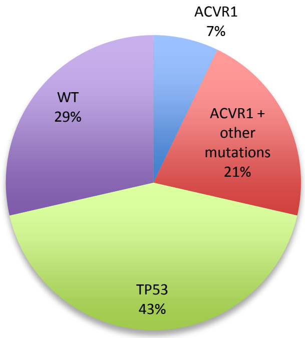

Supplementary Figure 10: Summary of integrated dataset of Diffuse Intrinsic Pontine Glioma samples from four published studies (n=121) and present work (n=9) showing distribution of histone 3 mutation status as well as mutation combinations of oncohistone partners for all patients. For a detailed list, please refer to Supplmentary Table 15.

Supplementary Table 1: Clinical and Molecular Data from 134 Punch Cores Taken From Autopsy Brains of 9 DIPG patients

PATIENT INFORMATION									MOLECULAR ANALYSES										IMMUNOHISTOCHEMISTRY				
				Treatment						MISEQ RUN 1 (2014)		MISEQ RUN 2 (2015)					Whole Exome Sequencing		Stains				
Patient ID	Diagnosis	Gender	Age	Chemotherapy	Radiation	Sample Name	Neuroanatomical Location	Tissue Type	ddPCR Run1	Histone 3	Other Genes	Histone 3	Other Genes	Methylation	RNA-Seq	ddPCR Run2	Histone 3	Other Genes	H&E	Ki67	H3 K27M	H3 K27me3	Comments
DIPG1 (H3.1)	DIPG (GBM)	Male	21y 2m	SAHA, Avastin, and Irinotecan	Yes	DIPG1-Frontal Lobe 1	B	Frozen		WT					1 core punch	WT	WT	no mutations					cerebral cortex & cerebellum is FFPE
						DIPG1-Frontal Lobe 2	B	FFPE											√		√	√	
						DIPG1-Cerebellum 1	E	FFPE											√	√	√	√	
						DIPG1-Cerebellum 2	E	FFPE				WT				H3.1B K27M (4.6%)	failed	failed	√	√	√	√	
						DIPG1-Ventricle 1	F	FFPE											√	√	√	√	
						DIPG1-Ventricle 2	F	FFPE											√	√	√	√	
						DIPG1-Hippocampus	G	FFPE											√	√	√	√	
						DIPG1-Thalamus	H	FFPE											√	√	√	√	
						DIPG1-Midbrain	I	Frozen				H3.1B K27M (19.32%)	PIK3R1 splicing (24.85%), CTNNA2 A260S (25.33%), RECQL5 splicing (17.24%)		1 Core Punch	H3.1B K27M (25%)	H3.1 K27M	CTNNA2 A260S; PIK3R1 splicing					
						DIPG1-Pons 1	J	Frozen		H3.1 K27M (52%)		H3.1B K27M (24.4%)	PIK3R1 splicing (23.75%), CTNNA2 A260S (27.39%), RECQL5 splicing (21.99%)		1 Core Punch	H3.1B K27M (34%)	H3.1 K27M	CTNNA2 A260S; PIK3R1 splicing					
						DIPG1-Pons 2	J	Frozen				H3.1B K27M (29%)	PIK3R1 splicing (29.33%), CTNNA2 A260S (33.04%), RECQL5 splicing (18.02%)				H3.1 K27M	CTNNA2 A260S; PIK3R1 splicing					
						DIPG1-Pons 3	J	Frozen				H3.1B K27M (23.1%)	PIK3R1 splicing (21.02%), CTNNA2 A260S (22.55%), RECQL5 splicing (16.85%)		1 Core Punch	H3.1B K27M (23%)	H3.1 K27M	CTNNA2 A260S; PIK3R1 splicing					
						DIPG1-Pons 4	J	Frozen		H3.1 K27M (19%)		H3.1B K27M (18.56%)	PIK3R1 splicing (21.97%), CTNNA2 A260S (20.6%), RECQL5 splicing (21.14%)		1 Core Punch	H3.1B K27M (23%)	H3.1 K27M	CTNNA2 A260S; PIK3R1 splicing					
						DIPG1-Pons 5	J	FFPE											√	√	√	√	
						DIPG1-Pons 6	J	FFPE											√	√	√	√	
						DIPG1-Medulla	K	Frozen		H3.1 K27M (24%)		H3.1B K27M (25.43%)	PIK3R1 splicing (25.60%), CTNNA2 A260S (24.21%), RECQL5 splicing (18.95%)		1 Core Punch	H3.1B K27M (30%)	H3.1 K27M	CTNNA2 A260S; PIK3R1 splicing					
						DIPG2-Temporal Lobe 1	A	FFPE		WT				Does not cluster with K27M group					√	√	√	√	
						DIPG2-Temporal Lobe 2	A	FFPE	WT														
						DIPG2-Temporal Lobe 3	A	Frozen				WT				WT	WT	no mutations					
						DIPG2-Temporal Lobe 4	A	FFPE	WT														
						DIPG2-Frontal Lobe 1	B	FFPE	WT					Does not cluster with K27M group									
						DIPG2-Frontal Lobe 2	B	FFPE	WT					Does not cluster with K27M group					√	√	√	√	
						DIPG2-Frontal Lobe 3	B	Frozen		WT		WT				WT	WT	no mutations					
						DIPG2-Parietal Lobe 1	C	FFPE	WT														
						DIPG2-Parietal Lobe 2	C	FFPE	WT										√	√	√	√	
						DIPG2-Parietal Lobe 3	C	FFPE	WT														
						DIPG2-Parietal Lobe 4	C	Frozen				WT				WT	WT	no mutations					

						DIPG2-Parietal Lobe 5	C	Frozen				WT				WT	WT	no mutations					
						DIPG2-Occipital Lobe 1	D	FFPE	WT														
						DIPG2-Occipital Lobe 2	D	Frozen				WT				WT	WT	no mutations					
**DIPG2 (H3.1)**	**DIPG (GBM)**	**Male**	**6y 10m**	**N/A**	**Yes**	DIPG2-Cerebellum 1	E	Frozen				H3.1B K27M (15.49%)	PIK3CA H1047R (0.65%), ACVR1 G328V (23.69%), MAX R51Q (0.62%), SETD5 K527Q (0.77%)			H3.1B K27M (16%)	H3.1 K27M	ACVR1 G328V					
						DIPG2-Cerebellum 2	E	Frozen				WT				failed	WT	no mutations					
						DIPG2-Cerebellum 3	E	Frozen				WT				WT	WT	no mutations					
						DIPG2-Cerebellum 4	E	Frozen				WT				WT	WT	no mutations					
						DIPG2-Cerebellum 5	E	FFPE		WT				Does not cluster with K27M group					√	√	√	√	
						DIPG2-Cerebellum 6	E	FFPE		WT				Does not cluster with K27M group					√	√	√	√	
						DIPG2-Thalamus	H	Frozen		H3.1 K27M (49%)	ACVR1 G328V (70%)	H3.1B K27M (45.03%)	PIK3CA H1047R (23.94%), ACVR1 G328V (60.72%), MAX R51Q (5.87%), PTEN A126S (16.34%), SETD5 K527Q (0.34%)			H3.1B K27M (45%)	H3.1 K27M	ACVR1 G328V; PIK3CA H1047R; MAX R51Q; PTEN A126S					
						DIPG2-Pons 1	J	Frozen		H3.1 K27M (37%)	ACVR1 G328V (57%)	H3.1B K27M (33.44%)	PIK3CA H1047R (0.36%), ACVR1 G328V (57.73%), MAX R51Q (16.27%), PTEN A126S (1.49%), SETD5 K527Q (0.62%)			H3.1B K27M (41%)	H3.1 K27M	ACVR1 G328V; MAX R51Q					
						DIPG2-Pons 2	J	Frozen				H3.1B K27M (37.14%)	PIK3CA H1047R (42.47%), ACVR1 G328V (58.13%), MAX R51Q (0.68%), ASH1L D286fs (24.84%), SETD5 K527Q (44.08%)			H3.1B K27M (30%)	H3.1 K27M	ACVR1 G328V; PIK3CA H1047R					
						DIPG2-Pons 3	J	Frozen				H3.1B K27M (33.22%)	PIK3CA H1047R (0.66%), ACVR1 G328V (55.21%), MAX R51Q (11.09%), PTEN A126S (0.28%), SETD5 K527Q (0.37%)		1 Core Punch	H3.1B K27M (39%)	H3.1 K27M	ACVR1 G328V; MAX R51Q;					
						DIPG2-Pons 4	J	FFPE		H3.1 K27M (49%)	ACVR1 G328V (70%)			Clusters with K27M group					√	√	√	√	
						DIPG2-Pons 5	J	FFPE											√	√	√	√	
						DIPG2-Pons 6	J	FFPE											√	√	√	√	
						DIPG2-Medulla	K	Frozen				H3.1B K27M (20.05%)	PIK3CA H1047R (0.78%), ACVR1 G328V (40.68%), MAX R51Q (6.94%), SETD5 K527Q (0.49%)		1 Core Punch	H3.1B K27M (26%)	H3.1 K27M	ACVR1 G328V; MAX R51Q;					
						DIPG3-Cerebrum	N/A	Frozen		WT				Does not cluster with K27M group	1 Core Punch		WT	no mutations					
**DIPG3 (H3.2)**	**DIPG (GBM)**	**Female**	**7y 5m**	**No**	**Yes**	DIPG3-Cerebellum	E	Frozen		WT				Does not cluster with K27M group	1 Core Punch		WT	no mutations					
						DIPG3-Pons 1	J	Frozen		H3.2 K27M (8%)	ACVR1 G328V (47%)			Clusters with K27M group	1 Core Punch		H3.2 K27M	ACVR1 G328V					
						DIPG3-Pons 2	J	Frozen		H3.2 K27M (5%)	ACVR1 G328V (57%)			Clusters with K27M group	1 Core Punch		H3.2 K27M	ACVR1 G328V; PIK3CA H1047R					
						DIPG4-Frontal Lobe 1	B	Frozen		WT						WT	WT	no mutations					
						DIPG4-Frontal Lobe 2	B	FFPE											√	√	√	√	
						DIPG4-Cerebellum	E	FFPE											√		√	√	
						DIPG4-Pons 1	J	Frozen				H3.3 K27M (46.47%)	ACVR1 R206H (29.01%), TP53 C3F (57.84%), IL13RA2 Y12D (28.91%)			H3.3 K27M (44%)	H3.3 K27M	TP53 C3F; ACVR1 R206H					
						DIPG4-Pons 2	J	Frozen		H3.3 K27M (45%)	ACVR1 R206H (30%)	H3.3 K27M (50.29%)	ACVR1 R206H (34.01%), TP53 C3F (61.84%), IL13RA2 Y12D (19.94%)										
**DIPG4 (H3.3)**	**DIPG (GBM)**	**Female**	**5y 6m**	**Capecitabine**	**Yes**	DIPG4-Pons 3	J	FFPE											√	√	√	√	cerebral cortex & cerebellum

						DIPG4-Pons 4	J	Frozen				H3.3 K27M (36.98%)	ACVR1 R206H (36.35%), TP53 C3F (60.57%), IL13RA2 Y12D (18.67%)		1 Core Punch								cerebellum is FFPE
						DIPG4-Pons 5	J	FFPE											√		√	√	
						DIPG4-Pons 6	J	Frozen							2 Core Punches								
						DIPG4-Pons 7	J	Frozen				H3.3 K27M (29.61%)	ACVR1 R206H (49.88%), TP53 C3F (60.96%), IL13RA2 Y12D (0.59%)										
						DIPG4-Pons 8	J	Frozen				H3.3 K27M (12.78%)	ACVR1 R206H (23.3%), TP53 C3F (42.57%), IL13RA2 Y12D (1.75%)										
						DIPG4-Pons 9	J	FFPE											√	√	√	√	
						DIPG4-Pons 10	J	Frozen															
DIPG5 (H3.3)	DIPG (GBM)	Male	8y	N/A	N/A	DIPG5-Frontal Lobe	B	FFPE											√	√	√	√	cerebral cortex & cerebellum is FFPE
						DIPG5-Cerebellum 1	E	FFPE	WT										√		√	√	
						DIPG5-Cerebellum 2	E	FFPE		WT		WT				WT	failed	failed	√		√	√	
						DIPG5-Midbrain 1	I	Frozen		H3.3 K27M (76%)		H3.3 K27M (57.06%)	PPM1D W427X (43.52%), MAP3K15 Y368fs (17.65%), TNIK R364S (1.65%), PPP1CA E231K (36.25%)		1 Core Punch	H3.3 K27M (58%)	H3.3 K27M	PPM1D W427X					
						DIPG5-Midbrain 2	I	Frozen		WT		WT				WT	WT	no mutations					
						DIPG5-Midbrain 3	I	FFPE											√		√	√	
						DIPG5-Pons 1	J	Frozen		H3.3 K27M (47%)		H3.3 K27M (42.23%)	ATRX D94fs (37.84%), TP53 A175fs (8.08%), PPM1D W427X (13.8%), TNIK R364S (52.98%)		2 Core Punches	H3.3 K27M (44%)	H3.3 K27M	ATRX D94fs; TP53 A175fs; PPM1D W427X					
						DIPG5-Pons 2	J	FFPE											√		√	√	
						DIPG5-Medulla	K	Frozen		H3.3 K27M (44%)		H3.3 K27M (48.79%)	TP53 A175fs (48.75%), PPM1D W427X (4.09%), TNIK R364S (30.23%)		1 Core Punch	H3.3 K27M (44%)	H3.3 K27M	TP53 A175fs;					
DIPG6 (H3.3)	DIPG (Astrocytoma)	Male	6y	Temozolomide	Yes	DIPG6-Frontal Lobe 1	B	Frozen		WT						WT	WT	no mutations					cerebral cortex & cerebellum is FFPE
						DIPG6-Frontal Lobe 2	B	FFPE											√		√	√	
						DIPG6-Cerebellum	E	FFPE	WT										√		√	√	
						DIPG6-Thalamus	H	FFPE											√	√	√	√	
						DIPG6-Midbrain	I	Frozen				H3.3 K27M (6.19%)	ATRX splicing (11.21%), PIK3CA H1047R (0.45%), TP53 G11D (11%), OLIG2 P215fs (4.44%)			H3.3 K27M (3%)	H3.3 K27M	PIK3CA H1047R; TP53 G113D; OLIG2 P215fs					
						DIPG6-Pons 1	J	Frozen		H3.3 K27M (46%)		H3.3 K27M (57.17%)	ATRX splicing (88.65%), PIK3CA H1047R (85.40%), TP53 G11D (85.40%), OLIG2 P215fs (34.46%)			H3.3 K27M (39%)	H3.3 K27M	TP53 G113D; OLIG2 P215fs					
						DIPG6-Pons 2	J	Frozen				H3.3 K27M (50.43%)	ATRX splicing (84.57%), PIK3CA H1047R (5.71%), TP53 G11D (86.03%), OLIG2 P215fs (46.3%)				H3.3 K27M	TP53 G113D; OLIG2 P215fs					
						DIPG6-Pons 3	J	Frozen				H3.3 K27M (46.4%)	ATRX splicing (92.7%), PIK3CA H1047R (30.41%), TP53 G11D (92.61%), OLIG2 P215fs (50.13%)			H3.3 K27M (31%)	H3.3 K27M	TP53 G113D; ATRX splicing; PIK3CA H1047R; OLIG2 P215fs					
						DIPG6-Pons 4	J	Frozen				H3.3 K27M (42.61%)	ATRX splicing (85.96%), PIK3CA H1047R (2.52%), TP53 G11D (86.24%), OLIG2 P215fs (44%)			H3.3 K27M (22%)	H3.3 K27M	ATRX splicing; TP53 G113D; OLIG2 P215fs					
						DIPG6-Pons 5	J	Frozen				H3.3 K27M (9.2%)	ATRX splicing (49.57%), PIK3CA H1047R (3.06%), TP53 G11D (51.19%), OLIG2 P215fs (23.5%)			H3.3 K27M (23%)	H3.3 K27M	TP53 G113D; OLIG2 P215fs					
						DIPG6-Pons 6	J	FFPE	WT										√	√	√	√	
						DIPG7-Temporal Lobe 1	A	FFPE		WT		WT		Does not cluster with K27M group		WT	failed	failed	√		√	√	
						DIPG7-Temporal Lobe 2	A	FFPE		WT				Does not cluster with K27M group					√		√	√	
						DIPG7-Frontal Lobe 1	B	FFPE		WT									√		√	√	

DIPG7 (H3.3)	DIPG (GBM)	Female	10y 8m	SAHA	Yes	DIPG7-Frontal Lobe 2	B	FFPE		H3.3 K27M (26%)				Clusters with K27M group					√	√	√	√	cerebral cortex & cerebellum is FFPE
						DIPG7-Parietal Lobe 1	C	Frozen		WT		WT			1 core punch	WT	WT	no mutations					
						DIPG7-Parietal Lobe 2	C	FFPE	WT					Does not cluster with K27M group					√		√	√	
						DIPG7-Parietal Lobe 3	C	FFPE	WT					Does not cluster with K27M group					√		√	√	
						DIPG7-Occipital Lobe 1	D	FFPE		WT									√		√	√	
						DIPG7-Occipital Lobe 2	D	FFPE		WT									√		√	√	
						DIPG7-Cerebellum 1	E	Frozen				H3.3 K27M (28.5%)	TP53 R43H (20.12%), PBRM1 splicing (0.35%)			H3.3 K27M (26%)	H3.3 K27M	TP53 R43H					
						DIPG7-Cerebellum 2	E	Frozen								WT	WT	no mutations					
						DIPG7-Cerebellum 3	E	FFPE		WT				Does not cluster with K27M group					√	√	√	√	
						DIPG7-Cerebellum 4	E	FFPE				WT		Does not cluster with K27M group					√	√	√	√	
						DIPG7-Ventricle 1	F	FFPE		H3.3 K27M (64%)				Clusters with K27M group					√	√	√	√	
						DIPG7-Ventricle 2	F	FFPE		H3.3 K27M (48%)				Clusters with K27M group					√	√	√	√	
						DIPG7-Hippocampus 1	G	FFPE	H3.3 K27M (59%)					Clusters with K27M group					√	√	√	√	
						DIPG7-Hippocampus 2	G	FFPE		WT									√		√	√	
						DIPG7-Thalamus 1	H	FFPE		WT									√		√	√	
						DIPG7-Thalamus 2	H	FFPE		H3.3 K27M (52%)				Clusters with K27M group					√	√	√	√	
						DIPG7-Pons 1	J	Frozen		H3.3 K27M (26%)		H3.3 K27M (66.75%)	TP53 R43H (67.55%), PBRM1 splicing (0.23%)			H3.3 K27M (64%)	H3.3 K27M	TP53 R43H					
						DIPG7-Pons 2	J	Frozen				H3.3 K27M (9.74%)	TP53 R43H (6.88%), PBRM1 splicing (0.22%)			H3.3 K27M (10%)	H3.3 K27M	TP53 R43H					
						DIPG7-Pons 3	J	Frozen				H3.3 K27M (56.87%)	TP53 R43H (87.04%), PBRM1 splicing (0.51%)		2 Core Punches	H3.3 K27M (58%)	H3.3 K27M	TP53 R43H					
						DIPG7-Pons 4	J	FFPE		N/A				Clusters with K27M group					√	√	√	√	
						DIPG7-Pons 5	J	Frozen				WT			2 Core Punches	WT	WT	no mutations					
						DIPG7-Pons 6	J	FFPE											√	√	√	√	
						DIPG7-Medulla	K	Frozen				H3.3 K27M (20.87%)	TP53 R43H (6.57%), PBRM1 splicing (0.36%)			H3.3 K27M (21%)	H3.3 K27M	TP53 R43H					
						DIPG8-Temporal Lobe 1	A	FFPE	WT														
						DIPG8-Temporal Lobe 2	A	FFPE	WT														
						DIPG8-Frontal Lobe 1	B	Frozen		WT		H3.3 K27M (5.11%)	TP53 E162X (11.6%), HOXD3 H431Q (4.48%), PTPN11 A72V (0.09%)			H3.3 K27M (9%)	H3.3 K27M	TP53 E162X					
						DIPG8-Frontal Lobe 2	B	FFPE	WT										√	√	√	√	
						DIPG8-Frontal Lobe 3	B	FFPE	WT										√	√	√	√	
						DIPG8-Parietal Lobe 1	C	FFPE	WT														
						DIPG8-Parietal Lobe 2	C	Frozen				WT				WT	WT	no mutations					
						DIPG8-Occipital Lobe	D	Frozen				WT				H3.3 K27M (21%)	WT	TP53 (3.13%)					

DIPG8 (H3.3)	DIPG (GBM)	Male	5y	Temozolomide, Avastin, SAHA	Yes	DIPG8-Cerebellum 1	E	Frozen				H3.3 K27M (17.96%)	TP53 E162X (22.17%), HOXD3 H431Q (4.27%), PTPN11 A72V (0.08%)			H3.3 K27M (24%)	H3.3 K27M	TP53 E162X					
						DIPG8-Cerebellum 2	E	Frozen				WT				WT	WT	TP53 (2.03%)					
						DIPG8-Cerebellum 3	E	FFPE	H3.3 K27M (6%)										√	√	√	√	
						DIPG8-Cerebellum 4	E	FFPE	H3.3 K27M (8%)										√	√	√	√	
						DIPG8-Ventricle	F	Frozen				WT				WT	WT	no mutations					
						DIPG8-Hippocampus 1	G	FFPE	WT			WT				WT	failed	failed					
						DIPG8-Hippocampus 2	G	FFPE	WT														
						DIPG8-Thalamus 1	H	FFPE	WT										√	√	√	√	
						DIPG8-Thalamus 2	H	FFPE	H3.3 K27M (4%)										√	√	√	√	
						DIPG8-Midbrain	I	Frozen				H3.3 K27M (41.66%)	TP53 E162X (77.78%), HOXD3 H431Q (46.62%), PTPN11 A72V (0.09%)			H3.3 K27M (16%)	H3.3 K27M	TP53 E162X					
						DIPG8-Pons 1	J	Frozen				H3.3 K27M (62.5%)	TP53 E162X (91.58%), HOXD3 H431Q (49.78%), PTPN11 A72V (0.01%)			H3.3 K27M (71%)	H3.3 K27M	TP53 E162X					
						DIPG8-Pons 2	J	Frozen				H3.3 K27M (44.42%)	TP53 E162X (69.52%), HOXD3 H431Q (25.18%), PTPN11 A72V (0.14%)			H3.3 K27M (21%)	H3.3 K27M	TP53 E162X					
						DIPG8-Pons 3	J	FFPE	H3.3 K27M (12%)										√	√	√	√	
						DIPG8-Pons 4	J	FFPE	H3.3 K27M (17%)										√	√	√	√	
						DIPG8-Pons 5	J	FFPE	H3.3 K27M (13%)										√	√	√	√	
						DIPG8-Medulla 1	K	Frozen		H3.3 K27M (48%)		H3.3 K27M (47.33%)	TP53 E162X (58.07%), HOXD3 H431Q (28.14%), PTPN11 A72V (0.33%)			H3.3 K27M (48%)	H3.3 K27M	TP53 E162X					
						DIPG8-Medulla 2	K	Frozen				H3.3 K27M (30.03%)	TP53 E162X (10.27%), HOXD3 H431Q (7.25%), PTPN11 A72V (17.68%)			H3.3 K27M (34%)	H3.3 K27M	TP53 E162X					
						DIPG8-Medulla 3	K	Frozen				H3.3 K27M (55.43%)	TP53 E162X (85.62%), HOXD3 H431Q (39.88%), PTPN11 A72V (0.15%)			H3.3 K27M (57%)	H3.3 K27M	TP53 E162X					
						DIPG9-Temporal Lobe 1	A	FFPE	WT														
						DIPG9-Temporal Lobe 2	A	FFPE	WT					Does not cluster with K27M group									
						DIPG9-Temporal Lobe 3	A	Frozen								WT	WT	no mutations					
						DIPG9-Frontal Lobe 1	B	FFPE	WT										√	√	√	√	
						DIPG9-Frontal Lobe 2	B	FFPE	WT					Does not cluster with K27M group									
						DIPG9-Frontal Lobe 3	B	Frozen		WT		WT				WT	WT	no mutations					
						DIPG9-Parietal Lobe 1	C	FFPE	WT					Does not cluster with K27M group									
						DIPG9-Parietal Lobe 2	C	FFPE	WT					Does not cluster with K27M group									
						DIPG9-Parietal Lobe 3	C	Frozen				WT				WT	WT	no mutations					
						DIPG9-Occipital Lobe 1	D	FFPE	WT					Does not cluster with K27M group									
				ABT888 [illegible]		DIPG9-Occipital Lobe 2	D	FFPE	WT					Does not cluster with K27M group									

**DIPG9 (H3.3)**	**DIPG (GBM)**	**Male**	**9y 3m**	**(Veliparb) Temozolomide in maintenance phase**	**Yes**	DIPG9-Occipital Lobe 3	D	Frozen				WT				WT	WT	no mutations				
						DIPG9-Cerebellum 1	E	FFPE		WT				Does not cluster with K27M group					√	√	√	√
						DIPG9-Cerebellum 2	E	FFPE		WT				Does not cluster with K27M group					√	√	√	√
						DIPG9-Cerebellum 3	E	Frozen				WT			1 Core Punch	failed	WT	no mutations				
						DIPG9-Ventricle	F	Frozen				H3.3 K27M (36.49%)	ATRX V1514D (37.84%), PPM1D E525X (24.68%)			H3.3 K27M (32%)	H3.3 K27M	PPM1D E525X; ATRX V1514D				
						DIPG9-Hippocampus 1	G	FFPE	WT					Does not cluster with K27M group								
						DIPG9-Hippocampus 2	G	FFPE	WT					Does not cluster with K27M group								
						DIPG9-Thalamus 1	H	FFPE	H3.3 K27M (11.5%)					Does not cluster with K27M group					√	√	√	√
						DIPG9-Thalamus 2	H	FFPE	WT					Does not cluster with K27M group								
						DIPG9-Midbrain	I	Frozen				H3.3 K27M (48.57%)	ATRX V1514D (62.76%), PPM1D E525X (48.87%)		1 Core Punch	H3.3 K27M (49%)	H3.3 K27M	PPM1D E525X; ATRX V1514D				
						DIPG9-Pons 1	J	Frozen		H3.3 K27M (26%)		H3.3 K27M (31.42%)	ATRX V1514D (39.03%), PPM1D E525X (22.06%)			H3.3 K27M (24%)	H3.3 K27M	PPM1D E525X; ATRX V1514D				
						DIPG9-Pons 2	J	FFPE											√	√	√	√
						DIPG9-Medulla 1	K	Frozen				H3.3 K27M (46.6%)	ATRX V1514D (64.19%), PPM1D E525X (30.9%)			H3.3 K27M (4%)	H3.3 K27M	PPM1D E525X				
						DIPG9-Medulla 2	K	FFPE	H3.3 K27M (59%)					Clusters with K27M group					√	√	√	√

Supplementary Table 1: Clinical and molecular data from 134 punch cores taken from autopsy brains of 9 Diffuse Intrinsic Pontine Glioma (DIPG) patients analyzed in this study. Neuroanatomical locations: A = temporal lobe, B = frontal lobe, C = parietal lobe, D = occipital lobe, E = cerebellum, F = lateral ventricles, G = hippocampus, H = thalamus, I = midbrain, J = pons, K = medulla

Supplementary Table 2: Sample distribution from 9 DIPG patient samples used in this study

		MOLECULAR ANALYSES #			IHC only #	IHC and molecular	
**Patient ID**	**Total # of samples analyzed (IHC and molecular)**	**Total #**	**Brainstem #**	**Non brainstem #**		**Tumor #**	**Normal #**
**DIPG1**	16	8	6	2	8	13	3
**DIPG2**	28	26	5	21	2	9	19
**DIPG3**	4	4	2	2	0	2	2
**DIPG4**	13	6	5	1	7	8	5
**DIPG5**	9	6	4	2	3	6	3
**DIPG6**	11	9	7	2	2	8	3
**DIPG7**	26	25	6	19	1	12	14
**DIPG8**	26	26	9	17	0	16	10
**DIPG9**	25	24	4	20	1	7	18
**TOTAL**	158*	134**	48	86	24***	81	77
**AVERAGE**	18	15	5	10	3	9	9

Supplementary Table 2: Sample distribution and neuroanatomical locations from 9 DIPG patients (DIPG1-DIPG9) for all methods used in this study. *Number includes all samples used in this study. **Number excludes samples with only IHC data. ***Number of samples with only IHC data.

Supplementary Table 3: Tumor extension assessed by molecular analysis

Patient ID	Pons	Medulla	Midbrain	Cerebellum	Thalamus	Lateral Ventricles	Hippo-campus	Frontal Lobe	Occipital Lobe	Other Cerebral location
**DIPG1**	**(4/4)**	**(1/1)**	**(1/1)**	**(1/1)**				(0/1)		
**DIPG2**	**(4/4)**	**(1/1)**		**(1/6)**	**(1/1)**			(0/3)	(0/2)	
**DIPG3**	**(2/2)**			(0/1)					(0/1)	(0/1)
**DIPG4**	**(5/5)**							(0/1)		
**DIPG5**	**(1/1)**	**(1/1)**	**(1/2)**	(0/2)						
**DIPG6**	**(5/6)**		**(1/1)**	(0/1)				(0/1)		
**DIPG7**	**(3/4)**	**(1/1)**		**(1/4)**	**(1/2)**	**(2/2)**	**(1/2)**	**(1/2)**		
**DIPG8**	**(5/5)**	**(3/3)**	**(1/1)**	**(3/4)**	**(1/2)**	(0/1)	(0/2)	**(1/3)**	**(1/1)**	
**DIPG9**	**(1/1)**	**(2/2)**	**(1/1)**	(0/3)	**(1/2)**	**(1/1)**	(0/2)	(0/3)	(0/3)	
**# patients with tumor extension**	9	6	5	4	4	2	1	2	1	
**Total # patients analyzed**	9	6	5	8	4	3	3	7	4	
**Percentage**	100%	100%	100%	50%	100%	67%	33%	29%	25%	
**# samples with tumor extension**	31	9	5	6	4	3	1	2	1	
**Total # samples analyzed**	33	9	6	22	6	4	5	13	6	
**Percentage**	94%	100%	83%	27%	67%	75%	20%	15%	17%	

Supplementary Table 3: Tumor extension in different neuroanatomical locations tested by different molecular analyses (WES, MiSeq, ddPCR) for 9 Diffuse Intrinsic Pontine Glioma brain samples (DIPG1-DIPG9) analyzed in this study. Numbers in parentheses:(number of samples with H3-K27M/number of samples analyzed by molecular methods). **Cells in bold indicate samples with extension detected by molecular analysis.**

Supplementary Table 4: Whole Exome Sequencing data for 67 samples (9 Patients; DIPG1-DIPG9)

	K27M Marker	Accompanying (potential) driver mutations			
DIPG1	HIST1H3B_p.K27M	PIK3R1_splicing	CTNNA2_p.A260S		
DIPG2	HIST1H3B_p.K27M	ACVR1_p.G328V	PIK3CA_p.H1047R	MAX_p.R51Q	PTEN_p.A126S
DIPG3	HIST2H3C_p.K27M	ACVR1_p.G328V	PIK3CA_p.H1047R		
DIPG4	H3F3A_p.K27M	ACVR1_p.R206H	TP53p.C3F		
DIPG5	H3F3A_p.K27M	TP53p.A175fs	PPM1D_p.W427X	ATRXp.D94fs	
DIPG6	H3F3A_p.K27M	TP53_p.G113D	PIK3CA_p.H1047R	ATRXsplicing	OLIG2p.P215fs
DIPG7	H3F3A_p.K27M	TP53_p.R43H			
DIPG8	H3F3A_p.K27M	TP53_p.E162X	TP53p.P58fs		
DIPG9	H3F3A_p.K27M	PPM1D_p.E525X	ATRX_p.V1514D		

Supplementary Table 4: Summary of Whole Exome Sequencing data for 67 samples from 9 Diffuse Intrinsic Pontine Gliomas patients (DIPG1-DIPG9) demonstrating the main driver mutations as well as the accessory mutations.

Supplementary Table 5 Whole Exome Sequencing-based CNV calling

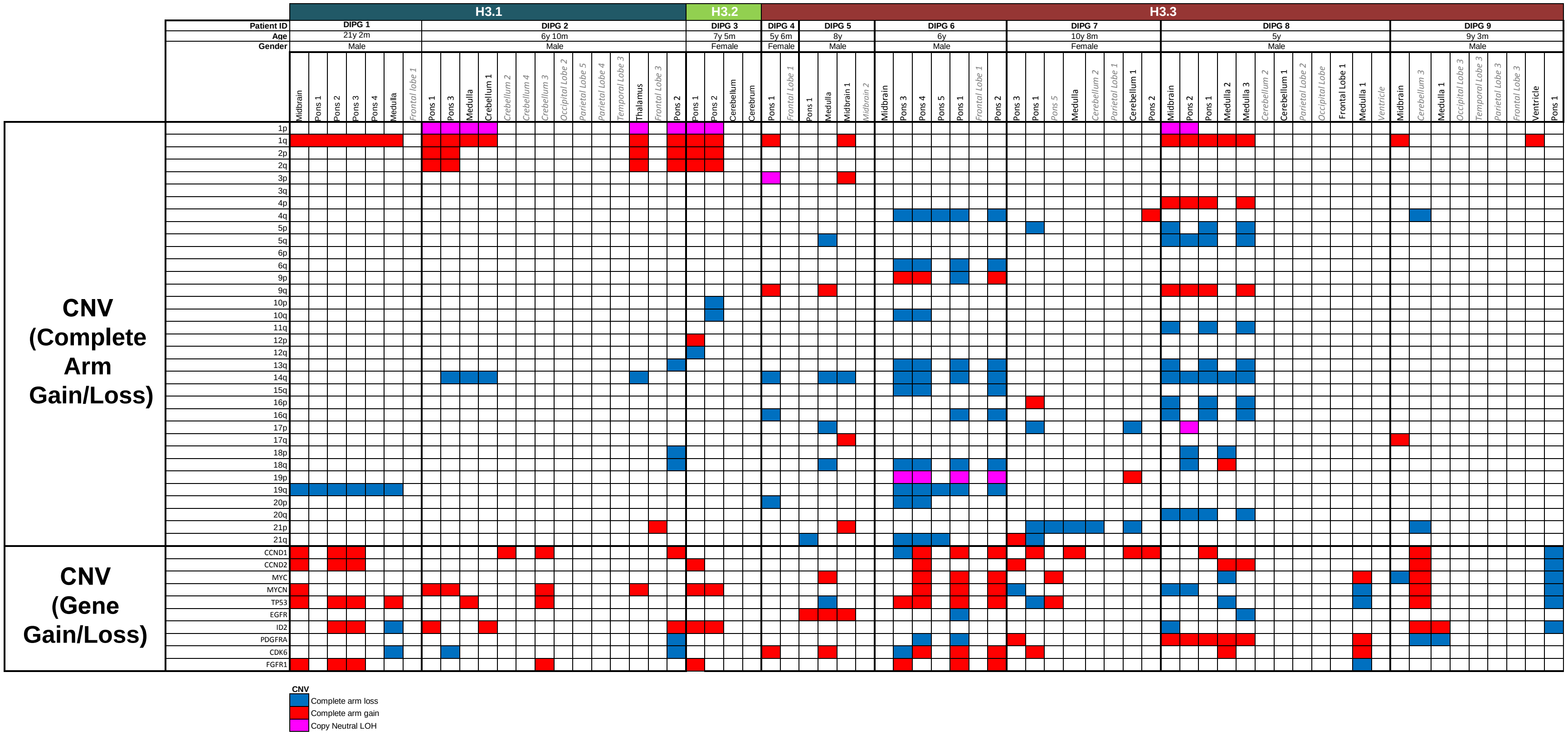

Supplementary Table 5: Copy Number Variation (CNV) calling based on Whole Exome Sequencing data for 67 samples from 9 Diffuse Intrinsic Pontine Glioma patients (DIPG1-DIPG9).

Supplementary Table 6: Integrated dataset of DIPG samples from four published studies (n=121) and present work (n=9)

Sample	Tumor Grade	Age	Gender	H3 mutation	ACVR1	TP53	PPM1D	PIK3CA	PIK3R1	PTEN	EGFR	FGFR1	PDGFRA	Other mutations	References
SJHGG106_A	IV	3.72	F	H3.1 K27M	R258G			H1047R							Wu et al. 2014
SJHGG074_D	IV	4.63	F	H3.1 K27M	R258G			I102_V105del							Wu et al. 2014
SJHGG056_A	IV	5.16	F	H3.1 K27M	R258G				L380del						Wu et al. 2014
SJHGG118_A	IV	3.40	M	H3.1 K27M	G328V				K567_I571>I						Wu et al. 2014
SJHGG008_A	IV	3.66	F	H3.1 K27M	G356D	R273C									Wu et al. 2014
SJHGG070_A	N/A	4.42	F	H3.1 K27M	G328E	G245C									Wu et al. 2014
SJHGG069_A	III	8.40	F	H3.1 K27M	G328E								Amp (Microarray CNV)		Wu et al. 2014
SJHGG047_A	IV	4.27	M	H3.1 K27M	G328E										Wu et al. 2014
SJHGG065_A	IV	6.58	M	H3.1 K27M	R206H										Wu et al. 2014
SJHGG077_A	IV	4.20	F	H3.1 K27M	R206H										Wu et al. 2014
SJHGG079_A	N/A	5.68	M	H3.1 K27M	R258G										Wu et al. 2014
SJHGG117_A	IV	2.58	F	H3.1 K27M	G328E			H1047R			R108K				Wu et al. 2014
SJHGG061_A	IV	6.90	F	H3.3 K27M	G328V			P449_L456>L					Amp (Microarray CNV)	ATRX gC335>FR	Wu et al. 2014
SJHGG058_A	IV	5.20	F	H3.3 K27M	G356D	E180fs									Wu et al. 2014
SJHGG002_A	IV	14.95	F	H3.3 K27M		R213X		E545K							Wu et al. 2014
SJHGG048_A	IV	6.12	M	H3.3 K27M		R156_R158>R (LOH)		E545K					Amp (Microarray CNV)		Wu et al. 2014
SJHGG057_A	IV	12.30	F	H3.3 K27M			W427X	E110del							Wu et al. 2014
SJHGG050_A	IV	5.70	F	H3.3 K27M			I466_P467fs		K575_R577del						Wu et al. 2014
SJHGG006_A	IV	3.66	F	H3.3 K27M		gE224A (LOH)									Wu et al. 2014
SJHGG102_D	IV	4.75	F	H3.3 K27M		R273C, Q144X									Wu et al. 2014
SJHGG105_A	IV	3.35	F	H3.3 K27M		E339X, R248W									Wu et al. 2014
SJHGG051_A	IV	15.70	M	H3.3 K27M		S241F							Amp (Microarray CNV)	NF1 I719fs (LOH)	Wu et al. 2014
SJHGG010325_A1	IV	10.23	M	H3.3 K27M		R273H							Amp (Microarray CNV)		Wu et al. 2014
SJHGG062_A	IV	12.50	F	H3.3 K27M		S241Y							N659K		Wu et al. 2014
SJHGG001_A	IV	5.33	F	H3.3 K27M		R273C (LOH)							A529>15aa		Wu et al. 2014
SJHGG045_A	N/A	11.78	M	H3.3 K27M		M237I (LOH)									Wu et al. 2014
SJHGG060_A	IV	13.27	M	H3.3 K27M		R248Q (LOH)									Wu et al. 2014
SJHGG073_A	N/A	11.10	M	H3.3 K27M		R273C, K120M									Wu et al. 2014
SJHGG007_A	IV	10.90	M	H3.3 K27M		S241F								NF1 R816X, SV (NF1_CNTN5)	Wu et al. 2014
SJHGG053_A	IV	7.31	F	H3.3 K27M		V173A									Wu et al. 2014
SJHGG055_A	IV	6.43	F	H3.3 K27M		H193Y									Wu et al. 2014
SJHGG101_A	IV	17.24	F	H3.3 K27M		C176Y									Wu et al. 2014
SJHGG004_D	IV	5.47	M	H3.3 K27M		L130_N131>L									Wu et al. 2014
SJHGG010324_A1	N/A	9.56	M	H3.3 K27M		R174_E180>R									Wu et al. 2014
SJHGG009_A	IV	6.01	M	H3.3 K27M		R303X									Wu et al. 2014
SJHGG066_A	IV	9.00	F	H3.3 K27M		R234C							Amp (Microarray CNV)		Wu et al. 2014
SJHGG049_A	IV	13.77	F	H3.3 K27M										CCND2 (Amp)	Wu et al. 2014
SJHGG054_A	IV	3.96	F	H3.3 K27M		G66V									Wu et al. 2014
SJHGG067_A	IV	5.60	F	H3.3 K27M											Wu et al. 2014
SJHGG109_A	IV	8.64	F	H3.3 K27M											Wu et al. 2014
SJHGG068_A	IV	2.97	M	H3.3 K27M		Y163C									Wu et al. 2014
SJHGG059_A	IV	8.90	M	WT	G328V			E545K	T576_R577>R						Wu et al. 2014
SJHGG064_A	IV	10.20	M	WT	G328W		Q404X	H1047R							Wu et al. 2014
SJHGG071_A	N/A	6.87	F	H3.3 K27M	G328E		S516X	E545K						ATRX T1610R	Wu et al. 2014
SJHGG005_A	IV	5.35	F	WT	G328V										Wu et al. 2014
SJHGG003_A	IV	15.58	M	WT		P47_F54fs (LOH)							Amp (Microarray CNV), SV (DIP2C_PDGFRA)		Wu et al. 2014
SJHGG052_A	IV	5.94	F	WT		R158G (LOH)									Wu et al. 2014
SJHGG063_A	IV	5.40	F	WT											Wu et al. 2014
SJHGG076_A	IV	10.25	F	H3.3 K27M			E405X								Wu et al. 2014
SJHGG078_A	IV	6.46	M	WT											Wu et al. 2014
SJHGG075_A	IV	1.82	M	WT		A88fs							Amp (Microarray CNV)		Wu et al. 2014
mHGA1	IV	10	M	H3.3 K27M										CHEK2 splicing	Fontebasso et al. 2014
mHGA2	IV	3.5	F	H3.3 K27M	R206H			E545K							Fontebasso et al. 2014
mHGA3	IV	5	M	H3.3 K27M		R273H									Fontebasso et al. 2014
mHGA4	IV	3	F	H3.3 K27M		IHC POSITIVE									Fontebasso et al. 2014
mHGA14	IV	10	F	H3.3 K27M		R282W							Amp (450K CNV), I543_V544	NF1 LOSS (450K CNV)	Fontebasso et al. 2014
mHGA15	IV	8	F	H3.3 K27M		R273P									Fontebasso et al. 2014
mHGA16	IV	12	M	H3.3 K27M		R248W									Fontebasso et al. 2014
mHGA17	IV	6	F	H3.3 K27M		R156Pfs*17)							N328S	SETD2 H1035D	Fontebasso et al. 2014
mHGA18	IV	11	F	H3.3 K27M		R248W		V344G		LOSS (450K CNV)			Amp (450K CNV)		Fontebasso et al. 2014
mHGA19	IV	7	M	H3.3 K27M		A159Pfs*11, LOSS (450K CNV)									Fontebasso et al. 2014
mHGA20	IV	7	M	H3.3 K27M		V173L									Fontebasso et al. 2014
mHGA21	IV	4.5	F	H3.3 K27M		R175H									Fontebasso et al. 2014
mHGA22	IV	4	M	H3.3 K27M		S241P									Fontebasso et al. 2014
mHGA23	IV	7	F	H3.3 K27M		V157F								ATRX Q1034fs	Fontebasso et al. 2014
mHGA24	IV	13	F	H3.3 K27M		P152Rfs*27			K447_Y452del					ATRX C1122Vfs*8)	Fontebasso et al. 2014
mHGA26	III	14	F	H3.3 K27M								N546K, 655_655del		ATRX L2240R	Fontebasso et al. 2014
mHGA30	IV	6	M	H3.1 K27M		E258G									Fontebasso et al. 2014
mHGA31	IV	4	M	H3.1 K27M	R258G				L449_H450delinsY						Fontebasso et al. 2014
mHGA32	IV	3	M	H3.1 K27M	G328E			C420R							Fontebasso et al. 2014
mHGA33	III	3	F	H3.1 K27M	G328V		C478X	Y1038F, H1047R							Fontebasso et al. 2014
mHGA34	IV	9	F	H3.1C K27M	G356D										Fontebasso et al. 2014
mHGA35	IV	17	M	WT		V173L, LOSS (450K CNV)							Amp(450K CNV)		Fontebasso et al. 2014

mHGA38	IV	6	M	H3.3 K27M		R158G									Fontebasso et al. 2014
mHGA39	IV	7	F	H3.3 K27M		S241F									Fontebasso et al. 2014
mHGA40	IV	8	M	H3.3 K27M		Y220C									Fontebasso et al. 2014
HSJD_DIPG002		6	F	H3.1 K27M	R258G			H1047R							Taylor et al. 2014
NCHP_DIPG011		4.8	F	H3.1 K27M	G328E				573-576 del					IGF2R K162R	Taylor et al. 2014
NCHP_DIPG108		7.5	M	H3.1 K27M	G328V					R130X					Taylor et al. 2014
HSJD_DIPG004		10	F	H3.1 K27M	G328E										Taylor et al. 2014
NCHP_DIPG052		4.6	M	H3.1 K27M	G328V										Taylor et al. 2014
NCHP_DIPG113		4.6	M	H3.1 K27M	G356D										Taylor et al. 2014
NCHP_DIPG114		8.6	M	H3.1 K27M				Q546K							Taylor et al. 2014
NCHP_DIPG103		5.8	F	H3.1 K27M		E221X									Taylor et al. 2014
NCHP_DIPG081		6.7	M	H3.3 K27M		R273H									Taylor et al. 2014
NCHP_DIPG105		6.6	F	H3.3 K27M		R175H									Taylor et al. 2014
NCHP_DIPG061		11.9	F	H3.3 K27M		V157F									Taylor et al. 2014
NCHP_DIPG102		10.3	M	H3.3 K27M		C135Y									Taylor et al. 2014
HSJD_DIPG001		6	F	H3.3 K27M		G244S									Taylor et al. 2014
NCHP_DIPG112		5.6	F	H3.3 K27M		R273C									Taylor et al. 2014
NCHP_DIPG111		10.6	F	H3.3 K27M		156fs									Taylor et al. 2014
NCHP_DIPG109		6.2	M	H3.3 K27M		R273C									Taylor et al. 2014
NCHP_DIPG065		10.2	M	H3.3 K27M		A159V			K567E						Taylor et al. 2014
NCHP_DIPG006		6.3	M	H3.3 K27M			L513X	E542K							Taylor et al. 2014
HSJD_DIPG003		6	M	H3.3 K27M			S468X	H1047R							Taylor et al. 2014
HSJD_DIPG008		6.5	M	H3.3 K27M			429fs								Taylor et al. 2014
NCHP_DIPG107		8.8	M	H3.3 K27M					K379E					ATM 1190-1191fs	Taylor et al. 2014
NCHP_DIPG101		3.9	F	H3.3 K27M											Taylor et al. 2014
HSJD_DIPG007		9.9	M	H3.3 K27M	R206H		428fs	C420R							Taylor et al. 2014
HSJD_DIPG106		12.1	M	WT			483fs	H1047R						IGF2R D1830R	Taylor et al. 2014
HSJD_DIPG104		4.4	M	WT		R282W								NF1 R192X	Taylor et al. 2014
HSJD_DIPG110		5.7	F	WT											Taylor et al. 2014
DIPG03	GBM	NA	M	H3.1C K27M		1									Buczkowicz et al. 2014
DIPG62	AA	NA	M	H3.1 K27M	G328E	R43H									Buczkowicz et al. 2014
DIPG26	LGA	NA	F	H3.3 K27M		S339P		E545G							Buczkowicz et al. 2014
DIPG07	GBM	NA	M	H3.3 K27M		R43H									Buczkowicz et al. 2014
DIPG08	GBM	NA	F	H3.3 K27M		R26fs									Buczkowicz et al. 2014
DIPG24	GBM	NA	F	H3.3 K27M		R141C									Buczkowicz et al. 2014
DIPG27	GBM	NA	M	H3.3 K27M		1									Buczkowicz et al. 2014
DIPG57	GBM	NA	F	H3.3 K27M		1									Buczkowicz et al. 2014
DIPG60	GBM	NA	M	H3.3 K27M		1									Buczkowicz et al. 2014
DIPG06	GBM	NA	M	H3.3 K27M		S109P									Buczkowicz et al. 2014
DIPG19	GBM	NA	F	H3.3 K27M		V41L									Buczkowicz et al. 2014
DIPG28	AA	NA	M	H3.3 K27M		1							T281P		Buczkowicz et al. 2014
DIPG30	GBM	NA	M	H3.3 K27M	1										Buczkowicz et al. 2014
DIPG04	GBM	NA	M	H3.3 K27M		V25F									Buczkowicz et al. 2014
DIPG61	GBM	NA	M	H3.3 K27M		1									Buczkowicz et al. 2014
DIPG58	GBM	NA	F	H3.3 K27M					Q325X, N344del						Buczkowicz et al. 2014
DIPG25	AA	NA	F	H3.3 K27M											Buczkowicz et al. 2014
DIPG29	GBM	NA	F	WT											Buczkowicz et al. 2014
DIPG18	GBM	NA	M	WT		1									Buczkowicz et al. 2014
DIPG1	GBM	21.2	M	H3.1 K27M					Splicing					CTNNA2 A260S	Present study
DIPG2	GBM	6.10	M	H3.1 K27M	G328V			H1047R		A126S				MAX R51Q	Present study
DIPG3	GBM	7.5	F	H3.2C K27M	G328V			H1047R							Present study
DIPG4	GBM	5.6	F	H3.3 K27M	R206H	C3F									Present study
DIPG5	GBM	8	M	H3.3 K27M		A175fs	W427X							ATRX D94fs	Present study
DIPG6	Astrocytoma	6	M	H3.3 K27M		G113D		H1047R						ATRX splicing, OLIG2 P215fs	Present study
DIPG7	GBM	10.8	F	H3.3 K27M		R43H									Present study
DIPG8	GBM	5	M	H3.3 K27M		P58fs, E162X									Present study
DIPG9	GBM	9.3	M	H3.3 K27M			E525X							ATRX V1514D	Present study

Supplementary Table 6: Integrated dataset of Diffuse Intrinsic Pontine Glioma samples from four published studies (n=121) and present work (n=9) showing ditribtuion of histone mutations.

Supplementary Table 7: Mutation analysis of biopsy (obtained at diagnosis) and autopsy pairs from the same patients

Sample Name	Type	H3 Mutation	Other Accompanying Mutations	
SJHGG001	Autopsy	H3.3 K27M	TP53_p.R273C (LOH)	PDGFRA_p.A529>15aa
	Biopsy	H3.3 K27M	TP53_p.R273C (LOH)	PDGFRA_p.A529>15aa
SJHGG002	Autopsy	H3.3 K27M	TP53_p.R213*	PIK3CA_p.E545K
	Biopsy	H3.3 K27M	TP53_p.R213*	TP53_p.L252P
mHGA18	Autopsy	H3.3 K27M	TP53_p.R248W	PIK3CA_p.V344G
	Biopsy	H3.3 K27M	TP53_p.R248W	PIK3CA_p.V344G

Supplementary Table 7: Mutation status of the main histone drivers and accompanying genes obtained from analysis of samples acquired at biopsy and autopsy from three Diffuse Intrinsic Pontine Glioma (DIPG) patients.

Discussion

Genomic analysis of autopsy DIPG brains provides a fascinating opportunity to reconstruct the evolution of this deadly tumor, and has important implications on potential therapeutic interventions. Despite the sample number limitation based on disease incidence, this study provides insight into the spatial and temporal evolution of the tumor genome in DIPG helping derive a general model for this disease.

Our analysis suggests that H3K27M mutations are the initial oncogenic event in DIPGs. However, these mutations are not solely sufficient for tumor formation, as they require, nearly universally, obligate partners for effective tumorigenesis. There is some disagreement in the cancer field as to the definition of “driver” versus “passenger” mutations. The designation passenger has been used for any variant that is not absolutely essential to the tumorigenesis. However, for the purpose of this study – and more generally – we postulate three categories of mutations: 1) Main driver mutations are essential to initiate and continue tumorigenesis; 2) Accessory driver mutations can further promote and accelerate tumor growth but are not absolutely essential; 3) Passenger mutations are neutral and do not affect the tumor. H3K27M and their obligate partners appear thus to be the main driver mutations, since they always co-occur together throughout each tumor.

These K27MH3.3 partner mutations are mostly genetic alterations affecting the TP53 pathway, mainly *TP53* and less frequently *PPM1D* genes. H3K27M mutants also favor association with *ACVR1* gain of function mutations and in rare cases (1 in our dataset and 2 in other reported cases) *PIK3R1* mutations. Interestingly, even if more frequent, PIK3CA mutations, including PIK3CAH1047R, clearly fall into the accessory driver category, since they were invariably sub-

clonal in our dataset and in some other reported cases. Similarly, a PTEN mutation was also found in some but not all tumor areas in DIPG2 (Fig. 3). We can only speculate at this time as to why mutations that ultimately activate the PI3K/AKT pathway appear clonal for some of the component genes and subclonal for others. This may be related to the dosage effect of pathway activation, as the loss of the regulatory unit (*PIK3R1*) leads to increased baseline activity of the wild-type kinase whereas the PIK3CAH1047R mutation has been associated with higher kinase activity and oncogenic potential through cellular reprograming and induction of stemness properties[24]. Further studies are needed to help ascertain the specific role of these accessory driver mutations in oncohistone tumorigenesis.

Once acquired, the main driver partnership is maintained throughout the course of the disease (diagnosis to autopsy), in all cells across the primary tumor site, and in tumor spread throughout the brain. The obligate partner is not chosen randomly. One speculation about the phenotypic advantage of the gain of additional mutations may be that the partners are selected as those best suited to increase levels of mutant H3 in cells. Indeed, H3K27M mutants show a dose-dependent effect in inducing cellular proliferation[26,27]. Canonical H3 variants are the most abundant histones in cells and need cell-cycle division (S-phase) for synthesis, whereas non-canonical H3.3 variants represent ~5% of all H3 in cells and are present throughout the cell cycle. Accordingly, H3K27M mutations in cell cycle-dependent canonical histones may co-occur with alterations affecting ACVR1, a growth factor that induces cell division, resulting in the synthesis of wildtype and mutant H3.1 proteins. H3K27M mutations in the non-canonical H3.3 histone are mainly associated with mutations affecting the TP53 pathway, as these possibly offer evasion from cell death and senescence and provide the needed opportunity for the mutant H3.3K27M to

exert its effect over a longer period, effectively reshaping the epigenome and drive tumor formation.

The previously unsuspected homogeneity for main driver mutations across the course of the disease we uncover in this study indicates that efforts to cure DIPG should be directed at the oncohistone partnership, as other genetic alterations are generally sub-clonal. Our findings further indicate that needle biopsies recommended to orient care are representative of the main drivers in DIPG even if the regional heterogeneity of other secondary targetable alterations, such as PIK3CA mutations, may not be fully captured. Based on early tumor spread, efforts to cure DIPG should aim for early systemic tumor control as opposed to regimens focused on the pons.

Methods

Patient samples

All patient samples were collected with informed consent in accordance with the respective Ethics Review Boards of the institutions that provided them. DIPG post-mortem specimen procurement was performed as previously described[19]. Briefly, brainstem and cerebellum were removed *en bloc* from the whole brain, and dissected into ~9 transverse sections. The cerebral cortex was dissected into ~11 coronal sections. The brainstem, cerebellum, and cerebral cortex sections were alternatively frozen or fixed in formalin. A total of 158 samples were studied by immunohistochemistry and molecular analyses, representing various neuroanatomical locations such as frontal, parietal, temporal, occipital lobes, thalamus, lateral ventricles, hippocampus, midbrain, pons, medulla, and cerebellum (Supplementary Tables 1-3; Supplementary Fig. 1). For molecular studies (RNA, DNA) 134 core punches were obtained using a biopsy punch and plunger (2mm, #33-31, Integra Miltex, York, PA). All histological sections were reviewed by neuropathologists (C-Y.H.; J.K.), according to the World Health Organization (WHO) classification of tumors. Demographical, clinical and histopathological characteristics of all specimens are presented in Supplementary Table 1.

Immunohistochemistry

Immunohistochemistry was performed on 5μm thick FFPE slides. Briefly, slides were de-paraffinized, processed for epitope retrieval, DAB detected using reagents customized for the Leica BOND-MAX automated stainer (Leica Biosystems, Buffalo Grove, IL). Processed slides were probed by immunohistochemical assay for hematoxylin and eosin (H&E), Ki67, H3-K27M, and H3-K27me3 as previously described[12] (Supplementary Fig. 2).

Antibodies

Rabbit monoclonal anti-Ki67 (Biocare Medical, Concord, CA) were pre-diluted and ready to use. Rabbit polyclonal anti-H3K27M (#ABE419 Millipore, Billerica, MA, 1:500), rabbit monoclonal anti-H3K27me3 (C36B11, #9733 Cell Signaling, Beverly, MA 1:75) were diluted in Bond primary antibody diluent (#AR9352 Leica Biosystems, Buffalo Grove, IL). Secondary detection was conducted using the Bond polymer refined detection kit (Leica Biosystems, Buffalo Grove, IL). Slides were counterstained for hematoxylin nuclear stain.

RNA and DNA extractions

Frozen tissue samples were homogenized with Trizol, and nucleic acids were phase separated using chloroform. Total RNA was extracted according to the PicoPure RNA Isolation kit (Arctrus Bioscience Inc. Mountain view, CA). Genomic DNA was extracted from frozen tissue using the Gentra Puregene DNA extraction kit, or from FFPE tissue using the QiaAmp DNA mini kit according to the manufacturer’s instructions (Qiagen, Valencia, CA). All DNA quantifications were conducted using the Quant-iT Picogreen dsDNA assay kit (Life Technologies, Carlsbad, CA).

Droplet Digital PCR

Digital droplet PCR (ddPCR) assays were performed according to standard methods. Briefly, each 20 ul reaction contained 1X ddPCR Supermix for Probes (Bio-Rad), 900 nM gene specific HPLC-purified forward and reverse primer, 250 nM gene-specific mutant or wild-type LNA probe and 12.5-25.0 ng genomic DNA. Each reaction was mixed with 60 ul Droplet Generation

Oil (Bio-Rad), partitioned into ~12,000 -16,000 droplets in QX100 Droplet Generator (Bio-Rad), transferred to a 96-well plate and sealed. The primers and probes were designed by Integrated DNA Technologies (IDT) as follows: forward primer for *H3F3A*: 5'-GTACAAAGCAGACTGCCCGCAAAT-3', reverse primer 5'-GTGGATACATACAAGAGAGACTTTGTCCC-3'. Forward primer for *HIST1H3B:* 5'-ACAGACGTCTCTGCAGGCAAGC-3', reverse primer 5'-GGCGGTAACGGTGAGGCTTT-3'. *H3F3A* K27M wild-type probe (HEX) CA+C+T+C+T+T+GC and mutant probe (FAM) CA+CT+C+A+T+GCG. *HIST1H3B* K27M wild-type probe (HEX) T+CGC+A+A+GAG+CG and mutant probe (FAM) TCGC+A+T+G+AGCG. The PCRs were performed in a T100 Thermal Cycler (Bio-Rad) with the following cycling conditions: 1× (95°C for 10 min), 50× (95°C for 30 s, 53°C or 57°C for 60 s, with 2°C /s ramp rate and 1× (98°C for 10 min). Following end-point amplification, the fluorescence intensity of individual droplets was measured with the QX100 Droplet Reader (Bio-Rad) and data analysis was performed with QuantaSoft droplet reader software (Bio-Rad). Positive and negative droplet populations were detected either automatically or manually on two-dimensional graphs and target DNA concentrations were calculated using the Poisson statistics[28].The absolute transcript levels were initially computed as copies/µl PCR for both mutant and wild-type and then presented as percent of total gene copy.

Whole-exome sequencing

Genomic DNA was extracted from multiple post-mortem samples patient using the standard extraction methods as described by Qiagen. Nextera Rapid Capture Exome kit was used to prepare the paired-end libraries according to the manufacturer's instructions using on average 36

ng of total starting genomic DNA. Sequencing was performed on Illumina HiSeq 2000 using rapid-run mode with 100 bp paired-end reads. Next, adaptor sequences were removed; reads were trimmed for quality using the FASTX-Toolkit. An in-house program was used to ensure the presence of exclusively paired-reads to be used in further steps of the analysis. We next aligned the reads using Burrows-Wheeler Aligner (BWA) 0.7.7 to hg19 as reference genome. Indel realignment was performed using the Genome Analysis Toolkit (GATK)[29] We next marked the duplicate reads using Picard and excluded them from further analyses as previously described[6]. The coverage of consensus coding sequence (CCDS) bases was assessed using GATK. The average coverage over all the samples was 70x. The majority of samples had >90% of CCDS bases covered by at least 10 reads and > 83% of CCDS bases covered by at least 20 reads.

We called SNVs and short indels using SAMtools mpileup with the extended base alignment quality (BAQ) adjustment (–E)[30,31]. Next we filtered them for quality so that at least 10% of reads supporting each variant call. We used both ANNOVAR[32] and in-house tools to annotate the variants and to identify whether these variants affect protein-coding sequence and if they had previously been observed in datasets including the 1000 Genomes Project data set (November 2011), the National Heart, Lung, and Blood Institute (NHLBI) Grand Opportunity (GO) exomes or in approximately 3,000 exomes previously sequenced at our center. Results of whole exome sequencing are summarized in Supplementary Table 4 and presented for individual patients as follows: DIPG1 in Supplementary Data 1; DIPG2 in Supplementary Data 2; DIPG3 in Supplementary Data 3; DIPG4 in Supplementary Data 4; DIPG5 in Supplementary Data 5; DIPG6 in Supplementary Data 6; DIPG7 in Supplementary Data 7; DIPG8 in Supplementary Data 8; DIPG9 in Supplementary Data 9.

Exploratory targeted high-depth DNA sequencing of hotspot mutations

Genomic DNA from DIPG samples were used for high-depth sequencing using Illumina MiSeq platform. The MiSeq panel covers exons in 16 Histone H3 isoforms (10 H3.1, 2 H3.2 and 3 H3.3 genes) and covering hotspot mutations such as *IDH1* mutation (codon 132), *IDH2* (codons 140 and 172), *ACVR1* (exons 6-9) and *BRAF* (exons 11 and 15) and *PPM1D* (exon 6). Genomic DNA samples were sequenced using the MiSeq sequencing platform (Illumina) as previously described[6] with an average coverage of > 20,000X of the analogous K27M base change across the three histone variants.

To estimate allele frequency of mutations identified using whole exome sequencing genomic DNA from the same samples was also used for high-depth sequencing on the MiSeq platform with an average coverage of > 4,000 x. Reads were mapped to the reference genome (human hg19) using the BWA genome alignment[30,31]. Alignment files were fed to an in-house program to calculate different variations' allele frequencies at the desired positions.

RNA Sequencing

We used Qiagen RNeasy Lipid Tissue Mini kit to extract RNA from tumor DIPG3 (Pons 1 and Pons 2) according to manufacturer's instructions. Library was prepared using rRNA depletion methods according to instruction from Epicentre (manufacturer) to achieve greater coverage of mRNA. Paired-end sequencing was performed on the Illumina HiSeq 2000 platform.

DNA Methylation Analysis

Methylation profiling data was analyzed as previously described[6]. The raw data were subject to quality control and preprocessing utilizing the R package minfi, and normalized for technical

variation between the Infinium I and II probes using the SWAN method. We removed probes on sex chromosomes (chrX, Y), those containing SNPs (dbSNP: http://www.ncbi.nlm.nih.gov/SNP/) as well as non-specific probes that bind to multiple genomic locations. Unsupervised hierarchical clustering was performed using average linkage and Pearson rank correlation distance on the top 5,000 most variable probes selected based on standard deviation of beta values (β-values).

Copy Number Variation analysis

To study copy number variations in our samples we developed an in-house program to calculate the deviation of B allele frequency from 50% as well as normalized coverage from whole exome sequencing data (adapted from methods used in FishingCNV[33] and ExomeAI[34]). Different CNV events (duplication, deletion, copy neutral LOH) were called based upon the B allelic imbalance and the status of the normalized coverage as follow: Deviation from 50% B allele frequency and an increase in normalized coverage was considered as amplification, Deviation from 50% B allele frequency and decrease in normalized coverage as deletion, and Deviation from 50% B allele frequency and no change in the normalized coverage was considered as potential copy neutral loss of heterozygosity. We mainly assessed the CNV events at the chromosomal arm level. The results of our CNV detection are presented in Supplementary Fig. 5 and Supplementary Table 14.

OncoScan verification of the CNV events

OncoScan® FFPE Assay Kit, provided by Affymetrix, is a platform based on Molecular Inversion Probe (MIP) technology, used to asses copy number and loss of heterozygosity using

small amounts of DNA from FFPE samples. We performed this assay on several samples in order to verify our WES based CNV detection method. Genomic DNA was quantified using Picogreen protocol (Quant-iT™ PicoGreen® dsDNA Products, Invitrogen, P-7589) and read on SpectraMAX GeminiXS Spectrophotometer. The OncoScan® FFPE Assay Kit was used according to the manufacturer's instructions (Affymetrix). A GeneAmp PCR system 9700 Thermal Cycler (Life Technologies) was used from the Anneal stage to the Denaturation stage. QC gels of the PCR and HaeIII digest products were performed on E-Gel® 48 4% Agarose Gels using Mother E-Base™ Device (Life Technologies) and imaged with SYNGENE GeneGenius Bio Imaging System (Syngene). The digest DNA target was hybridized on OncoScan® Array, (Affymetrix) and incubated at 49^0C in the Genechip® Hybridization oven 640 (Affymetrix) for 17 hours at 60 rpm. OncoScan® Arrays were then washed in a GeneChips® Fluidics Station 450 (Affymetrix) using OncoScan® Stain and Wash Reagents according to the manufacturer's instructions (Affymetrix). The microarrays were finally scanned on a GeneChip® scanner 3000 (Affymetrix). Data QC analysis was performed with the OncoScan Consol 1.2.0.50 software (Affymetrix) using OncoScan Analysis Library files r1.1. OncoScan® Positive and negative Control supplied in the OncoScan® FFPE Assay Kit were used for internal controls to assess the performance of each run. CNV events were called using the normalized data using Nexus Express for OncoScan 3.1 (Affymetrix). The OncoScan plots are represented in Supplementary Fig. 6.

Constructing evolutionary trees

We used PhyloWGS[35] to reconstruct the tumor phylogeny, which uses a Bayesian approach to infer cellular frequencies from mutation allele frequencies. It applies Dirichlet process to cluster

mutations with similar cellular frequencies and the tree-structured stick-breaking process to model the clonal evolutionary tree. For multi-region samples from the same patient, we normalized the read counts used for phylogenetic tree construction by copy number counts from CNV analysis. Read counts were corrected for the CNV events (Supplementary Fig. 5; Table 3) for each sample as follow: in case of duplication Ref' = Ref, Alt' = Alt/2; in case of deletion Ref' = Ref + Alt, Alt' = Alt; in case of a gene on chromosome X Ref' = Ref *2 + Alt, Alt' = Alt; in case of Copy Neutral LOH Ref' = Ref+Alt/2, and Alt' = Alt/2. The reconstructed trees were redrawn in Graphviz by using in-house scripts adapted from AncesTree[36] to show the trajectories of mutations with contribution greater than 0.05.

Differential Expression and Gene Set Enrichment Analysis

We aligned RNASeq data from DIPG3-Pons 1 and DIPG3-Pons 2 (RNA extraction and sequencing protocol described before) using STAR 2.3.0[37], and analyzed differentially expressed genes using DeSEQ2[36] The top 200 over expressed and top 200 under expressed genes in DIPG3-Pons 2 (the sub-clone with PIK3CA activating mutation) were analyzed for geneset enrichment using both AmiGo 2 tool[38] provided by Gene Ontology and DAVID[39]. We used PANTHER Overrepresentation Test (release 20150430) for analysis type by Amigo 2 (annotation version and release date: GO Ontology database Released 2015-08-06.) The top pathways found by AmiGo 2 to be enriched with enrichment folds higher than 5 and (Bonferroni < 0.05) were retained. We used functional annotation clustering and set the stringency to the highest in DAVID and filtered the results for enrichment folds higher than 5 (Bonferroni corrected *p-value* < 0.05.) We used the set of genes with at least 50 RNASeq reads in both DIPG3 Pons1 and 2 combined as background gene set in this analysis (Supplementary Data 10).

URLs

FASTX-Toolkit, http://hannonlab.cshl.edu/fastx_toolkit/; Genome Analysis Toolkit (GATK), http://www.broadinstitute.org/gsa/wiki/; Picard, http://picard.sourceforge.net/; SAMtools, http://samtools.sourceforge.net/; dbSNP, http://www.ncbi.nlm.nih.gov/SNP/.

Amigo 2, http://amigo.geneontology.org/amigo; DAVID, https://david.ncifcrf.gov/.

Accession Numbers

Whole-exome sequencing data for all tumors (along with RNA sequencing data for DIPG3), and also DNA methylation profiles can be accessed through the European Genome Archive (EGA) at the following accession EGAS00001001654 and Gene Expression Omnibus (GEO) under accession GSE77353 respectively.

References

1. Buczkowicz, P., Bartels, U., Bouffet, E., Becher, O. & Hawkins, C. Histopathological spectrum of paediatric diffuse intrinsic pontine glioma: diagnostic and therapeutic implications. *Acta Neuropathol* **128**, 573-581 (2014).
2. Khuong-Quang, D.A. *et al.* K27M mutation in histone H3.3 defines clinically and biologically distinct subgroups of pediatric diffuse intrinsic pontine gliomas. *Acta Neuropathol* **124**, 439-447 (2012).
3. Schwartzentruber, J. *et al.* Driver mutations in histone H3.3 and chromatin remodelling genes in paediatric glioblastoma. *Nature* **482**, 226-231 (2012).
4. Wu, G. *et al.* Somatic histone H3 alterations in pediatric diffuse intrinsic pontine gliomas and non-brainstem glioblastomas. *Nat Genet* **44**, 251-253 (2012).
5. Buczkowicz, P. *et al.* Genomic analysis of diffuse intrinsic pontine gliomas identifies three molecular subgroups and recurrent activating ACVR1 mutations. *Nat Genet* **46**, 451-456 (2014).
6. Fontebasso, A.M. *et al.* Recurrent somatic mutations in ACVR1 in pediatric midline high-grade astrocytoma. *Nat Genet* **46**, 462-466 (2014).
7. Taylor, K.R. *et al.* Recurrent activating ACVR1 mutations in diffuse intrinsic pontine glioma. *Nat Genet* **46**, 457-461 (2014).
8. Wu, G. *et al.* The genomic landscape of diffuse intrinsic pontine glioma and pediatric non-brainstem high-grade glioma. *Nat Genet* **46**, 444-450 (2014).
9. Kumar, A. *et al.* Deep sequencing of multiple regions of glial tumors reveals spatial heterogeneity for mutations in clinically relevant genes. *Genome Biol* **15**, 530 (2014).

10. Sottoriva, A. *et al.* Intratumor heterogeneity in human glioblastoma reflects cancer evolutionary dynamics. *Proc Natl Acad Sci U S A* **110**, 4009-4014 (2013).

11. Szerlip, N.J. *et al.* Intratumoral heterogeneity of receptor tyrosine kinases EGFR and PDGFRA amplification in glioblastoma defines subpopulations with distinct growth factor response. *Proc Natl Acad Sci U S A* **109**, 3041-3046 (2012).

12. Bechet, D. *et al.* Specific detection of methionine 27 mutation in histone 3 variants (H3K27M) in fixed tissue from high-grade astrocytomas. *Acta Neuropathol* **128**, 733-741 (2014).

13. Caretti, V. *et al.* Subventricular spread of diffuse intrinsic pontine glioma. *Acta Neuropathol* **128**, 605-607 (2014).

14. Gerlinger, M. *et al.* Intratumor heterogeneity and branched evolution revealed by multiregion sequencing. *N Engl J Med* **366**, 883-892 (2012).

15. Eleveld, T.F. *et al.* Relapsed neuroblastomas show frequent RAS-MAPK pathway mutations. *Nat Genet* **47**, 864-871 (2015).

16. Wu, X. *et al.* Clonal selection drives genetic divergence of metastatic medulloblastoma. *Nature* **482**, 529-533 (2012).

17. Johnson, B.E. *et al.* Mutational analysis reveals the origin and therapy-driven evolution of recurrent glioma. *Science* **343**, 189-193 (2014).

18. Walker, D.A. *et al.* A multi-disciplinary consensus statement concerning surgical approaches to low-grade, high-grade astrocytomas and diffuse intrinsic pontine gliomas in childhood (CPN Paris 2011) using the Delphi method. *Neuro Oncol* **15**, 462-468 (2013).

19. Kambhampati, M. *et al.* A standardized autopsy procurement allows for the comprehensive study of DIPG biology. *Oncotarget* **6**, 12740-12747 (2015).

20. Castel, D. *et al.* Histone H3F3A and HIST1H3B K27M mutations define two subgroups of diffuse intrinsic pontine gliomas with different prognosis and phenotypes. *Acta Neuropathol* **130**, 815-827 (2015).

21. Nik-Zainal, S. *et al.* Mutational processes molding the genomes of 21 breast cancers. *Cell* **149**, 979-993 (2012).

22. Popic, V. *et al.* Fast and scalable inference of multi-sample cancer lineages. *Genome Biol* **16**, 91 (2015).

23. Zhang, L. *et al.* Exome sequencing identifies somatic gain-of-function PPM1D mutations in brainstem gliomas. *Nat Genet* **46**, 726-730 (2014).

24. Koren, S. *et al.* PIK3CA(H1047R) induces multipotency and multi-lineage mammary tumours. *Nature* **525**, 114-118 (2015).

25. Karar, J. & Maity, A. PI3K/AKT/mTOR Pathway in Angiogenesis. *Front Mol Neurosci* **4**, 51 (2011).

26. Lewis, P.W. *et al.* Inhibition of PRC2 activity by a gain-of-function H3 mutation found in pediatric glioblastoma. *Science* **340**, 857-861 (2013).

27. Funato, K., Major, T., Lewis, P.W., Allis, C.D. & Tabar, V. Use of human embryonic stem cells to model pediatric gliomas with H3.3K27M histone mutation. *Science* **346**, 1529-1533 (2014).

28. Hindson, C.M. *et al.* Absolute quantification by droplet digital PCR versus analog real-time PCR. *Nat Methods* **10**, 1003-1005 (2013).

29. McKenna, A. *et al.* The Genome Analysis Toolkit: a MapReduce framework for analyzing next-generation DNA sequencing data. *Genome Res* **20**, 1297-1303 (2010).

30. Li, H. & Durbin, R. Fast and accurate short read alignment with Burrows-Wheeler transform. *Bioinformatics* **25**, 1754-1760 (2009).

31. Li, H. *et al.* The Sequence Alignment/Map format and SAMtools. *Bioinformatics* **25**, 2078-2079 (2009).

32. Wang, K., Li, M. & Hakonarson, H. ANNOVAR: functional annotation of genetic variants from high-throughput sequencing data. *Nucleic Acids Res* **38**, e164 (2010).

33. Shi, Y. & Majewski, J. FishingCNV: a graphical software package for detecting rare copy number variations in exome-sequencing data. *Bioinformatics* **29**, 1461-1462 (2013).

34. Nadaf, J., Majewski, J. & Fahiminiya, S. ExomeAI: detection of recurrent allelic imbalance in tumors using whole-exome sequencing data. *Bioinformatics* **31**, 429-431 (2015).

35. Deshwar, A.G. *et al.* PhyloWGS: reconstructing subclonal composition and evolution from whole-genome sequencing of tumors. *Genome Biol* **16**, 35 (2015).

36. Love, M.I., Huber, W. & Anders, S. Moderated estimation of fold change and dispersion for RNA-seq data with DESeq2. *Genome Biol* **15**, 550 (2014).

37. Dobin, A. *et al.* STAR: ultrafast universal RNA-seq aligner. *Bioinformatics* **29**, 15-21 (2013).

38. Carbon, S. *et al.* AmiGO: online access to ontology and annotation data. *Bioinformatics* **25**, 288-289 (2009).

39. Huang da, W., Sherman, B.T. & Lempicki, R.A. Systematic and integrative analysis of large gene lists using DAVID bioinformatics resources. *Nat Protoc* **4**, 44-57 (2009).

Acknowledgements

The authors would like to express their sincere gratitude toward all the patients' families, as well as the staff at the McGill University and Genome Quebec Innovation Centre for excellent technical expertise, library preparation and sequencing. This work was performed within the context of the I-CHANGE consortium (International Childhood Astrocytoma iNtegrated Genomics and Epigenomics consortium) and supported by funding from Genome Canada, Genome Quebec, The Institute for Cancer Research of the Canadian Institutes for Health Research (CIHR) McGill University and the Montreal Children's Hospital Foundation. It was also supported by UL1TR000075 and KL2TR000076 from the NIH National Center for Advancing Translational Sciences, The Smashing Walnuts Foundation (Middleburg, VA), The Zickler Family Foundation (Chevy Chase, MD), Goldwin Foundation (St. Lincoln, NE), The Piedmont Community Foundation (Middleburg, VA), The Musella Foundation (Hewlett, NY), The Mathew Larson Foundation (Franklin Lake, NJ), and Brain Tumor Foundation for Children (Atlanta, GA). N. Jabado is a member of the Penny Cole lab and the recipient of a Chair de Recherche from le Fond de Recherche en Sante au Quebec. J. Majewski holds a Canada Research Chair (tier 2). T. Gayden is supported by a studentship from the CIHR. D. Bechet is supported by a studentship from the T.D trust/Montreal Children's Hospital Foundation. E. Panditharatna is a predoctoral student in the Molecular Medicine Program of the Institute for Biomedical Sciences at the George Washington University. This work is from a dissertation to be presented to the above program in partial fulfillment of the requirements for the Ph.D. degree.

Author contributions

E.P., L.M., D.F., D.B. performed experiments, E.P., L.M., H.N., R.L., T.G., M.K., E.I.H., M.O. S.P.C., A.S., C.Y.H., J.K., N.J., J.N., J.M. analyzed the data and produced figures and tables, K.L.L., B.E., W.J.I., A.S.M., C.S., K.E.W., R.J.P. provided tissue samples. N.J., J.N. and J.M. provided project leadership and designed the study.

Conflict of Interest

The authors declare that they have no competing financial interests.

Figure Legends

Figure 1. Oncogenic alterations in 41 sub-regions from nine DIPG patients from whole exome sequencing data. Samples representing different anatomical locations within each patient are represented in columns. The mutations (in rows) were selected based on published datasets in pediatric glioblastoma and specifically DIPG. Mutations were divided into two subgroups; driver mutations which are essential for tumor initiation/maintenance and accessory driver mutations, which can further promote and accelerate tumor growth, but are not absolutely essential for tumor initiation or maintenance.

Figure 2. Selected examples of clonal evolution within DIPG tumors. Left: histograms show the raw allele frequencies (whole exome sequencing data) for each somatic mutation in different autopsy regions within each tumor. Red: ubiquitous mutations across regions; yellow: mutations shared in at least two regions; blue: mutations seen in only one region. Right: Phylogenetic trees constructed from the mutation allele frequencies of deep amplicon sequencing data showing the order of evolution along with support probabilities (upper portions of graphs) and clonal mixing proportions within samples (lower portions). For clarity, only mutations selected to be likely oncogenic are shown. A) DIPG5: a rare case harboring both TP53 and PPM1D mutations, which are generally found to be mutually exclusive. PPM1D and TP53 mutations occur in distinct clones and are both secondary to H3K27M. ATRX is also secondary and subclonal. B) DIPG6: while it is impossible to resolve the order of H3/TP53/ATRX mutations' appearance, PIK3CA is clearly sub-clonal and appears in the later stages of evolution within this tumor. C) DIPG2: the H3.1 K27M and ACVR1 main driver mutations are ubiquitous, occur at similar frequencies across all samples, and their mutations order cannot be resolved. Conversely, other accessory

driver mutations are clearly secondary in order of appearance, and are present only in distinct subclones.

Figure 3. Tumor spread in DIPG. A) Tumor spread in DIPG2 in the thalamus, cerebellum and brain stem. Tumor extension in thalamus harbors secondary mutation PIK3CA, MAX, and PTEN which indicates late spread from both Pons 1 and Pons 2. Extension towards cerebellum is relatively early in the tumor evolution as it lacks secondary mutations found in the primary tumor and other brainstem spread. B) Evolution of tumor in patient DIPG3. Autopsy revealed two morphologically and histologically distinct regions of the tumor, indicated as DIPG3 Pons 1 (low-grade) and DIPG3 Pons 2 (high-grade). Exome sequencing identified 11 SNVs and several large scale CNAs common to both regions. Shared alterations included H3.2 K27M and ACVR1 G328V mutations that are likely the main driver mutations in this patient. The analysis also indicated a clear clonal substructure of the two regions, with 18 SNVs and 1 CNA found only in DIPG3 Pons 1, and 11 SNVs and 3 CNAs unique to DIPG3 Pons 2. Intriguingly, DIPG3 Pons 2 carries the activating PIK3CA H1047R mutation, which occurs early in the evolution of this sub-clone judging by its high allelic frequency. PIK3CA H1047R is associated with multi-potency[24] and PI3K activation with angiogenesis and growth and this mutation likely contributes to tumor aggressiveness and high-grade features of Pons 2 compared to Pons 1 in DIPG3. *Scale bar 500 μm.*
